# Supplementary material for: Effectiveness of knowledge brokering and recommendation dissemination for influencing healthcare resource allocation decisions: A cluster randomised controlled implementation trial
Source: PLoS Med. 2021 Oct 22;18(10):e1003833. doi: 10.1371/journal.pmed.1003833 (PMC8570499; doi:10.1371/journal.pmed.1003833)
Supplement: S1 Text — (PDF) [file pmed.1003833.s003.pdf]

Sarkies *et al. Implementation Science* (2018) 13:60
https://doi.org/10.1186/s13012 018 0752 7

Implementation Science

STUDY PROTOCOL Open Access

# Implementation of evidence-based weekend service recommendations for allied health managers: a cluster randomised controlled trial protocol

Mitchell N. Sarkies[1*], Jennifer White[2], Meg E. Morris[3,4], Nicholas F. Taylor[3,5], Cylie Williams[6], Lisa O'Brien[7], Jenny Martin[8], Anne Bardoel[9], Anne E. Holland[10], Leeanne Carey[11,12], Elizabeth H. Skinner[2], Kelly Ann Bowles[1], Kellie Grant[1], Kathleen Philip[13] and Terry P. Haines[14]

## Abstract

**Background:** It is widely acknowledged that health policy and practice do not always reflect current research evidence. Whether knowledge transfer from research to practice is more successful when specific implementation approaches are used remains unclear. A model to assist engagement of allied health managers and clinicians with research implementation could involve disseminating evidence based policy recommendations, along with the use of knowledge brokers. We developed such a model to aid decision making for the provision of weekend allied health services. This protocol outlines the design and methods for a multi centre cluster randomised controlled trial to evaluate the success of research implementation strategies to promote evidence informed weekend allied health resource allocation decisions, especially in hospital managers.

**Methods:** This multi centre study will be a three group parallel cluster randomised controlled trial. Allied health managers from Australian and New Zealand hospitals will be randomised to receive either (1) an evidence based policy recommendation document to guide weekend allied health resource allocation decisions, (2) the same policy recommendation document with support from a knowledge broker to help implement weekend allied health policy recommendations, or (3) a usual practice control group. The primary outcome will be alignment of weekend allied health service provision with policy recommendations. This will be measured by the number of allied health service events (occasions of service) occurring on weekends as a proportion of total allied health service events for the relevant hospital wards at baseline and 12 month follow up.

**Discussion:** Evidence based policy recommendation documents communicate key research findings in an accessible format. This comparatively low cost research implementation strategy could be combined with using a knowledge broker to work collaboratively with decision makers to promote knowledge transfer. The results will assist managers to make decisions on resource allocation, based on evidence. More generally, the findings will inform the development of an allied health model for translating research into practice.

**Trial registration:** This trial is registered with the Australian New Zealand Clinical Trials Registry (ANZCTR) (ACTRN12618000029291). Universal Trial Number (UTN): U1111 1205 2621.

**Keywords:** Implementation, Research, Evidence, Allied health, Weekend, Decision making, Evidence informed decision making, Resource allocation, Knowledge broker, Recommendation

* Correspondence: mitchell.sarkies@monash.edu
[1]Monash University and Monash Health Allied Health Research Unit, Kingston Centre, 400 Warrigal Road, Heatherton, Victoria 3192, Australia
Full list of author information is available at the end of the article

© The Author(s). 2018 **Open Access** This article is distributed under the terms of the Creative Commons Attribution 4.0 International License (http://creativecommons.org/licenses/by/4.0/), which permits unrestricted use, distribution, and reproduction in any medium, provided you give appropriate credit to the original author(s) and the source, provide a link to the Creative Commons license, and indicate if changes were made. The Creative Commons Public Domain Dedication waiver (http://creativecommons.org/publicdomain/zero/1.0/) applies to the data made available in this article, unless otherwise stated.

## Background

### Background and rationale

One of the challenges of evidence-based healthcare worldwide is to effectively and efficiently translate the findings of research into practice. Patient outcomes, patient satisfaction, cost-effectiveness, and quality outcomes benefit from evidence-informed decision-making [1–3]. Local healthcare policies that foster the timely translation of research findings to behaviour change can facilitate evidence-based practice [4–7]. In some cases, allied health policy and practice do not directly reflect current research evidence [8–12]. The delay in the translation of research into practice has also been documented for the medical [13] and nursing [14] professions, where it can take over 10 years for new scientific discoveries to enter day to day clinical practice [15, 16].

Allied health professionals generally have positive attitudes towards evidence-informed decision-making [8–10, 17–19]. Research receptivity and capability among allied health professionals are also influenced by organisational characteristics such as team dynamics, a culture of acceptance or resistance to change, and managerial support [20–22]. Allied health policy-makers and managers can influence these organisational factors and facilitate the translation of research into policy and practice [23]. However, they do not always have the training or access to knowledge transfer resources to assist them to engage effectively with research implementation [24, 25].

Implementation research has sought to develop strategies to reduce the gap between scientific evidence and practice [26]. A recently published systematic review identified 32 studies examining a number of different research implementation strategies for allied health professionals [27]. Education as a single strategy was most frequently evaluated, yet was not always successful in facilitating desired behaviour change [27]. Isolated educational strategies targeting individual professionals may not always meet the needs of complex organisational structures and multiple levels of decision-making involved in adopting an innovation. Providing resources to assist evidence-informed healthcare policy and management decisions may also facilitate behaviour change [28]. Slade et al. [3] highlighted the importance of allied health managers in fostering a research culture to embed evidence-based practice.

Developing evidence-based policy recommendations for allied health decision-makers has the potential to increase engagement with research implementation [29]. Short documents, which communicate key research findings in an accessible format, are one of the few research implementation strategies evaluated for use by resource allocation decision-makers, such as allied health managers [3, 23, 30, 31]. Single research implementation strategies have been reported as less successful than multifaceted approaches in some settings [32].

More interactive strategies may complement the provision of evidence-based policy recommendations, particularly in health services without a strong research culture [33]. One such interactive strategy is the use of knowledge brokers to work collaboratively with stakeholders, promoting the transfer and exchange of information [34]. Indeed, in Canada, many public health organisations have adopted knowledge broker roles as linking agents and capacity builders [35]. This is despite limited evidence to support their benefits [36]. Further high quality empirical research is needed to evaluate this particular implementation resource that could be provided to allied health policy-makers and managers to support the translation of research into practice.

### Implementation context

One area of allied health policy and practice that could better align with the current research evidence is the provision of allied health services to hospital wards during weekends. Routinely throughout the world, allied health services including physiotherapy, speech and language therapy, occupational therapy, social work, nutrition and dietetics, and podiatry, are delivered Monday to Friday. In some parts of the world, allied health services are also provided on Saturdays and Sundays [37–40]. Saturday physiotherapy services are the most common form of allied health provided outside business hours internationally [39, 41]. Only 30% of sub-acute hospitals provide weekend physiotherapy, despite evidence suggesting the provision of after-hours or weekend rehabilitation improves outcomes in the sub-acute rehabilitation setting [41–43]. Research implementation strategies could inform weekend allied health resource allocation decisions to better align policy and practice with contemporary research evidence.

### Objectives

This protocol outlines the design and methods for a multi-centre cluster randomised controlled trial to evaluate the success of select research implementation strategies for promoting evidence-informed weekend allied health resource allocation decisions by hospital managers. The implementation strategies will guide allied health managers in deciding how resources for provision of allied health services on weekends can be allocated between general medical and surgical, and sub-acute rehabilitation wards.

The resource allocation decision will be based on the following question: “How should resources for the provision of allied health services on weekends be allocated between general acute medical/surgical and sub-acute rehabilitation wards?”

## Methods

### Trial design

This multi-centre study will be evaluated using a three-group matched (based on health service regional status) parallel cluster randomised controlled trial. A three-group design will allow the comparison of two different research implementation strategies with a control. Stratification will be based on self-reported health service geographical classification as metropolitan or regional (including rural and remote), and clustering will occur at the level of weekend allied health resource allocation decision-making within each health service (e.g. health service level or hospital level).

This evaluation will be based on the Kirkpatrick Evaluation Model Hierarchy framework, which has four outcome levels that are designed as a sequence of ways to evaluate training programs [44, 45]. This study will focus on behaviour change outcomes in the third category.

### Study setting

The study sample will be drawn from Australian and New Zealand hospitals. Public or private, acute and sub-acute hospitals providing inpatient allied health services will be eligible for inclusion, with a representation of hospitals from both metropolitan and regional geographic classifications sought. Specialist hospitals such as maternity hospitals, paediatric hospitals, cancer centres, mental health and palliative care hospitals will be excluded. These hospitals will be excluded as no research regarding weekend allied health provision has been identified in these settings.

### Eligibility criteria

Allied health managers responsible for weekend allied health resource allocation decisions will be eligible for inclusion. All allied health professions currently providing an inpatient service to acute general medical and surgical wards, and sub-acute rehabilitation wards are eligible. A representation of the different allied health professions (e.g. physiotherapy, occupational therapy, speech pathology, dietetics, podiatry, psychology, exercise physiology, and social work) will be sought. We shall include those who currently provide weekend allied health services as well as those who do not currently provide services, but could potentially introduce these services.

### Interventions

Three intervention groups will be compared: control strategy group, implementation strategy group 1, and implementation strategy group 2. A summary of the intervention conditions described according to the Template for Intervention Description and Replication (TIDieR) guidelines is provided in Table 1 [46]. The two research implementation strategies were designed according to factors perceived to be associated with effective strategies and the inter-relationship between these factors to establish an imperative for change, build trust, develop a shared vision, and action a change mechanism, supported by effective employment of communication strategies and provision of resources to support change [23].

#### *Control strategy group*

The control strategy group will involve a 12-month wait-list for the provision of an evidence-based policy recommendation document at trial completion. This group will involve usual practice conditions, as per each health services usual decision-making process.

#### *Implementation strategy group 1: provision of an evidence-based policy recommendation document*

Participants will be provided with an electronic evidence-based policy recommendation document via email after random group allocation. This will have specific recommendations as to how the proportion of total allied health services should be delivered during weekends to align with current research evidence. Project investigators will develop draft recommendations through a consensus building approach based on the results of a systematic review and meta-analysis of the effectiveness and cost-effectiveness of in-patient weekend allied health services for improving patient and health service outcomes. In addition, a key stakeholder committee comprised of health professionals, managers, consumers, carer representatives, policy-makers, and academics will review draft recommendations and provide feedback before document finalisation. The document will be constructed in a simple format based on the Canadian Health Services Research Foundation [47]. This format allows for a one-page outline of key messages that have come from the research, a three-page executive summary, and 25-pages presenting the report findings and methodology.

#### *Implementation strategy group 2: provision of an evidence-based policy recommendation document and access to a knowledge broker*

Participants will be provided with the same electronic evidence-based policy recommendation document as implementation strategy group 1 via email after random group allocation. In addition, participants in implementation strategy group 2 will have access to a knowledge broker who will facilitate the transfer and exchange of relevant information between researchers and healthcare decision-makers to promote evidence-informed decision-making (EIDM) [34, 36]. A single knowledge broker with a Post-Honorary Doctorate (PhD) level qualification, from an allied health professional

**Table 1** Intervention conditions according to the TIDieR guidelines

| TIDieR criteria | Control group | Implementation strategy group 1 | Implementation strategy group 2 |
|---|---|---|---|
| Item 1. "Brief name: provide the name of a phrase that describes the intervention" | Usual practice control group | Evidence based policy recommendation document | Evidence based policy recommendation document and a knowledge broker |
| Item 2. "Why: describe any rationale, theory, or goal of the elements essential to the intervention" | Usual practice is the model of weekend allied health resource allocation decision making at the research location. This serves as a pragmatic reference standard for implementation research | The evidence based policy recommendation document will communicate research findings in an accessible format to facilitate evidence informed decision making [43]. This will be achieved by embedding an understanding of the political context within design, providing quality evidence communicated through a credible messenger, and fostering active engagement and linkages between policy makers and researchers [44]. | The evidence based policy recommendation document will be the same as that provided to implementation strategy group 1. In addition, the knowledge broker will act as an intermediary agent to facilitate the transfer and exchange of relevant information between researchers and healthcare decision makers to promote evidence informed decision making [48, 49]. The knowledge broker will undertake activities focused on identifying and engaging with decision makers, facilitating collaboration, identifying and obtaining relevant information, facilitating development of analytic and in terpretive skills, creating research implementation resources, project coordination, communication and information sharing, network development, evaluating change, and supporting sustainability [48]. |
| Item 3. "What (materials): Describe any physical or informational materials used in the intervention, including those provided to participants or used in intervention delivery or in training of intervention providers. Provide information on where the materials can be accessed (e.g. online appendix, URL)." | There will be no materials provided to the control group during the study period. Participants will be able to use materials ordinarily available for resource allocation decisions at their discretion. | The evidence based policy recommendation document provided will be constructed in a simple 1:3:25 format developed by the Canadian Health Services Research Foundation [55]. It al lows for a one page outline of key messages, a three page executive summary, and 25 pages presenting the report findings and methodology. | Participants will be provided with the same evidence based policy recommendation document as implementation strategy group 1. Participants in implementation strategy group 2 will also be provided with access to a knowledge broker who may deliver educational materials including plain English summaries, slides, and handouts. Scientific abstracts and full text journal articles relevant to the weekend allied health resource allocation decision may also be provided as applicable. |
| Item 4. "What (procedures): Describe each of the procedures, activities, and/or processes used in the intervention, including any enabling or support activities." | Weekend allied health resource allocation decisions will follow usual practice conditions according to pre existing individual and organisational processes. | The evidence based policy recommendation document will be emailed to participants after random group allocation. This document was developed by project investigators through a consensus building approach and reviewed by a key stake holder committee comprised of health professionals, managers, consumers, carer representatives, policy makers, and academics. | The same version of the evidence based policy recom mendation document provided to implementation strategy group 1 will be emailed to participants after random group allocation.<br>The knowledge broker will offer an initial consultation to perform an individual, organisational, and external environment (e.g. government policy) needs assessment, and develop a 12 month plan. One webinar session will be offered within the first 6 months depending on allied health manager availability, and monthly follow up contact will also be offered. |
| Item 5. "Who: For each category of intervention provider (e.g. psychologist, | | A team of tertiary qualified academics, clinicians, and | A team of tertiary qualified academics, clinicians, and policy |

**Table 1** Intervention conditions according to the TIDieR guidelines *(Continued)*

| TIDieR criteria | Control group | Implementation strategy group 1 | Implementation strategy group 2 |
|---|---|---|---|
| nursing assistant), describe their expertise, background and any specific training given." | Participants may consult a variety of individuals at their discretion. | policy makers from healthcare and business management backgrounds in Victoria, Australia produced and endorsed the evidence based policy recommendation document. | makers from healthcare and business management backgrounds in Victoria, Australia produced and endorsed the evidence based policy recommendation document.<br>In addition, one knowledge broker with a PhD level qualification, from an allied health professional background, with research experience, currently employed as a post doctoral research fellow will be recruited for this implementation strategy. |
| Item 6. "How: Describe the modes of delivery (e.g. face to face or by some other mechanism, such as internet or telephone) of the intervention and whether it was provided individually or in a group." | Usual practice conditions may involve participants accessing information via internet, telephone, or face to face when making resource allocation decisions. | An electronic evidence based policy recommendation document will be provided via email. | An electronic evidence based policy recommendation document will be provided via email.<br>The 1:1 initial knowledge broker consultation will be offered via telephone, videoconference, or face to face (where available) as per participant preference. The group based webinar session will be offered via video or audio and follow up contact will be offered via email or telephone (as per participant preference). |
| Item 7. "Where: Describe the type(s) of location(s) where the intervention occurred, including any necessary infrastructure or relevant features." | Usual practice conditions are likely to involve participants making decisions at their place of work. | An electronic version of the evidence based policy recommendation document will be delivered via email. Therefore, participants may be able to access at the location of their choice. This is most likely to be accessed at their place of work, in an acute or sub acute hospital. | An electronic version of the evidence based policy recommendation document will be delivered via email. Therefore, participants may be able to access at the location of their choice. This is most likely to be accessed at their place of work, in an acute or sub acute hospital. The knowledge broker contact will occur via webinar, telephone, or email. Therefore, participants may be able to access at the location of their choice. This is most likely to be accessed at their place of work in an acute or sub acute hospital. If the initial consultation can be arranged face to face, this will occur at a location convenient to both the participant and the knowledge broker, most likely at the participant's place of work. |
| Item 8. "When and How Much: Describe the number of times the intervention was delivered and over what period of time including the number of sessions, their schedule, and their duration, intensity or dose." | 12 month wait list of usual practice conditions. The evidence based policy recommendation document will be provided upon study completion. | One evidence based policy recommendation document will be provided to participants after random group allocation for the duration for the 12 month intervention period. | One evidence based policy recommendation document will be provided to participants after random group allocation for the duration for the 12 month intervention period.<br>The knowledge broker will provide one 60 min initial consultation, one 60 min group webinar, and one follow up contact each month for the 12 month intervention period. |
| Item 9. "Tailoring: If the intervention was planned to be personalised, titrated or | Usual practice conditions allow participants to take various | There is no adaptation planned for the evidence based policy | There is no adaptation planned for the evidence based policy |

**Table 1** Intervention conditions according to the TIDieR guidelines *(Continued)*

| TIDieR criteria | Control group | Implementation strategy group 1 | Implementation strategy group 2 |
|---|---|---|---|
| adapted, then describe what, why, when, and how." | approaches when making resource allocation decisions. These can be altered at participant discretion as per organisation policy and practice. | recommendation document during the study period. | recommendation document during the study period. The knowledge broker role is iterative in nature. Interaction will be tailored to the needs of the participants at the discretion of the knowledge broker based on their professional judgement. |
| Item 10. "Modifications: If the intervention was modified during the course of the study, describe the changes (what, why, when, and how)." | Not applicable for protocol | Not applicable for protocol | Not applicable for protocol |
| Item 11. "How Well (planned): If intervention adherence or fidelity was assessed, describe how and by whom, and if any strategies were used to maintain or improve fidelity, describe them." | Adherence or fidelity will not be assessed in the usual practice control group, as no implementation strategy will be provided during the study period. | Whether or not participants read the evidence based policy recommendation document will be explored in the 12 month follow up qualitative interviews. | Whether or not participants read the evidence based policy recommendation document will be explored in the 12 month follow up qualitative interviews. Adherence to the knowledge broker component of the implementation strategy group 2 intervention will be monitored via the knowledge broker diary kept for the 12 month period. |
| Item 12. "How Well (actual): If intervention adherence or fidelity was assessed, describe the extent to which the intervention was delivered as planned." | Not applicable for protocol | Not applicable for protocol | Not applicable for protocol |

background, with research experience, currently employed as a post-doctoral research fellow will be recruited. The knowledge broker will offer a 60-min initial consultation with the allied health manager on a one-on-one basis via telephone, videoconference, or face to face (where able) to perform an individual, organisational, and external environment (e.g. government policy) needs assessment. Where required, the knowledge broker will assist development of a 12-month plan to address individual and organisational capacity for evidence-informed decision-making. One 60-min group-based webinar session will be offered within the first 6 months of the intervention period depending on allied health manager availability. Follow-up contact will be offered on a monthly basis via email or telephone, according to the manager's preference, throughout the 12-month intervention period. Knowledge broker dosage (frequency, intensity, time, and type) was based on the description of a knowledge broker role implemented as part of a randomised controlled trial evaluating three knowledge translation strategies by Dobbins et al. [36]. The knowledge broker will follow an iterative process, with prompting questions informed by the COM-B (capacity, opportunity, motivation, and behaviour) model [48]. The number and format of contacts between the allied health manager and knowledge broker will record engagement with the knowledge broker implementation strategy.

## Outcomes

### *Primary outcome: alignment of weekend allied health service provision with policy recommendations at 12-month follow-up*

Allied health service events (occasions of service) occurring during weekends, as a proportion of total allied health service events for the relevant hospital wards over a 1 month period will be used to determine alignment with the policy recommendation. This information will be collected at baseline for the preceding calendar month and the same corresponding calendar month at the 12-month follow-up. Allied health service events will be defined as per the 2017 National Allied Health Data Working Group (NAHDWG) endorsed National Allied Health Best Practice Data Sets (Additional file 1). Where data relating to allied health service events during weekends and weekdays is not available, data relating to allied health staffing levels or budgetary allocations during the weekend as a proportion of total allied health staffing levels or budgetary allocations for the relevant wards at each hospital for the preceding month will be used. Each participant cluster (hospital/health service) will receive a single classification as either (1) practice fully aligned with policy recommendation for both acute and sub-acute hospital wards, (2) practice partially aligned with policy recommendations (e.g. if practice on acute wards is completely aligned with the policy recommendation but not on sub-acute wards, and vice versa), or (3) practice not aligned with policy recommendation.

#### *Secondary outcome 1: mean hospital length of stay at 12-month follow-up*

The mean hospital length of stay for relevant wards over the 1 month period preceding random group allocation, and the same corresponding month 12 months later. Hospital length of stay is a key driver of hospital efficiency [49–52] and provides a measure of benefit or non-inferiority for weekend allied health provision [37, 38, 53].

#### *Secondary outcome 2: opportunity cost to make the decision during the intervention period at 12-month follow-up in AUD$ (time to make decision, resources used, and knowledge broker time attributable to each participant in implementation strategy group 2)*

Participants will record self-reported time (person-hours) taken to make the resource allocation decision, and any resources used (e.g. librarian to conduct literature search). For implementation strategy group 2, the self-reported data will be combined with time contributed by the knowledge broker to each decision-maker. The opportunity costs involved in making a decision (e.g. staff time and resources used) will be captured using interviews with allied health managers and logs of staff time recorded by research personnel. Measures of staff time will be valued using market salary rates in AUD$, with a 33% salary "on-cost" loading to account for allowances, leave, and other employee entitlements. Understanding the cost and benefits of providing research implementation strategies can assist healthcare governance agencies making implementation resource allocation decisions.

#### *Process measures*

Semi-structured interviews performed by a researcher who has not been involved in delivering the intervention at 12-month follow-up will be used to explore participant experiences concerning: (1) perceptions of the trustworthiness and sufficiency of evidence of the evidence base to guide clinical practice in this area of allied health service delivery, (2) the sources of information relied upon by allied health managers when deciding upon the model of weekend allied health service delivery they used in acute and sub-acute hospital wards and why they chose to use those sources, (3) perceived most influential source of information encountered by allied health managers when deciding upon the model of weekend allied health service delivery they used in acute and sub-acute hospital wards and why they thought this source was the most influential, and (4) perceived potential improvements to the intervention received and how it was provided. This information will be used to inform future allied health research implementation strategies.

#### *Qualitative measure: perceived risks, barriers, and facilitators to adopting evidence-based policy recommendations at 12-month follow-up (implementation strategy groups 1 and 2) and during knowledge broker interactions (implementation strategy group 2 only)*

Semi-structured interviews will explore what participants perceived as being the risks, barriers, and facilitators encountered in adopting or not adopting the policy recommendation. Control group participants will not be invited to participate in this final qualitative interview.

### Participant timeline

The Consolidated Standard of Reporting Trials (CONSORT) study flow diagram is provided in Fig. 1 [54]. Baseline data collection and implementation strategy group allocation are planned to occur following the participant information and consent process. A 12-month period will then be provided between initial implementation strategy provision and follow-up to allow sufficient time, on pragmatic grounds, to initiate changes required to align weekend allied health resource allocation with the evidence-based policy recommendation. Follow-up data will then be collected after the 12-month intervention period upon trial completion. The Standard Protocol Items: Recommendations for Interventional Trials (SPIRIT) flow diagram schedule of enrolment, interventions, and assessment procedures is described in Table 2 [55].

### Sample size

The sample size estimate was calculated based on the units of assessment of the primary outcome 'alignment of weekend allied health service provision with policy recommendations at 12-month follow-up'. The most conservative unit of assessment for this outcome will be clustered at individual hospitals/health services with geographically distinct decision makers. We therefore conducted our power analysis at this "cluster-level" rather than at the ward-level or individual decision-maker level (as one hospital/health service may have multiple decision makers involved). A sample size of 25 clusters (hospitals/health services) per group will provide greater than 80% power under the assumption that 50% of participants in an intervention group and 10% in the control group will completely align with the policy recommendation. We will aim to recruit 27 clusters (hospitals/health services) per group to allow for approximately 5–10% loss to follow-up in each group. Assumptions regarding statistical power and expected loss to follow-up were derived from a similar randomised controlled trial by Dobbins et al. [33]. Based on data from a survey of physiotherapy services provided outside business hours in Australian hospitals by Shaw et al. [41], and allied health staffing levels for health service

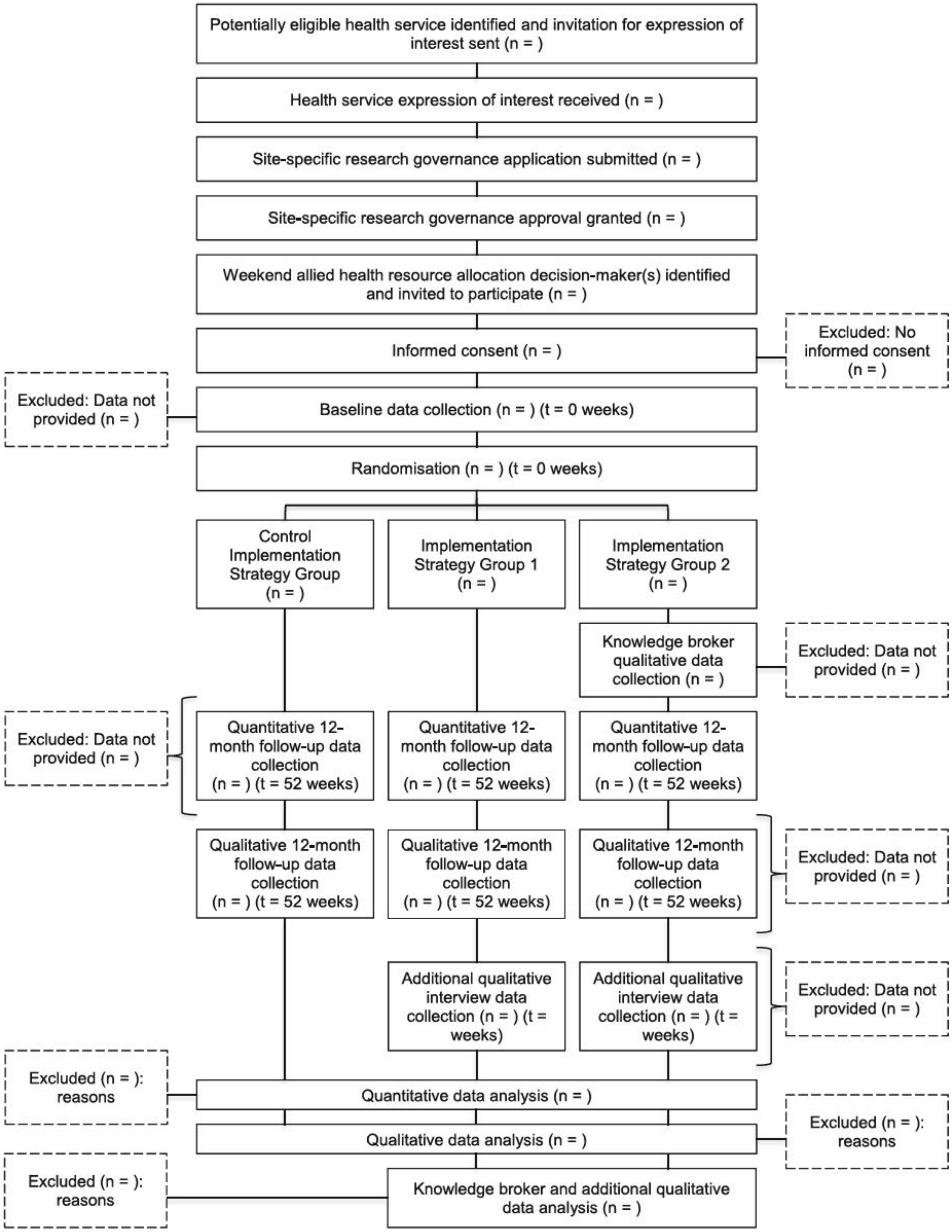


**Fig. 1** CONSORT study flow diagram

**Table 2** SPIRIT flow diagram: schedule of enrolment, interventions, and assessment procedures

| Activity | Study period | | | |
|---|---|---|---|---|
| | T −1<br>Pre-allocation | T 0<br>Baseline | T 1<br>Intervention period | T 2<br>12-month follow-up |
| Enrolment: | | | | |
| Organisation expression of interest | X | | | |
| Site-specific research governance approval | X | | | |
| Participant information and consent | X | | | |
| Random group allocation | | X | | |
| Interventions: | | | | |
| Control group: *usual practice* | | — | — | — |
| Implementation strategy 1: *evidence-base policy recommendation document* | | — | — | — |
| Implementation strategy 2: *evidence-base policy recommendation document and knowledge broker* | | — | — | — |
| Outcomes: | | | | |
| Data collection (all groups): | | | | |
| - Demographic data | X | | | X |
| - Allied health service events | X | | | X |
| - Hospital length of stay | X | | | X |
| - Cost to make decision | | | | X |
| - Semi-structured interviews | | | | X |
| Knowledge broker qualitative data collection (implementation strategy group 2 only) | | | X | |
| Qualitative interview data collection (implementation strategy groups 1 and 2) | | | | X |

inpatients in Victoria [56], it is anticipated that none of the recruited health services will be completely aligned with the evidence-based policy recommendation at baseline. This analysis was performed using Stata 13 (StataCorp, 2013. Stata Statistical Software: Release 13. College Station; TX: StataCorp LP).

#### Recruitment

Project investigators will identify potentially eligible hospitals/health services using pre-existing professional networks and publicly available resources (e.g. government websites). Members of the research team will contact the allied health management either face-to-face, via telephone, or email and provide information regarding the study. The allied health management will then be asked if they support the research being conducted at their organisation. If support is provided, the potential participant/s responsible for weekend allied health resource allocation decisions at the health service will be identified via the allied health management. Potential participants (weekend allied health resource allocation decision-makers) will be contacted, provided with information on the study, and written informed consent will be sought for participation.

#### Assignment and concealment of implementation strategy group allocation

Study investigators will consult with health service representatives to determine decision-making structure for weekend allied health services within their health network. Some health services in Australia are comprised of multiple geographically separated hospitals that report to the same board but have independent decision-making processes in relation to weekend allied health service provision decisions. They will be treated as separate units of recruitment and randomisation, where health service representatives report that decision-making for geographically distinct hospitals is independent and that they anticipate ability to prevent contamination of the intervention between sites within their network. Units of randomisation (hospitals or health services) will then be stratified according to geographic location. A random number sequence will be generated using an online software application (Sealed Envelope

Ltd. 2018. Create a blocked randomisation list. [Online] Available from: https://www.sealedenvelope.com/simple-randomiser/v1/lists), incorporating permuted blocks of randomly selected sizes of three, six, or nine, and stratified according to metropolitan or regional status. This random number sequence will be generated and held by a single investigator (TPH) in a secure location so that investigators conducting recruitment and data collection are blinded to the allocation status of participating sites and the allocation of the next site to be recruited.

## Blinding

Participant weekend allied health decision-makers will not be blinded to group allocation. It will be clear to participants which implementation strategy group they have been allocated due to the nature of the trial, thus it is not possible to blind participants. In order to maintain allocation concealment, site allocation to implementation strategy group 2 will only be revealed to the knowledge broker once the site has been recruited and baseline data collected. Investigators performing data collection will be blinded to participant implementation strategy group allocation for the duration of the study. Investigators performing qualitative interviews will not be blinded. The trial data analyst will be blinded to group allocation. Three mock codes representing different sequence allocation will be used to blind the statistician conducting the final quantitative data analysis from the identity of each hospital/health service and the implementation strategy group allocation.

## Data collection

Project investigators will collect the primary outcome data by requesting an allied health activity statistics report for the relevant wards at each hospital from the weekend allied health decision-maker/s at each health service. It will be requested that this report contains the minimum variables: number of allied health service events, date of each allied health service event, as well as hospital and ward location of each allied health service event. Where these data are not available, allied health staffing levels or budgetary allocations during weekdays and weekends for the relevant wards at each hospital in the preceding month will be requested. The mean hospital length of stay for relevant wards will be collected as reported by the hospital electronic patient management systems. Previous research has shown this data collection method for hospital length of stay provides completeness of data capture when compared to other methods [49]. Allied health managers will be encouraged to record the amount of time (person-hours) taken and other resources used (e.g. librarian) to make the resource allocation decision in a log. For implementation strategy group 2, these data will be combined with knowledge broker records of the amount of time attributed to each participant during the intervention period. Project investigators will perform audio-recorded semi-structured interviews either face to face, via telephone, or videoconference as per participant preference. Qualitative data from knowledge broker conversations will be audio-recorded, and regular communication (e.g. email, phone, online forums) with the knowledge broker will be captured.

## Analysis

### *Quantitative*

Primary outcome: alignment with policy recommendation will be analysed with pairwise comparisons (intervention group 1 vs control, intervention group 2 vs control, and intervention group 1 vs intervention group 2) performed using the sign rank test for ordinal data among matched pairs. This primary analysis will be conducted at both the cluster level and ward level. Analysis will be undertaken according to 'as randomised' (intention-to-treat) principles. Where it is identified that participants have moved between clusters allocated to different intervention groups during the study period, we will undertake a contamination-adjusted intention-to-treat analysis.

### *Qualitative*

Semi-structured interview and data from knowledge broker interactions will be transcribed verbatim, with identifying data removed. An inductive thematic analysis process including constant comparison will used to analyse qualitative data [57]. Rigour in this qualitative study will be ensured by the strategies of immersion in data, reflexive analysis, memo writing, peer debriefing, and consensus coding between team members [58].

### *Economic evaluation*

The economic analysis will calculate the "incremental cost per additional cluster and ward that completely align with the policy recommendation" of implementation strategy group 1 vs control, and implementation strategy group 2 vs control. The opportunity costs involved in making a decision, captured using interviews with decision-makers and research personnel logs of staff time involved, will be valued using market salary rates with a 33% on cost loading to account for leave and other employee entitlements. This analysis will be a trial-based evaluation. The analysis will then be fed into a net-benefit analysis which will incorporate data relating to the amount of allied health events captured at the baseline and 12-month follow-up assessments and changes in hospital length of stay. These data will then be modelled into a 5-year time horizon assuming that weekend service levels at the 12-month follow-up assessment are maintained 5 years into the future. One-way sensitivity analyses will be conducted to model the

effects of having different numbers of allied health managers involved with making this decision at both the cluster and ward level.

### Monitoring study conduct

Monitoring of study progress will be performed at regular meetings between study investigators. Strategic governance oversight will review study recruitment progress, quality of data collection and management, and the occurrence of any unintended effects identified throughout study conduct. Adjustments shall be made to aspects of trial conduct as necessary; however, funding sources will not be involved in study monitoring or decisions regarding adjustment of trial conduct. Data collection at baseline and 12-months will provide an opportunity for monitoring of study progress. The knowledge broker shall also be able to provide feedback to the wider research team as to the study progress in implementation strategy group 2. There is no planned interim analysis, as only one study follow-up period has been planned.

### Ethics and dissemination

#### *Research ethics approval*

Approval to conduct this study has been obtained from the Monash Health Research Ethics Committee (HREC/17/MonH/44) and has been registered with the Australian New Zealand Clinical Trials Registry (ANZCTR) (ACTRN12618000029291). Universal Trial Number (UTN): U1111-1205-2621. Site specific research governance approval will be sought from each requesting health service upon review of the study protocol, participation information and consent form, and other requested documents (including subsequent modifications). Subsequent to initial review and approval, investigators will make safety and progress reports as requested.

#### *Protocol amendments*

Amendments to the study protocol will require approval from study investigators and the human research ethics committee. Any amendments will be communicated via trial registration updates, and reported in any published manuscripts associated with the study as necessary.

#### *Consent*

Potential participating allied health decision-makers will be provided with information regarding the study via the participant information and consent form. They will be provided with the opportunity to discuss the project with study investigators and time to consider their response. Return of a signed participant information and consent form will constitute informed consent for study participation. A copy of the signed participant information and consent form will be provided to the participant, and the researchers will retain a copy for their records. The Version 2, Master Participation Information and Consent Form (PICF) is presented in Additional file 2.

#### *Confidentiality*

The researchers will conduct themselves in accordance with the Declaration of Helsinki and the Principles of Ethical Conduct outlined in the National Statement on Ethical Conduct in Research Involving Humans [59]. All forms where the participant is identified (e.g. consent forms) will be kept in a locked filing cabinet in a lockable room, accessible by only the research team. Electronic data will be stored in password access folders on Monash University 'LabArchives'. The details of data storage will be made available to participants who will not be identifiable in any literature published from the findings of this study.

#### *Access to data*

Information relating to the participation in the trial will not be available to any persons outside the study team. De-identified results data can be made available upon request to study investigators.

#### *Ancillary and post-trial care*

Usual practice control group participants will receive the evidence-based policy recommendation document upon trial conclusion.

#### *Dissemination policy*

A forum for allied health managers will be organised for the purpose of communicating the findings from this research. The results from the research will be reported in scientific journals and presented at conferences and workshops with personal information omitted. Participants will be advised they may request a copy of any results once available. De-identified data will be made available upon request to study investigators. Authorship eligibility will be determined according to the International Committee of Medical Journal Editors (ICMJE) recommendations.

## Discussion

Implementing research evidence into health policy and practice is actively promoted [23, 60]. The continuous process of disinvesting from low value healthcare practice and reinvesting in new approaches that are more efficacious, accurate, and safe, requires the integration of local expertise with the best available external evidence from systematic research [61]. Research in the allied health professions has identified the benefits of many healthcare interventions, such as strength and functional sensory discrimination training to reduce impairments for patients following stroke [62–66]. Innovations in allied health service delivery have also been made to

improve access to, and reduce cost of interventions. Recent studies suggest that rehabilitation and exercise programs for chronic health conditions may be equally effective when delivered in home-based settings compared with centre-based settings, providing a potential alternative for those who cannot access centre-based programs [67–69]. The benefits of these research findings are clear. Yet, in order to produce desired outcomes at scale, evidence must be disseminated and implemented across healthcare organisations [70, 71].

Increased pressure on healthcare organisations to improve access, quality and cost of care has led to the identification of strategies to reduce the gap between research and practice [72]. As allied health professionals generally have positive attitudes towards evidence-informed decision-making [8–10, 17–19], strategies targeted at changing reactions, beliefs, and knowledge may not address the needs of decision-makers. Instead, the research implementation strategies described in our randomised controlled trial protocol aim to complement existing evidence-informed decision-making processes in allied health. Stroke rehabilitation is one of many areas where allied health has led the implementation of research into policy and practice [73]. Consensus implementation statements based on systematic review evidence [74] and clinical champions (diffusion fellows) [75] have been identified as some of the best methods for implementing stroke rehabilitation evidence into practice [73, 76]. While our strategies (evidence-based policy recommendation and knowledge brokerage) share similarities with consensus statements and clinical champions, these approaches have not yet been evaluated in a randomised controlled setting.

## Conclusion

Evidence-based policy recommendation documents communicate key research findings to the healthcare community in an accessible format. This comparatively low-cost research implementation approach can be combined with using a knowledge broker to work collaboratively with decision-makers to promote the transfer and exchange of information. The results from this study may also inform the development of a model for translating research into practice for allied health settings.

## Additional files

**Additional file 1:** Definition allied health service event. (DOCX 72 kb)

**Additional file 2:** Master Participant Information and Consent Form Version 2, 20/09/2017. (DOCX 38 kb)

### Abbreviations

ANZCTR: Australian New Zealand Clinical Trials Registry; COM B: Capacity, opportunity, motivation, and behaviour; EBDM: Evidence based decision making; EviTAH: Evidence Translation in Allied Health; ICMJE: International Committee of Medical Journal Editors; NAHDWG: National Allied Health Data Working Group; PhD: Post Honorary Doctorate; PICF: Participant Information and Consent Form; TIDieR: Template for Intervention Description and Replication; UTN: Universal Trial Number

### Acknowledgements

We wish to thank Monash University, Monash Health, and the Victorian Department of Health and Human Services for providing the support for this project. We thank members of the Evidence Translation in Allied Health (EviTAH) project: Prof. Terry P Haines, Prof. Meg E. Morris, Prof. Leeanne Carey, Prof. Nicholas Taylor, Prof. Anne Holland, Prof. Anne Bardoel, Prof. Jenny Martin, Dr. Cylie Williams, A/Prof Lisa O'Brien, Dr. Elizabeth H Skinner, Dr. Jenni White, and Ms. Kath Philip for their contributions.

### Funding

This study is funded by a partnership grant from the National Health and Medical Research Council (NHMRC) Australia (APP1114210), and the Victorian Department of Health and Human Services. The funding arrangement ensured there was no role of the study funder in study design, collection, management, analysis, and interpretation of data, writing the report, and decision to submit for publication.

### Authors' contributions

TH, MS, JW, MM, NT, AH, LC, CW, AB, JM, LOB, ES, KAB, KG, and KP were responsible for the overall design of this study protocol. MS addressed ethical considerations, trial registration, recruitment, and data collection approach, with additional input from TH, JW, and KAB. TH designed interventions, with refinement from MS and JW. Intervention development was led by JW and MS with further input from TH, MM, NT, AH, LC, CW, AB, JM, LOB, ES, KAB, and KP. TH and MS selected outcomes, and TH planned statistical analysis. MS wrote the first draft manuscript and MS, TH, JW, MM, NT, AH, LC, CW, AB, JM, LOB, ES, KAB, KG, and KP contributed to writing the final versions of the manuscript. All authors read and approved the final manuscript.

### Authors' information

Mitchell Sarkies is a Physiotherapist from Melbourne, Victoria, Australia, with an interest in translating research into practice. He is currently a Ph.D. candidate at Monash University.

### Ethics approval and consent to participate

Not applicable.

### Competing interests

Project investigators declare that they have no competing interests.

## Publisher's Note

Springer Nature remains neutral with regard to jurisdictional claims in published maps and institutional affiliations.

### Author details

[1]Monash University and Monash Health Allied Health Research Unit, Kingston Centre, 400 Warrigal Road, Heatherton, Victoria 3192, Australia. [2]Monash University and Monash Health Allied Health Research Unit, Kingston Centre, 400 Warrigal Road, Heatherton, Victoria 3202, Australia. [3]La Trobe Centre for Sport and Exercise Medicine Research, La Trobe University, Bundoora 3086, Australia. [4]North Eastern Rehabilitation Centre, Healthscope Australia, Melbourne, Australia. [5]Allied Health Clinical Research Office, Eastern Health, Box Hill 3128, Australia. [6]Peninsula Health, 4 Hastings Rd, Frankston, Victoria 3199, Australia. [7]Department of Occupational Therapy, Monash University, Building G, McMahons Road, Frankston, Victoria 3199, Australia. [8]School of Arts, Social Sciences and Humanities, Swinburne University, Hawthorn Campus, John St, Hawthorn, Victoria 3122, Australia. [9]Department of Management and Marketing, Swinburne University, BA 1224 Hawthorn Campus, John St, Hawthorn, Victoria 3122, Australia. [10]Alfred Health and La Trobe University, 99 Commercial Rd, Melbourne 3004, Australia. [11]Occupational Therapy, School of Allied Health, La Trobe University, Bundoora, Victoria 3086, Australia. [12]Florey Institute of Neuroscience and Mental Health, Neurorehabilitation and Recovery, Melbourne Brain Centre, 245 Burgundy Street, Heidelberg, Victoria 3084, Australia. [13]Department of

Health and Human Services, Melbourne, Victoria, Australia. [14]Monash University, Level 3, Building G, Peninsula Campus, McMahons Rd, Frankston, Victoria 3199, Australia.

Received: 2 April 2018 Accepted: 18 April 2018
Published online: 24 April 2018

## References

1. Grol R. Successes and failures in the implementation of evidence based guidelines for clinical practice. Med Care. 2001;39(8):Il 46 54.
2. Woolf SH, Grol R, Hutchinson A, Eccles M, Grimshaw J. Potential benefits, limitations, and harms of clinical guidelines. BMJ. 1999;318(7182):527 30.
3. Slade SC, Philip K, Morris ME. Frameworks for embedding a research culture in allied health practice: a rapid review. Health Res Policy Syst. 2018;16(1):29. https://doi.org/10.1186/s12961 018 0304 2.
4. Hyder AA, Bloom G, Leach M, Syed SB, Peters DH, Future Health Systems: Innovations for E. Exploring health systems research and its influence on policy processes in low income countries. BMC Public Health. 2007;7:309. https://doi.org/10.1186/1471 2458 7 309.
5. Lane H, Sarkies M, Martin J, Haines T. Equity in healthcare resource allocation decision making: a systematic review. Soc Sci Med. 2016;175:11 27. https://doi.org/10.1016/j.socscimed.2016.12.012.
6. Haines TP, Foster MM, Cornwell P, Fleming J, Tweedy S, Hart A, et al. Impact of enhanced primary care on equitable access to and economic efficiency of allied health services: a qualitative investigation. Aust Health Rev. 2010; 34(1):30 5.
7. Labonté R. Health systems governance for health equity: critical reflections. Revista de salud pública. 2010;12:62 76.
8. Harding KE, Porter J, Horne Thompson A, Donley E, Taylor NF. Not enough time or a low priority? Barriers to evidence based practice for allied health clinicians. J Contin Educ Health Prof. 2014;34(4):224 31.
9. Lizarondo L, Grimmer Somers K, Kumar S. A systematic review of the individual determinants of research evidence use in allied health. J Multidiscip Healthc. 2011;4:261 72. https://doi.org/10.2147/JMDH.S23144.
10. Jette DU, Bacon K, Batty C, Carlson M, Ferland A, Hemingway RD, et al. Evidence based practice: beliefs, attitudes, knowledge, and behaviors of physical therapists. Phys Ther. 2003;83(9):786 805.
11. Stevenson K, Lewis M, Hay E. Does physiotherapy management of low back pain change as a result of an evidence based educational programme? J Eval Clin Pract. 2006;12(3):365 75.
12. Salls J, Dolhi C, Silverman L, Hansen M. The use of evidence based practice by occupational therapists. Occup Ther Health Care. 2009;23(2):134 45.
13. Hanney SR, Castle Clarke S, Grant J, Guthrie S, Henshall C, Mestre Ferrandiz J, et al. How long does biomedical research take? Studying the time taken between biomedical and health research and its translation into products, policy, and practice. Health Res Policy Syst. 2015;13(1):1.
14. Wallin L. Knowledge translation and implementation research in nursing. Int J Nurs Stud. 2009;46(4):576 87. https://doi.org/10.1016/j.ijnurstu.2008.05.006
15. Westfall JM, Mold J, Fagnan L. Practice based research "Blue Highways" on the NIH roadmap. JAMA. 2007;297(4):403 6.
16. Morris ZS, Wooding S, Grant J. The answer is 17 years, what is the question: understanding time lags in translational research. J R Soc Med. 2011;104(12): 510 20.
17. Metcalfe C, Lewin R, Wisher S, Perry S, Bannigan K, Moffett JK. Barriers to implementing the evidence base in four NHS therapies: dietitians, occupational therapists, physiotherapists, speech and language therapists. Physiotherapy. 2001;87(8):433 41.
18. Bennett S, Tooth L, McKenna K, Rodger S, Strong J, Ziviani J, et al. Perceptions of evidence based practice: a survey of Australian occupational therapists. Aust Occup Ther J. 2003;50(1):13 22.
19. Iles R, Davidson M. Evidence based practice: a survey of physiotherapists' current practice. Physiother Res Int. 2006;11(2):93 103.
20. Döpp CM, Graff MJ, Rikkert MGO, van der Sanden MWN, Vernooij Dassen MJ. Determinants for the effectiveness of implementing an occupational therapy intervention in routine dementia care. Implement Sci. 2013;8(1):1.
21. Skinner EH, Hough J, Wang YT, Hough CR, Southby A, Snowdon DA, et al. Physiotherapy departments in Australian tertiary hospitals regularly participate in and disseminate research results despite a lack of allocated staff: a prospective cross sectional survey. Physiother Theory Pract. 2015; 31(3):200 6.
22. Skinner EH, Williams CM, Haines TP. Embedding research culture and productivity in hospital physiotherapy departments: challenges and opportunities. Aust Health Rev. 2015;39(3):312 4.
23. Sarkies MN, Bowles K A, Skinner EH, Haas R, Lane H, Haines TP. The effectiveness of research implementation strategies for promoting evidence informed policy and management decisions in healthcare: a systematic review. Implement Sci. 2017;12(1):132. https://doi.org/10.1186/s13012 017 0662 0.
24. Borkowski D, McKinstry C, Cotchett M, Williams C, Haines T. Research culture in allied health: a systematic review. Aust J Prim Health. 2016;22(4):294 303.
25. Williams C, Miyazaki K, Borkowski D, McKinstry C, Cotchet M, Haines T. Research capacity and culture of the Victorian public health allied health workforce is influenced by key research support staff and location. Aust Health Rev. 2015;39(3):303 11.
26. Grimshaw JM, Eccles MP, Lavis JN, Hill SJ, Squires JE. Knowledge translation of research findings. Implementation Sci. 2012;7(1):50.
27. Scott SD, Albrecht L, O'Leary K, Ball G, Hartling L, Hofmeyer A, et al. Systematic review of knowledge translation strategies in the allied health professions. Implement Sci. 2012;7(1):70.
28. Damschroder LJ, Aron DC, Keith RE, Kirsh SR, Alexander JA, Lowery JC. Fostering implementation of health services research findings into practice: a consolidated framework for advancing implementation science. Implement Sci. 2009;4(1):50. https://doi.org/10.1186/1748 5908 4 50.
29. Grimshaw JM, Thomas RE, MacLennan G, Fraser C, Ramsay CR, Vale L, et al. Effectiveness and efficiency of guideline dissemination and implementation strategies. Health Technol Asses. 2004;8(6):iii v. 1 72
30. Beynon P, Chapoy C, Gaarder M, Masset E. What difference does a policy brief make? Full report of an IDS, 3ie, Norad study. New Delhi: Institute of Development Studies and the International Initiative for Impact Evaluation (3ie); 2012. http://www.3ieimpact.org/media/filer public/2012/08/22/fullreport what difference does a policy brief make 2pdf adobe acrobat pro.pdf.
31. Jones N, Walsh C. Policy briefs as a communication tool for development research: Overseas development institute (ODI). London; 2008.
32. LaRocca R, Yost J, Dobbins M, Ciliska D, Butt M. The effectiveness of knowledge translation strategies used in public health: a systematic review. BMC Public Health. 2012;12:751. https://doi.org/10.1186/1471 2458 12 751.
33. Dobbins M, Hanna SE, Ciliska D, Manske S, Cameron R, Mercer SL, et al. A randomized controlled trial evaluating the impact of knowledge translation and exchange strategies. Implement Sci. 2009;4(1):1 16. https://doi.org/10.1186/1748 5908 4 61.
34. Bornbaum CC, Kornas K, Peirson L, Rosella LC. Exploring the function and effectiveness of knowledge brokers as facilitators of knowledge translation in health related settings: a systematic review and thematic analysis. Implement Sci. 2015;10(1):1 12. https://doi.org/10.1186/s13012 015 0351 9.
35. Dagenais C, Laurendeau M C, Briand Lamarche M. Knowledge brokering in public health: a critical analysis of the results of a qualitative evaluation. Eval Program Plann. 2015;53:10 7. https://doi.org/10.1016/j.evalprogplan.2015.07.003
36. Dobbins M, Robeson P, Ciliska D, Hanna S, Cameron R, O'Mara L. A description of a knowledge broker role implemented as part of a randomized controlled trial evaluating three knowledge translation strategies. Implement Sci. 2009;4 https://doi.org/10.1186/1748 5908 4 23.
37. Haines TP, O'Brien L, Mitchell D, Bowles K A, Haas R, Markham D, et al. Study protocol for two randomized controlled trials examining the effectiveness and safety of current weekend allied health services and a new stakeholder driven model for acute medical/surgical patients versus no weekend allied health services. Trials. 2015;16(1):1.
38. Haines T, Bowles K, Mitchell D, O'Brien L, Markham D, Plumb S, et al. Impact of disinvestment from weekend allied health services across acute medical and surgical wards: 2 stepped wedge cluster randomised controlled trials. PLoS Med. 2017;14(10):e1002412.
39. Campbell L, Bunston R, Colangelo S, Kim D, Nargi J, Hill K, et al. The provision of weekend physiotherapy services in tertiary care hospitals in Canada. Physiother Can Physiother. 2010;62(4):347 54. https://doi.org/10.3138/physio.62.4.347.
40. Haas R, et al. Early commencement of physical therapy in the acute phase following elective lower limb arthroplasty produces favorable outcomes: a systematic review and meta analysis examining allied health service models. Osteoarthritis Cartilage. 2016;24(10):1667 81.
41. Shaw KD, Taylor NF, Brusco NK. Physiotherapy services provided outside of business hours in Australian hospitals: a national survey. Physiother Res Int. 2013;18(2):115 23.

42. Scrivener K, Jones T, Schurr K, Graham PL, Dean CM. After hours or weekend rehabilitation improves outcomes and increases physical activity but does not affect length of stay: a systematic review. J Phys. 2015;61(2):61 7. https://doi.org/10.1016/j.jphys.2015.02.017.
43. Peiris CL, Taylor NF, Shields N. Extra physical therapy reduces patient length of stay and improves functional outcomes and quality of life in people with acute or subacute conditions: a systematic review. Arch Phys Med Rehabil. 2012;92(9):1490 500. https://doi.org/10.1016/j.apmr.2011.04.005.
44. Kirkpatrick DL. Evaluating training programs: A collection of articles from the Journal of the American Society for Training and Development. American Society for Training and Development, Madison, Wisconsin: American Society for Training and Development; 1975.
45. Kirkpatrick DL. Evaluating human relations programs for industrial foremen and supervisors. Madison: University of Wisconsin; 1954.
46. Hoffmann TC, Glasziou PP, Boutron I, Milne R, Perera R, Moher D, et al. Better reporting of interventions: template for intervention description and replication (TIDieR) checklist and guide. BMJ. 2014;348 https://doi.org/10.1136/bmj.g1687.
47. Davies P. The state of evidence based policy evaluation and its role in policy formation. Natl Inst Econ Rev. 2012;219(1):R41 52.
48. Michie S, van Stralen MM, West R. The behaviour change wheel: a new method for characterising and designing behaviour change interventions. Implement Sci. 2011;6:42. https://doi.org/10.1186/1748 5908 6 42.
49. Sarkies M, Bowles K A, Skinner E, Mitchell D, Haas R, Ho M, et al. Data collection methods in health services research: hospital length of stay and discharge destination. Appl Clin Inform. 2015;6(1):96.
50. Morgan M, Beech R. Variations in lengths of stay and rates of day case surgery: implications for the efficiency of surgical management. J Epidemiol Community Health. 1990;44(2):90 105.
51. Shojania KG, Showstack J, Wachter RM. Assessing hospital quality: a review for clinicians. Eff Clin Pract. 2001;4(2):82 90.
52. Brasel KJ, Lim HJ, Nirula R, Weigelt JA. Length of stay: an appropriate quality measure? Arch Surg. 2007;142(5):461 6.
53. Brusco NK, Shields N, Taylor NF, Paratz J. A Saturday physiotherapy service may decrease length of stay in patients undergoing rehabilitation in hospital: a randomised controlled trial. Aust J Physiother. 2007;53(2):75 81.
54. Moher D, Schulz KF, Altman DG. The CONSORT statement: revised recommendations for improving the quality of reports of parallel group randomized trials. BMC Med Res Methodol. 2001;1(1):2. https://doi.org/10.1186/1471 2288 1 2.
55. Chan A W, Tetzlaff JM, Gøtzsche PC, Altman DG, Mann H, Berlin JA, et al. SPIRIT 2013 explanation and elaboration: guidance for protocols of clinical trials. BMJ. 2013;346 https://doi.org/10.1136/bmj.e7586.
56. Victorian Department of Health and Human Services. Allied Health Staffing levels for health service inpatients in Victoria: Phase 1 Macro Staffing Analysis. In: Services DoHaH, editor. Victoria; 2016.
57. Braun V, Clarke V. Using thematic analysis in psychology. Qual Res Psychol. 2006;3(2):77 101.
58. Morse JM. Critical analysis of strategies for determining rigor in qualitative inquiry. Qual Health Res. 2015;25(9):1212 22.
59. Phelan T. National Statement on ethical conduct in research involving humans. Chisholm Health Ethics Bull. 1999;5(1):1.
60. Orton L, Lloyd Williams F, Taylor Robinson D, O'Flaherty M, Capewell S. The use of research evidence in public health decision making processes: systematic review. PLoS One. 2011;6(7):e21704.
61. Sackett DL, editor. Evidence based medicine: Seminars in perinatology, Elsevier; 1997.
62. Taylor NF, Dodd KJ, Damiano DL. Progressive resistance exercise in physical therapy: a summary of systematic reviews. Phys Ther. 2005;85(11):1208 23. https://doi.org/10.1093/ptj/85.11.1208.
63. Ada L, Dorsch S, Canning CG. Strengthening interventions increase strength and improve activity after stroke: a systematic review. Aust J Physiother. 2006;52(4):241 8. https://doi.org/10.1016/S0004 9514(06)70003 4
64. Harris JE, Eng JJ. Strength training improves upper limb function in individuals with stroke. A Meta Analysis. 2010;41(1):136 40. https://doi.org/10.1161/strokeaha.109.567438.
65. Morris SL, Dodd KJ, Morris ME. Outcomes of progressive resistance strength training following stroke: a systematic review. Clin Rehabil. 2004;18(1):27 39.
66. Leeanne C, Richard M, Thomas AM. SENSe: study of the effectiveness of neurorehabilitation on sensation: a randomized controlled trial. Neurorehabil Neural Repair. 2011;25(4):304 13. https://doi.org/10.1177/1545968310397705.
67. Jansons P, Robins L, O'Brien L, Haines T. Gym based exercise and home based exercise with telephone support have similar outcomes when used as maintenance programs in adults with chronic health conditions: a randomised trial. J Phys. 2017;63(3):154 60. https://doi.org/10.1016/j.jphys.2017.05.018.
68. Zwisler AD, Norton RJ, Dean SG, Dalal H, Tang LH, Wingham J, et al. Home based cardiac rehabilitation for people with heart failure: a systematic review and meta analysis. Int J Cardiol. 2016;221:963 9. https://doi.org/10.1016/j.ijcard.2016.06.207.
69. Holland AE, Mahal A, Hill CJ, Lee AL, Burge AT, Cox NS, et al. Home based rehabilitation for COPD using minimal resources: a randomised, controlled equivalence trial. Thorax. 2016; https://doi.org/10.1136/thoraxjnl 2016 208514.
70. Milat AJ, Bauman A, Redman S. Narrative review of models and success factors for scaling up public health interventions. Implement Sci. 2015;10(1): 1 11. https://doi.org/10.1186/s13012 015 0301 6.
71. Milat AJ, Newson R, King L. Increasing the scale of population health interventions: a guide. North Sydney: NSW Ministry of Health; 2014.
72. Elshaug AG, Moss JR, Littlejohns P, Karnon J, Merlin TL, Hiller JE. Identifying existing health care services that do not provide value for money. Med J Aust. 2009;190(5):269 73.
73. Marion FW, Rebecca JF, Nicol K B, Annie M, Leeanne MC. From what we know to what we do: translating stroke rehabilitation research into practice. Int J Stroke. 2012;8(1):11 7. https://doi.org/10.1111/j.1747 4949.2012.00974.x.
74. Fisher RJ, Gaynor C, Kerr M, Langhorne P, Anderson C, Bautz Holter E, et al. A consensus on stroke: early supported discharge. Stroke. 2011;42(5):1392 7. https://doi.org/10.1161/strokeaha.110.606285.
75. Rowley E, Morriss R, Currie G, Schneider J. Research into practice: Collaboration for Leadership in Applied Health Research and Care (CLAHRC) for Nottinghamshire, Derbyshire, Lincolnshire (NDL). Implement Sci. 2012;7: 40. https://doi.org/10.1186/1748 5908 7 40.
76. Menon A, Korner Bitensky N, Kastner M, McKibbon KA, Straus S. Strategies for rehabilitation professionals to move evidence based knowledge into practice: a systematic review. J Rehabil Med. 2009;41(13):1024 32. https://doi.org/10.2340/16501977 0451.

**Ready to submit your research? Choose BMC and benefit from:**

- fast, convenient online submission
- thorough peer review by experienced researchers in your field
- rapid publication on acceptance
- support for research data, including large and complex data types
- gold Open Access which fosters wider collaboration and increased citations
- maximum visibility for your research: over 100M website views per year

**At BMC, research is always in progress.**

**Learn more** biomedcentral.com/submissions
