# Supplementary material for: Effectiveness of knowledge brokering and recommendation dissemination for influencing healthcare resource allocation decisions: A cluster randomised controlled implementation trial
Source: PLoS Med. 2021 Oct 22;18(10):e1003833. doi: 10.1371/journal.pmed.1003833 (PMC8570499; doi:10.1371/journal.pmed.1003833)
Supplement: S3 Text — (DOCX) [file pmed.1003833.s005.docx]

**Standards for Reporting Implementation Studies: the StaRI checklist for completion**

The StaRI standard should be referenced as: Pinnock H, Barwick M, Carpenter C, Eldridge S, Grandes G, Griffiths CJ, Rycroft-Malone J, Meissner P, Murray E, Patel A, Sheikh A, Taylor SJC for the StaRI Group. Standards for Reporting Implementation Studies [(StaRI) statement](http://www.bmj.com/content/356/bmj.i6795.full). *BMJ* 2017;356:i6795

The detailed Explanation and Elaboration document, which provides the rationale and exemplar text for all these items is: Pinnock H, Barwick M, Carpenter C, Eldridge S, Grandes G, Griffiths C, Rycroft-Malone J, Meissner P, Murray E, Patel A, Sheikh A, Taylor S, for the StaRI group. Standards for Reporting Implementation Studies [(StaRI). Explanation and Elaboration document](http://bmjopen.bmj.com/content/7/4/e013318.full?ijkey=vv4LKZxc25YcLJv&keytype=ref). *BMJ Open* 2017 2017;7:e013318

Notes: A key concept of the StaRI standards is the dual strands of describing, on the one hand, the implementation strategy and, on the other, the clinical, healthcare, or public health intervention that is being implemented. These strands are represented as two columns in the checklist.

| The primary focus of implementation science is the implementation strategy (column 1) and the expectation is that this will always be completed. | The evidence about the impact of the intervention on the targeted population should always be considered (column 2) and either health outcomes reported or robust evidence cited to support a known beneficial effect of the intervention on the health of individuals or populations. |
| --- | --- |

The StaRI standardsrefers to the broad range of study designs employed in implementation science. Authors should refer to other reporting standards for advice on reporting specific methodological features. Conversely, whilst all items are worthy of consideration, not all items will be applicable to, or feasible within every study.

| **Checklist item** | | **Reported on page #** | **Implementation Strategy** | **Reported on page #** | **Intervention** |
| --- | --- | --- | --- | --- | --- |
|  | |  | “Implementation strategy” refers to how the intervention was implemented |  | “Intervention” refers to the healthcare or public health intervention that is being implemented. |
| **Title and abstract** | | | | | |
| Title | **1** | Title  Paragraph 1 | Identification as an implementation study, and description of the methodology in the title and/or keywords | | |
| Abstract | **2** | Abstract  Paragraph 1-3 | Identification as an implementation study, including a description of the implementation strategy to be tested, the evidence-based intervention being implemented, and defining the key implementation and health outcomes. | | |
| **Introduction** | | | | | |
| Introduction | **3** | Introduction  Paragraph 1-2 | Description of the problem, challenge or deficiency in healthcare or public health that the intervention being implemented aims to address. | | |
| Rationale | **4** | Introduction  Paragraph 3 | The scientific background and rationale for the implementation strategy (including any underpinning theory/framework/model, how it is expected to achieve its effects and any pilot work). | Page 6  Introduction  Paragraph 2 | The scientific background and rationale for the intervention being implemented (including evidence about its effectiveness and how it is expected to achieve its effects). |
| Aims and objectives | **5** | Introduction  Paragraph 4 | The aims of the study, differentiating between implementation objectives and any intervention objectives. | | |
| **Methods: description** | | | | | |
| Design | **6** | Methods, Trial design  Paragraph 1-2 | The design and key features of the evaluation, (cross referencing to any appropriate methodology reporting standards) and any changes to study protocol, with reasons | | |
| Context | **7** | Methods, Context  Paragraph 1 | The context in which the intervention was implemented. (Consider social, economic, policy, healthcare, organisational barriers and facilitators that might influence implementation elsewhere). | | |
| Targeted ‘sites’ | **8** | Methods, Participants and setting  Paragraph 1 | The characteristics of the targeted ‘site(s)’ (e.g locations/personnel/resources etc.) for implementation and any eligibility criteria. | Methods, Participants and setting  Paragraph 1 | The population targeted by the intervention and any eligibility criteria. |
| Description | **9** | Methods, Interventions  Paragraph-2 and Table 1 | A description of the implementation strategy | Methods, Interventions  Paragraph-1 and Text box 1 | A description of the intervention |
| Sub-groups | **10** | NA | Any sub-groups recruited for additional research tasks, and/or nested studies are described | | |
| **Methods: evaluation** | | | | | |
| Outcomes | **11** | Methods, Outcomes  Paragraph 1 | Defined pre-specified primary and other outcome(s) of the implementation strategy, and how they were assessed. Document any pre-determined targets | Methods, Outcomes  Paragraph 2 | Defined pre-specified primary and other outcome(s) of the intervention (if assessed), and how they were assessed. Document any pre-determined targets |
| Process evaluation | **12** | NA: reported in  other publications | Process evaluation objectives and outcomes related to the mechanism by which the strategy is expected to work | | |
| Economic evaluation | **13** | NA: reported in other publications | Methods for resource use, costs, economic outcomes and analysis for the implementation strategy | NA: reported in other publications | Methods for resource use, costs, economic outcomes and analysis for the intervention |
| Sample size | **14** | Methods, Statistical methods  Paragraph 3 | Rationale for sample sizes (including sample size calculations, budgetary constraints, practical considerations, data saturation, as appropriate) | | |
| Analysis | **15** | Methods, Statistical methods  Paragraph 1-3 | Methods of analysis (with reasons for that choice) | | |
| Sub-group analyses | **16** | NA | Any a priori sub-group analyses (e.g. between different sites in a multicentre study, different clinical or demographic populations), and sub-groups recruited to specific nested research tasks | | |

| **Results** | | | | | |
| --- | --- | --- | --- | --- | --- |
| Characteristics | **17** | Results, Table 1-2  Paragraph 1 | Proportion recruited and characteristics of the recipient population for the implementation strategy | NA | Proportion recruited and characteristics (if appropriate) of the recipient population for the intervention |
| Outcomes | **18** | Results, Table 4-7  Paragraph 2-3 | Primary and other outcome(s) of the implementation strategy | Results, Table 5-7  Paragraph 3 | Primary and other outcome(s) of the Intervention (if assessed) |
| Process outcomes | **19** | Results, Process outcomes  Paragraph 1  Further detail reported in other publications | Process data related to the implementation strategy mapped to the mechanism by which the strategy is expected to work | | |
| Economic evaluation | **20** | NA: reported in other publications | Resource use, costs, economic outcomes and analysis for the implementation strategy | NA: reported in other publications | Resource use, costs, economic outcomes and analysis for the intervention |
| Sub-group analyses | **21** | NA | Representativeness and outcomes of subgroups including those recruited to specific research tasks | | |
| Fidelity/ adaptation | **22** | Results, Process outcomes  Paragraph 1  Further detail reported in other publications | Fidelity to implementation strategy as planned and adaptation to suit context and preferences | Results, Table 4-7  Paragraph 2-3 | Fidelity to delivering the core components of intervention (where measured) |
| Contextual changes | **23** | NA | Contextual changes (if any) which may have affected outcomes | | |
| Harms | **24** | Results  Paragraph 1 | All important harms or unintended effects in each group | | |
| **Discussion** | | | | | |
| Structured discussion | **25** | Discussion  Paragraph 1-6 | Summary of findings, strengths and limitations, comparisons with other studies, conclusions and implications | | |
| Implications | **26** | Discussion  Paragraph 2-7 | Discussion of policy, practice and/or research implications of the implementation strategy (specifically including scalability) | Discussion  Paragraph 2-7 | Discussion of policy, practice and/or research implications of the intervention (specifically including sustainability) |
| **General** | | | | | |
| Statements | **27** | Methods  Paragraph 1  Declarations  Paragraph 1-2 | Include statement(s) on regulatory approvals (including, as appropriate, ethical approval, confidential use of routine data, governance approval), trial/study registration (availability of protocol), funding and conflicts of interest | | |
