# Supplementary material for: Effectiveness of knowledge brokering and recommendation dissemination for influencing healthcare resource allocation decisions: A cluster randomised controlled implementation trial
Source: PLoS Med. 2021 Oct 22;18(10):e1003833. doi: 10.1371/journal.pmed.1003833 (PMC8570499; doi:10.1371/journal.pmed.1003833)
Supplement: S4 Text — (PDF) [file pmed.1003833.s006.pdf]

Evidence Translation in Allied Health

[redacted]

Weekend Allied Health Services on Acute and Sub-Acute Hospital, Wards, 2018

[redacted]

EVIDENCE-BASED POLICY RECOMMENDATION DOCUMENT:

'EFFECTIVENESS AND ECONOMIC EFFICIENCY OF PROVIDING WEEKEND ALLIED HEALTH SERVICES TO ACUE AND SUB-ACUTE HOSPITAL WARDS'

Evidence Translation in Allied Health 2017

Professor Terry P Haines [1, 2]
Professor Meg E. Morris [3, 3b]
Professor Nicholas Taylor [3 4]
Professor Anne Holland [3 5]
Professor Leeanne Carey [3, 6]
Associate Professor Lisa O'Brien[1]
Dr Elizabeth H Skinner [7]
Dr Cylie Williams [8, 12]
Professor Anne Bardoel [9]
Professor Jenny Martin [10]
Ms Kathleen Philip [11]
Dr Jennifer White [1, 2]
Mr Mitchell N Sarkies [1, 2]
Ms Kellie Grant [1]

Monash University [1], Monash Health [2], La Trobe University[3] & Healthscope [3b], Eastern Health [4], Alfred Health [5], The Florey Institute of Neuroscience and Mental[6], Western Health [7], Peninsula Health [8], Swinburne University [9], RMIT University [10], Victorian Department of Health and Human Services[11], Peninsula Health [12]

This evidence-based policy recommendation document is a publication of the Evidence-Translation in Allied Health (EviTAH) partnership project funded by the National Health and Medical Research Council (NHMRC) and the Victorian Department of Health and Human Services. EviTAH is dedicated to seeking better ways that evidence can be translated into practice in order to achieve real improvements in patient outcomes and efficiencies in service delivery. The views expressed are those of the authors and do not necessarily represent the views of the partnership institutions nor NHMRC.

Effectiveness and economic-efficiency of providing weekend allied health services to acute and sub-acute hospital wards. 2018 Evidence Translation in Allied Health

All rights reserved. This publication may not be re-produced in whole or in part for commercial or non-commercial purposes without express written permission of EviTAH. To seek this permission, please contact: med-evitah@monash.edu.

Please do not share this document as distribution could prevent ability to publish key findings and breach copyright laws.

Evidence Translation in Allied Health
Email: med-evitah@monash.edu

Table of Contents

....1

EVIDENCE-BASED POLICY RECOMMENDATION DOCUMENT: 2

EXECUTIVE SUMMARY 4

Policy context 4

REPORT SUMMARY 5

Background.. 5

Aim.. 5

Methods 5

Results 6

Findings... 6

Recommendations for weekend allied health service delivery changes 6

WHAT DOES THIS MEAN AT A PRACTICAL LEVEL? WHAT DO I DO NOW? 8

_Toc499231584STUDY BACKGROUND 10

METHODS 10

RESULTS.. 14

DISCUSSION 21

Strengths and Limitations.. 22

CONCLUSION 23

REFERENCES 24

FIGURES...28

Appendix 1: NHMRC Evidence Hierarchy: designations of 'levels of evidence' according to type of research question (including explanatory notes).......................37

Appendix 2: Search Term Summary...39

Appendix 3: Inclusion and Exclusion Criteria..41

Appendix 4: Detailed information about included studies.. 42

EXECUTIVE SUMMARY

This evidence-based policy recommendation document was developed by the Evidence Translation in Allied Health (EviTAH) partnership project to assist resource allocation decisions made by managers and policy makers regarding the provision of weekend allied health services on acute and sub-acute hospital wards.

Policy context

The Victorian Department of Health and Human Services Allied Health Categories Position Paper identified more than 25 different allied health therapy professions, emphasising the complexity of resource allocation decisions faced by allied health managers [1]. In the hospital setting; physiotherapy, occupational therapy, speech pathology, social work, podiatry, psychology, and dietetics represent the major allied health service providers. The demands for allied health services, within public and private hospitals, can vary according to the day of the week. In Australia, most therapy services are provided from Monday to Friday, although some allied health services are provided on Saturday and Sunday to maintain patient flow and prevent escalation of care.

Based on results from a systematic review and meta-analyses outlined in this document, the following key recommendations are provided towards the delivery of allied health weekend services in adult, acute and sub-acute wards within public and private hospitals.

- It is recommended that acute general medical and surgical hospital wards do not provide a weekend allied health service staffing model for all patients. Instead, a criterion for “clinical priorities and exceptions” should be developed towards the provision of outreach or on-call allied health services on a case-by-case basis. For example, for deteriorating patients in order to prevent escalation in care. This recommendation **does not apply** to specialist acute wards (e.g. ICU, Emergency, Burns units) that were not involved in the published trials contributing to this systematic review and subsequent recommendation; or have previously established evidence for weekend efficacy.
- It is recommended that sub-acute rehabilitation wards (excluding Geriatric Evaluation and Management) provide a Saturday allied health service consisting of physiotherapy and occupational therapy, or physiotherapy only, in addition to their weekday service model.
- It is recommended that sub-acute rehabilitation wards (excluding Geriatric Evaluation and Management) provide a Sunday physiotherapy service on an individual case-by-case basis for stroke patients and, occasionally, to other patients when a clear need is evident.

Given that each health service is different it is not possible to suggest an ideal service make-up. Rather, individual health services may consider re-allocating existing resources in order to meet the recommendations. This may require the development of a local business case. The Allied Health Resource Allocation Decision Tool (ReAD-iT) is available to assist allied health managers to make evidence-based decisions regarding the effectiveness and cost-effectiveness of health services they are considering allocating resources to. **For more information visit: http://www.readit.health.vic.gov.au/Version2**

REPORT SUMMARY

Background

Allied health professionals, within public and private hospitals, are responsible for deciding upon the timing, quantity and focus of allied health services provided. Allied health professionals also play key roles in determining the types of patients that receive services and the evaluation of therapy outcomes. Allied health managers and professionals commonly have competing demands for limited resources in a range of clinical areas, service settings and patient populations. Further, decisions often need to be made quickly and evidence needs to be provided to justify service delivery. This report focuses on the evidence-base towards the provision of weekend allied health services on acute and sub-acute hospital wards.

Aim

A systematic review and meta-analysis aimed to address the following questions:

1. What is the effectiveness and economic efficiency of providing additional allied health services to patients on acute general medical and surgical hospital wards during weekends?

2. What is the effectiveness and economic efficiency of providing additional allied health services to patients on sub-acute rehabilitation hospital wards during weekends?

Methods

A search strategy was developed to retrieve contemporary literature on the provision of weekend allied health services to acute and sub-acute hospital wards. Articles were screened for inclusion and were assessed for methodological quality and risk of bias by two independent reviewers. Meta-analyses were performed where possible with effect estimates pooled according to the study design (e.g. separate for acute and sub-acute, and randomised and non-randomised study designs). Based on these results draft recommendations were developed towards the delivery of weekend allied health services.

For the purpose of this evidence-based policy recommendation, <u>adult acute general medical and surgical wards</u> were defined as: general medical, general surgical, orthopaedic, vascular, plastics, ear nose and throat (ENT), respiratory, renal, rheumatology, neurological (including stoke units), and gastroenterology hospital wards. Recommendations do not apply to other acute wards, including but not exclusive to: emergency departments, medical assessments units (MAU), coronary care units (CCU), high dependency units (HDU), intensive care units (ICU), burns, spinal, maternity, paediatrics, mental health, and palliative hospital wards. Adult, <u>sub-acute rehabilitation wards</u> were defined as: inpatient rehabilitation (both mixed and condition specific wards). Geriatric Evaluation and Management (GEM) wards were excluded, as there was no published data available towards this clinical area. Recommendations do not apply to other sub-acute wards, including but not exclusive to: chronic and long-term care, transition care, alternative level of care, and extended care wards.

The results of the systematic review and a draft version of recommendations provided within this document were reviewed by EviTAH project committee, and by a committee of stakeholder representatives prior to finalisation. The stakeholder committee included consumer representatives, researchers, and health care manager and clinician representatives across Victorian health services.

Results

A total of 20 eligible studies were identified (38,732 patients) worldwide. Ten randomised controlled trials evaluated the effect of providing allied health services during weekends. Two of these studies were performed in acute general medical and surgical hospital ward settings. Eight were performed in sub-acute rehabilitation hospital wards, including: mixed, orthopaedic, and stroke rehabilitation. Ten non-randomised trials were also identified. Seven of these were cohort studies performed in acute general medical and surgical hospital wards, including: orthopaedic, rheumatology, and stroke. Two cohort studies were performed in mixed rehabilitation sub-acute hospital wards, and one cross sectional study in mixed rehabilitation. There were no identified studies in the GEM setting that met inclusion criteria. The majority of studies were performed in Australia (14 studies) and the most common profession investigated in isolation (9 studies) or combination (20 studies) with other professions was physiotherapy. Most studies examined allied health service models provided between Monday and Friday only, compared to models with additional Saturday +/- Sunday services.

Findings

Acute general medical and surgical wards

A meta-analysis of two stepped-wedge randomised controlled trials (Level I evidence) involving a sequential nonetheless random rollout of an intervention over multiple time periods, in the acute general medical and surgical ward setting (n= 27508), indicated it was unclear whether weekend allied health services significantly changed: hospital length of stay, hospital readmission, the number of adverse events, the number of patients discharged to supported, residential aged-care, and cost to health system per hospital admission [2, 3]. It is worth noting, these studies permitted the limited provision (28 occasions of service over 14 months) of allied health for "clinical exceptions" on an individual case-by-case basis for patients and families [2, 3].

Sub-acute rehabilitation wards

Meta-analysis of three randomised controlled trials (Level I evidence) in the sub-acute rehabilitation setting (n=1437) indicated that the provision of weekend allied health services reduced hospital length of stay by 2.35 days, and may be an economically efficient way to improve function and health-related quality of life outcomes[4-6].

Recommendations for weekend allied health service delivery changes

Acute general medical and surgical wards

- It is recommended that acute general medical and surgical wards do not provide access to weekend allied health services for all patients, as it is unclear whether there are systematic improvements in therapy outcomes, discharge destination, patient safety, readmission rates, and cost of service provision or length of stay, over and above the benefits of Monday to Friday usual care.
- Instead, acute general medical and surgical wards should develop site-specific "clinical priorities and exceptions" towards the provision of weekend allied health services (e.g. on-call or outreach) to manage acutely deteriorating patients and to prevent escalation in care or adverse events.
- It is advised that each health service establish a process for developing evidence-based "clinical priorities and exceptions" that are informed by local health service contextual factors.

For example a study across multiple health services conducted by Haines et al (2017), developed "clinical priorities and exceptions" following input from clinical staff and managers from relevant wards at these health services [3]. These are shown in Table 1. These clinical priorities and exceptions led to less than 0.1% of the total number of allied health occasions of service being provided on the weekend on the wards involved.

Table 1: Clinical priorities and exceptions used in the Haines et al (2017) study [3].

**Profession**	**Clinical scenario**
Physiotherapy	Provision of non-invasive ventilation for patients with severe exacerbation of chronic obstructive pulmonary disease, cardiogenic pulmonary oedema, immunosuppressed patients, and patients at risk of type 1 or type 2 respiratory failure and escalation of care, for example severe exacerbations of chronic obstructive pulmonary disease, immunosuppressed patients with respiratory compromise, or patients with pulmonary complications following abdominal surgery or lung resection surgery [7]
Speech pathology	Provision of a swallow assessment for a patient who has been fasted pending a swallow assessment for up to 72 hours and the 72nd hour occurs on the weekend [8]
Dietetics	Feeding regimen adjustment for a patient who is at risk of re-feeding syndrome and requires an adjustment to their feeding regimen based on pathology results [9]
Social work	To provide bereavement support in cases of stillbirth or foetal death in utero (FDIU). Liaison with government authorities, where a child protection issue has been identified

Sub-acute rehabilitation wards

- It is recommended sub-acute rehabilitation wards be provided with weekend allied health services, in addition to weekday services, with the aim of reducing hospital length of stay, maintaining function and health-related quality of life. Based on trials included in this systematic review that reported favourable results, a suggested weekend allied health-staffing model is shown in Table 2.

Table 2: Suggested weekend allied health staffing for sub-acute rehabilitation wards based on trials included in this systematic review that reported favourable results

Model	Staffing
Physiotherapy + occupational therapy	Sufficient to allow one additional hour of physiotherapy and occupational therapy for each patient on Saturdays. This led to greater than 10% of the total number of physiotherapy occasions of service being delivered on weekends, and greater than 10% of the total number of occupational therapy occasions of service being delivered on weekends.
Physiotherapy only	Sufficient to allow one additional hour of physiotherapy for each patient on Saturdays and/or Sundays. This led to greater than 10% of the total number of physiotherapy occasions of service being delivered on weekends.

The next section provides guidelines to assist health services align with the recommendations.

WHAT DOES THIS MEAN AT A PRACTICAL LEVEL? WHAT DO I DO NOW?

In order to align with the evidence outlined in this systematic review and implement the posited recommendations, each local health service is encouraged to consider the following:

1. How do I interpret this information?

- It is important to read and reflect upon the information presented in this policy recommendation document (Appendix 1 provides an outline of the NHMRC Evidence Hierarchy)
- The aim is to understand how allied health, particularly physiotherapy and occupational therapy roles are currently defined, organised, managed and delivered over the weekend in your health service.
- You may wish to discuss the recommendations further with the allied health heads of department in your health service, or their equivalents.
- The ReAD-iT is a helpful resource designed to help you with your decision making. Visit: http://readit.health.vic.gov.au/Version2

2. How do I act on these recommendations?

- If you decide to implement the recommendations and change allied health service delivery then, depending on your health service, you may need to develop a business case. This often necessitates a local evaluation of the patient mix, caseload, staffing, and workforce profile. In order to assist, we can provide you with access to the data collected relevant to your health service and de-identified, aggregated data from other health services.
- Central to the implementation of the recommendation is the development of allied health "clinical priorities and exceptions" towards the provision of outreach or on-call allied health services on a case-by-case basis. You may wish to involve allied health staff and managers in defining these "clinical priorities and exceptions." Involving staff may help them feel ownership of any changes.
- To help reduce resistance to change it is helpful to ensure that your allied health staff understand the reasons for change and the benefits gained from adopting an evidence based practice model.
- At the ward level, it will be important to involve frontline nurses, other health professionals and support staff as well as nurse managers and physicians. Negotiating with medical practitioners is key, particularly when expectations vary from contemporary evidence.
- Consumer involvement is another consideration. The extent to which you involve patients and their care-givers will vary from site to site.

3. How do I know if I am on the right track?

With the recommendations we have developed metrics based on the key studies included in the systematic review. In the acute hospital trial by Haines et al (2017), weekend allied health services made up approximately 4% of the total occasions of service for allied health over the whole week [3]. When allied health occasions of service on a weekend were re-focused to "clinical priorities and exceptions" these made up less than 0.1% of all allied health occasions of services. In the sub-acute hospital & rehabilitation site study by Pieris et al (2013), weekend occupational therapy made up 15% of total occupational therapy occasions of service and physiotherapy made up 10% of total physiotherapy occasions of service [6]. We therefore suggest the following:

METRICS TO HELP ALIGN WITH THE RECOMMENDATIONS

(a) Based on the data, an allied health service model for acute general medical and surgical wards that is consistent with these evidence-based policy recommendations will have:

- ✓ Between 0% and 0.1% of total allied health occasions of service on these wards being delivered on weekends.

(b) Based on the data, an allied health service model for sub-acute rehabilitation wards (excluding geriatrics evaluation and management) will have:

- ✓ Between 10% and 20% of physiotherapy occasions of service being delivered on weekends
- ✓ Between 10% and 20% of physiotherapy, and between 10 and 20% of occupational therapy services being delivered on weekends.

STUDY BACKGROUND

There are more than 100,000 allied health professionals who work in Victoria, Australia [10] .Allied health professionals comprise 18% of the total health workforce in Australia, and provide an estimated 200 million services annually; delivering interventions that are vital to the functioning of an efficient and effective health system[10]. Specifically, allied health professionals provide treatment to improve patient outcomes, to promote health, wellbeing and social care and to facilitate the economic efficiency of a health care system [10]. Within the Australian system, allied health professionals practice across various settings (acute, sub-acute, rehabilitation, community, and primary care) as well as specialty areas such as geriatrics, neurology, orthopaedics and cardiopulmonary. Allied health professionals are often organised into profession-specific departmental structures (e.g. physiotherapy, occupational therapy, dietetics), which largely allows self-determination regarding models of care delivery.

There is a widespread move towards the implementation of science-led health care practices underpinned by research evidence and guided by principles of safety, effectiveness, patient-centeredness, timeliness, efficiency, and equity [11] . The implementation of evidence-based practice (EBP) amongst allied health professionals is characterised by the integration of best research evidence, clinical expertise, and patient values [12]. There is a growing focus on the review of evidence in the form of clinical guidelines, systematic reviews, meta-analyses, and evidence summaries to arrive at recommendations to guide health professionals in practice [13]. However, this evidence is often in a form that is specific to a given health profession, and focused on a specific intervention and outcome measure [14] which may not directly answer the broader problem faced by managers of allied health departments who often cover patients with needs for diverse health services.

A challenge faced by allied health managers in hospitals in the public and private sectors is to decide how to allocate resources to provision of allied health services on weekends. In Australia 61% of hospitals provided routine physiotherapy on Saturdays, and 45% on Sundays [15], with more provided to metropolitan and acute hospitals than regional and sub-acute hospitals. These findings appear consistent internationally, with a survey of tertiary-care hospitals in Canada reporting that 97% of facilities provided weekend physiotherapy services [16], with substantial diversity in the amount and focus of service delivery. Little is known of the practices of other allied health professions, and the evidence-base to support the benefits of provision of any of these services is presently unclear. Further, enterprise bargaining agreements in Australia [17] presently dictate that staffing of allied health services on weekends are more expensive per hour of provision than weekday services. There can be some difficulties in trying to attract suitably qualified allied health staff into these roles, and the difficulties in performance managing these staff, especially if they only work on weekends [18]. There is a need to better understand the evidence base underpinning allied health weekend service provision and for clear recommendations for practice to be made in light of this evidence base.

METHODS

Approach

Aim: To develop an evidence-based, policy recommendation to assist allied health managers within hospital-based health services.

The objectives of this research were to:

1. Determine the effectiveness and economic-efficiency of providing additional allied health services to adult, acute general medical and surgical hospital wards during weekends.
2. Determine the effectiveness and economic-efficiency of providing additional allied health services to adult, sub-acute rehabilitation wards during weekends.

We separated the objectives into acute and sub-acute hospital service contexts to reflect the difference in the focus and role of allied health professionals in these different areas towards promoting effective and efficient health service delivery.

Study Design
A systematic review and meta-analysis of the literature was used to address these questions.

Following the conduct of the systematic review and meta-analyses, the people who led the review developed a draft set of recommendations. This set of recommendations were discussed and refined by the EviTAH committee. Finally, the recommendations were presented to, discussed and further refined by a committee of stakeholder representatives. This committee included consumer representatives, health care manager representatives, clinician representatives and a health policy maker representative.

Systematic Review: Identification and selection of studies
This systematic review was registered with Prospero (record number 76771) and was conducted and reported in accordance with the Preferred Reporting Items for Systematic Reviews and Meta-Analysis (PRISMA) guidelines. Ovid MEDLINE (all fields), PubMed (all fields), CINAHL (keyword, title, CINAHL subject headings, abstract, and instrumentation fields), Cochrane library (title, abstract, keywords), and Scopus (title, abstract, keywords) were searched from 01/01/2000 to 05/05/2017 in order to retrieve contemporary literature. Terms relevant to the population and intervention were combined and results were limited to English language publications. See Appendix 2 for summary list of search terms.

Electronic database searches were supplemented by crosschecking the reference list of included articles and relevant systematic reviews identified during the screening process. Publication lists from key authors in the field were also hand searched to identify additional studies.

Study selection
Study design
We considered all experimental, quasi-experimental and observational study designs.

Population
Patients admitted to acute general medical and surgical, and sub-acute rehabilitation wards.

Acute general medical and surgical ward inclusion criteria:
General medical, general surgical, medical assessment unit (MAU), orthopaedics, vascular, plastics, ear nose and throat (ENT), respiratory, coronary care unit (CCU), renal, rheumatology, neurology, and gastroenterology.

Acute general medical and surgical ward exclusion criteria:
Emergency department, intensive care unit (ICU), high dependency unit (HDU), burns, spinal, maternity, paediatrics, mental health, and palliative wards.

Sub-acute rehabilitation ward inclusion criteria:
Patients admitted sub-acute rehabilitation wards included: inpatient rehabilitation (both mixed and condition specific wards). However there was no published evidence on GEM wards that could be included in the systematic review.

Sub-acute rehabilitation ward exclusion criteria:
Sub-acute patients admitted to mental health and psychiatric, chronic and long-term care, alternative level of care, and extended care.

Interventions
We included studies that specifically examined services delivered by allied health professionals during weekends (i.e. Saturday and/or Sunday). Weekend services were defined as complementary to the traditional workweek, as per the country the study they were performed. Studies that reported data relating to the provision of additional allied health services as part of changing the timing of commencement, intensity, frequency or duration of service provision with a weekend component were included; but only if data relating specifically to weekend services with appropriate controls could be extracted.

We limited allied health professions to therapy professions as defined by the Victorian Department of Health and Human Services Allied Health Categories Position Paper: occupational therapy (OT), physiotherapy (PT), social work (SW), speech pathology (SP), dietetics (DT), art therapy, chiropractic, exercise physiology, music therapy, oral health (not dentistry), osteopathy, podiatry, psychology, and allied health assistants (AHA's) [1].

Outcomes
Patient and health service outcomes as reported in included studies.

Screening
The web-based application Covidence (Covidence, Melbourne, Victoria, Australia) was used for reference management [19]. Two reviewers (MS and JW) screened titles and abstracts independently (see Appendix 3 for inclusion and exclusion criteria). Studies determined to be potentially relevant or whose eligibility was uncertain were retrieved for full-text review. Where there was any disagreement, a third independent reviewer (KH) was consulted. Two reviewers (MS and JW) then independently assessed the full-text articles to ascertain eligibility for inclusion, with another independent reviewer (RH) deciding on inclusion or exclusion if there was any disagreement in the screening process. Attempts were made to contact authors of studies whose full-text articles were unable to be retrieved, and those that remained unavailable were excluded.

Quality assessment
One (JW) and either of two other reviewers (KH or JB) independently assessed the risk of bias for randomised controlled trials using the Cochrane Collaboration's tool for assessing risk of bias [20], and the Newcastle – Ottawa Quality Assessment Scale for observational studies [21]. Any discrepancy in risk of bias assessment was resolved by discussion, and if discrepancies were unable to be resolved, a fourth independent

reviewer was consulted (RH). Reviewers discussed further areas of potential bias not identified by these tools that were pertinent to this context.

Data extraction
Data were extracted using a data extraction form, developed and piloted for this review. One (JW) and either of two other reviewers (KH or JB) independently extracted data relating to the study details, design, setting, population, intervention, outcomes, and results for all included studies. Discrepancies in extracted data were resolved by discussion, and where agreement could not be reached, a fourth independent reviewer was consulted (MS).

Data analysis
Analysis was performed using Stata (StataCorp, 2013. Stata Statistical Software: Release 13. College Station; TX: StataCorp LP). Relative measures of effect estimates were pooled according to study setting and design (separate for acute and sub-acute settings, and randomised and non-randomised study designs). Random effect meta-analysis accounted for differences in populations, interventions, and outcomes across studies, and was performed where data were available for outcomes evaluated in more than one study.

A majority of our analyses used summative, study-level data. Weighted mean difference (WMD) effect size estimates were used for continuous outcomes where measured and reported in the same units (e.g. length of stay and cost), with the standardised mean difference (SMD) used to estimate effect size for function and quality of life outcomes where measures using difference scales were synthesised. Risk ratios (RR) were used for dichotomous outcomes (e.g. number of adverse events, patients discharged home, and delayed discharge).

Attempts were made to contact authors of studies that did not report enough outcome data for inclusion in the meta-analysis to request additional data. Data were pooled from subgroups to estimate the total population effect size where data were only reported according to sub-groups within an individual trial. Proportion of patients discharged to residential aged-care facility reported as low level and high-level aged care sub-groups was summed to create a total proportion of patients discharged to aged care. Where two control groups were used for non-randomised controlled trials, the first mentioned control group was selected as the comparator. This applied only to observational studies where multiple time periods / cohorts were reported within the study. If study results were reported as medians and range or inter-quartile range, and the mean and standard deviation could not be obtained, means and standard deviation were estimated using the methods of Wan et al [22]. In cases where adequate data were only reported in figures and graphically, a scale ruler was used to determine the mean and standard deviation, or median, range and inter-quartile range. A sensitivity analysis was undertaken to assess the effect of excluding studies where data were estimated with inadequate reporting.

Heterogeneity in study results was represented using the I-squared statistic (I^2), with values over 50% considered substantial [23]. An iterative approach was used to explore possible explanations for heterogeneity by sub-grouping studies according to variables such as: allied health profession, patient population, and potential sources of bias. A formal meta-regression was not planned due to the anticipated low number of studies for inclusion in the meta-analysis.

Two of the studies identified and included in this review were stepped-wedge cluster randomised trials that were conducted in succession at the same research location (hospitals and wards). These were the only randomised trials identified in the acute

setting. We used data available from this study at the participant level (for continuous outcomes) and cluster level (for proportion outcomes) rather than use of summative data. This was done to incorporate the dependency of observations gathered from the same wards between the two trials. Weighted mean difference (WMD) was used for continuous outcomes, and risk difference (RD) for proportional outcomes.

RESULTS

Search results

A total of 3413 titles were identified, with 3405 from the electronic search strategy and 8 from hand searching publication lists of prominent authors. Duplicates (293) were removed using Endnote (n = 224) and Covidence (n = 69), resulting in 3120 titles remaining for screening [Figure 1]. After title and abstract screening, 72 full-text articles were considered potentially eligible for inclusion. The full-text for each publication was then retrieved and screened, with 52 articles removed (most common reason being duplicate publication, n=20), leaving 20 studies (n = 19 articles) included in this review.

Characteristics of included studies
Study details are presented in detail in Appendix 4.

Description of study designs used
Ten randomised controlled trials (9 articles) evaluated the effect of providing allied health services during weekends. Two of these studies were performed in acute general medical and surgical hospital ward settings [3]. Eight were performed in sub-acute rehabilitation hospital wards, including: mixed [4, 6, 24-26], orthopaedic [27], and stroke rehabilitation [5, 28]. Ten non-randomised controlled trials were also identified (10 articles). Seven of these were cohort studies performed in acute general medical and surgical hospital wards, including: orthopaedic [29-33], rheumatology [34], and stroke [35]. Two cohort, studies were performed in mixed rehabilitation sub-acute hospital wards [36, 37], and one cross sectional study in mixed rehabilitation [38].

Description of participants and settings
The majority of studies were performed in Australia (n = 14) [3-6, 24-30, 33, 36, 37], followed by England (n = 1) [34], Canada (n = 1) [38], Japan (ν = 1) [35], Singapore (ν = 1) [31], Scotland (ν = 1) [32]. Provision of physiotherapy during weekends was the most examined allied health profession (n = 9) [4, 5, 28-32, 34, 36] followed by physiotherapy and occupational therapy (n = 7) [6, 24-27, 35, 38] physiotherapy, occupational therapy and social work (n = 1) [37];and all professions (physiotherapy, occupational therapy, speech pathology, dietetics, and social work) (n=3) [3, 33].

Description of weekend allied health services investigated
Ten studies compared a Monday to Friday allied health service with a model that incorporated Saturday and Sunday services [3, 5, 28-30, 32-34, 38], eight compared Monday to Friday with an additional Saturday service [4, 6, 24-27, 36, 37], one Monday to Saturday service compared with an additional Sunday service [35], and one compared no weekend allied health with a new stakeholder driven service including Friday, Saturday, Sunday, and Monday services [3]. See Appendix 4 for further detail, including information on service make-up where available.

Risk of bias
We used reliable and validated tools to assess for bias across all included studies. The Cochrane Collaboration tool for assessing risk of bias is presented in Table 2 for the included randomised controlled trials [20]. Eight studies reported adequate methods of random sequence generation [3, 5, 6, 24-28] and not in one [4]. All studies reported adequate allocation concealment, however blinding of participants and personnel was not possible in any of the included studies. Risk of bias for selective reporting was identified in two studies [5, 6], where another study reported further outcomes to be reported in other publications [3]. No potential other sources of bias were identified for any of the included studies.

Non-randomised controlled trials were assessed using the Newcastle - Ottawa quality assessment scale for cohort and case control studies [21]. Eight studies reported adequate methods to control for potential selection risk of bias [29-31, 33-35, 37, 38], with two studies potentially at risk of selection bias [32, 36]. There was adequate reporting of methods to control for potential comparability risk of bias for all studies. Seven studies reported adequate methods to control for potential outcome or exposure risk of bias [29, 31, 33-35, 37, 38], with three studies potentially at risk of outcome bias [30, 32, 36].

Table 2: The Cochrane Collaboration tool for assessing risk of bias

	Random sequence generation	Allocation concealment	Blinding of participants and personnel	Blinding of outcome assessment	Incomplete outcome data	Selective reporting	Other sources of bias
Haines 2017 [3]	✓	✓	✕	?	✓	✓	✓
Peiris 2013 [6]	✓	✓	✕	?	✓	✕	✓
Brusco et a., 2014 [25]	✓	✓	✕	?	✓	✓	✓
Brusco et al., 2015 [26]	✓	✓	✕	?	✓	✓	✓
Brusco et al., 2014B [24]	✓	✓	✕	✕	✓	✓	✓
Peiris et al., 2012 [27]	✓	✓	✕	✕	✓	✓	✓
Brusco 2007 [4]	✕	✓	✕	?	✓	✓	✓
English 2015 [5]	✓	✓	✕	?	✕	✕	✓
English et al., 2014 [28]	✓	✓	✕	✕	✓	✓	✓

✓: indicates low risk of bias, ✕: indicates high risk of bias, and, ?: indicates unclear risk of bias

Table 3: Newcastle - Ottawa quality assessment scale for cohort and case control studies

Author	Selection	Comparability	Outcome
Maidment 2014 [29]	****	*	***
Boxall 2004 [30]	****	*	**
David 2003 [34]	****	*	***
Pua 2011 [31]	****	*	***
Pengus 2015 [32]	***	*	**
Haas 2017 [33]	****	*	***
Kinoshita 2017 [35]	****	*	***
Caruana 2016 [36]	***	*	**
Hakkennes 2015 [37]	****	*	***
DiSotto-Monastero 2012 [38]	****	*	***

NB- the higher the number of stars in each column indicates lower risk of bias. There is a different scale version used for cross-sectional studies, where "outcome" is replaced with "exposure."

EFFECT OF ADDITIONAL WEEKEND ALLIED HEALTH

Effectiveness and economic-efficiency of providing additional weekend allied health services to acute general medical and surgical wards, and sub-acute rehabilitation wards
In the following, we present meta-analyses of randomised controlled trials for each outcome and setting (acute and sub-acute), followed by comments outlining whether results from non-randomised studies and subgroup analyses were concordant with these. We also comment on when statistical heterogeneity is identified, which indicates that the differences between study results are unlikely to be due to random variation alone. Some caution is required in interpreting the result of a meta-analysis when substantial heterogeneity is identified. Absence of sub-headings for each outcome indicates that no studies reported on that outcome within that category of trial type and setting.

Outcome: Hospital Length of Stay

Setting: Acute

Two acute stepped-wedge, randomised controlled trials (n=27,508) were identified that examined this outcome domain in the acute setting [3]. Meta-analysis of individual participant level data in these trials demonstrated no difference between intervention and control conditions for hospital length of stay (WMD 0.08 days; 95% CI -0.15 to 0.32; I^2=98.7%) [Figure 2(a)]. When patient diagnosis was taken into account, heterogeneity levels were reduced and there was no difference in the proportion of patients whose hospital length of stay was longer than their expected length of stay between intervention and control conditions using cluster level data (RD 0.00 days; 95% CI -0.02 to 0.02; I^2=78.5%) [Figure 3]. High levels of heterogeneity in the study results were examined by post-hoc exploratory analysis in Haines et al (2017) suggesting that there was a significant change in hospital length of stay outcomes between control conditions, but when intervention conditions were compared; there was no significant difference between patient hospital length of stay outcomes [3].

Contrast with non-randomised studies and subgroup analyses

These meta-analysis results were somewhat concordant with those involving only non-randomised studies. Meta-analysis (n = 4,676) of six acute non-randomised controlled trials showed no effect of weekend allied health services on hospital length of stay (WMD 0.24 days; 95% CI -0.17 to 0.66; I^2=95.5%[29-32, 34, 35, 39] [Figure 2(b)]. Heterogeneity levels were reduced when sub-group analysis of only total hip arthroplasty patients was performed (WMD 0.08 days; 95% CI -0.12 to 0.29; I^2=23.7%) [29, 30, 32].

Setting: Sub-acute

Meta-analysis (n = 1,437) of three randomised controlled trials conducted in the sub-acute setting indicated that weekend allied health services reduced sub-acute hospital length of stay (WMD 2.35 days; 95% CI 0.45 to 4.24; I^2=0.0%) [Figure 2(c)] [4-6]. There was no evidence of heterogeneity between studies.

Contrast with non-randomised studies and subgroup analyses

The results from the randomised trials in the sub-acute setting were somewhat concordant with non-randomised studies. A meta-analysis (n = 5,012) of three sub-acute non-randomised controlled trials showed a trend towards reduced hospital length of stay in favour of weekend allied health provision (WMD 0.49 days 95% CI -0.87 to 1.85; I^2=82.7%) [Figure 2(d)] [36-38]. High levels of heterogeneity in study results were observed between studies.

Outcome: Hospital Re-Admission

Setting: Acute

Meta-analysis (n = 27,508) of cluster level data from two acute stepped wedge randomised controlled trials showed no difference in the proportion of patients who had an unplanned hospital re-admission within 28-days post hospital discharge between groups receiving and not receiving weekend allied health services (RD 0.01; 95% CI -0.00 to 0.02; I^2=42.6%) [Figure 4][3]. Low levels of heterogeneity in study results were observed within individual analyses.

This was consistent with the results of one non-randomised trial that also demonstrated no difference in unplanned hospital re-admission within 6 weeks of discharge between groups.

Setting: Sub-acute

Meta-analysis was unable to be performed for this outcome in randomised or non-randomised trials in the sub-acute setting. However, one sub-acute randomised control trial reported no difference between intervention and control conditions for hospital re-admission within 30-days post discharge [25].

Outcome: Adverse Events

Setting: Acute

Meta-analysis (n = 27,508) of cluster level data from two acute stepped wedge randomised controlled trials indicated no statistically significant difference in the proportion of patients experiencing adverse events (n=2464) for the events measured (falls, pressure injuries, pulmonary embolism, deep vein thrombosis, rapid response medical team call, transfer to intensive care or high dependency unit, and death) between those receiving weekend allied health and not receiving weekend allied health services (RD 0.00; 95% CI -0.01 to 0.01; I^2=82.7%) [Figure 5(a)] [3]. High levels of heterogeneity in study results were observed within individual analyses. High levels of heterogeneity in the study results were examined by post-hoc exploratory analysis in Haines et al (2017) suggesting that there was a significant change in patients experiencing any adverse event between control conditions, but when intervention conditions were compared; there was no significant difference [3].

Contrast with non-randomised studies and subgroup analyses

This result was concordant with those from two acute non-randomised controlled trials whose meta-analysis (n = 3,348) showed no effect of weekend allied health on the number of adverse events, n=135 (falls, pressure injuries, pulmonary embolism, deep vein thrombosis, rapid response medical team calls, transfer to intensive care or high dependency unit, and deaths) (RR 1.18; 95% CI 0.51 to 2.73; I^2=78.3%) [Figure 5(b)] [33, 35]. High levels of heterogeneity in study results were observed between studies.

Setting: Sub-acute

Meta-analysis (n = 1,437) of three sub-acute randomised controlled trials indicated no difference between weekend and no weekend allied health for the number of adverse events, n=303 (falls, skin tears, infections, re-admission to acute service, and death) (RR 1.13; 95% CI 0.92 to 1.39; I^2=0.0%) [Figure 5(c)] [4-6]. There was no evidence of heterogeneity between studies.

Outcome: Discharge Destination

Setting: Acute

Meta-analysis (n = 27,508) of cluster level data from two acute stepped wedge

randomised controlled trials indicated no difference in the proportion of patients discharged to aged-care between those receiving weekend allied health and not receiving weekend allied health services (RD 0.00; 95% CI -0.00 to 0.01; I^2=31.2%) [Figure 6(a)] [3]. Low levels of heterogeneity in study results were observed within the individual analyses.

Contrast with non-randomised studies and subgroup analyses
The pooled results from the RCTs and non-RCTS were generally discordant (n = 3,588) where a meta-analysis showed patients receiving weekend allied health may have been more likely to be discharged home to private residence from hospital (RR 1.19; 95% CI 1.03 to 1.38; I^2=58.7%) [Figure 6(b)] [Figure 6(b)] [30, 33, 35]. However, high levels of heterogeneity in study results were observed between studies.

Setting: Sub-acute
Meta-analysis was unable to be performed for this outcome in randomised trials in the sub-acute setting. However, one sub-acute randomised control trial reported no difference between intervention and control conditions for the number of patients discharged to aged-care [4].

Contrast with non-randomised studies and subgroup analyses
This result was concordant with those from two sub-acute non-randomised studies (n = 4,476) where a meta-analysis showed no effect of weekend allied health on the relative risk of patients being discharged to aged care (RR 1.00; 95% CI 0.75 to 1.34; I^2=0.0%) [Figure 6(d)] [37, 38].

Outcome: Functional Independence
Setting: Acute
Meta-analysis was unable to be performed for this outcome in randomised trials in the acute setting.

Contrast with non-randomised studies and subgroup analyses
Functional activities of daily living were measured in three acute non-randomised controlled trials [30, 32, 39]. The Barthel Index, ‘days to mobilising with two sticks’, and ‘days to independent mobility’ were transformed to conform to the same effect direction, these were then pooled and categorised as functional activities of daily living outcomes. Meta-analysis of three acute non-randomised studies (n = 1,201) showed no difference in functional independence between those who had weekend allied health services available and those who didn’t (SMD 0.19; 95% CI -0.12 to 0.50; I^2=77.1%) [Figure 7(b)]. Heterogeneity levels were reduced when sub-group analysis of only total hip arthroplasty patients (SMD 0.31; 95% CI 0.12 to 0.51; I^2=0.0%), total knee arthroplasty patients (SMD 0.39; 95% CI 0.15 to 0.64; I^2=0.0%), or studies examining only physiotherapy (no other professions) was performed (SMD 0.34; 95% CI 0.19 to 0.49; I^2=0.0%) (Table 5).

Setting: Sub-acute
The Functional Independence Measure (FIM) was pooled and categorised as a functional activity of daily living outcome measured in three sub-acute randomised controlled trials [4-6]. The Timed Up and Go Test (TUGT), and the Wolf Motor Function Test (WMFT) were transformed to conform to the same effect direction, these were then pooled and categorised as functional mobility outcomes measured in three sub-acute randomised controlled trials [4-6]. Walking speed and 10-Metre Walk Test (10MWT) were transformed to conform to the same effect direction, these were then pooled and categorised as

functional walking speed outcomes measured in three sub-acute randomised controlled trials [4-6]. The Motor Assessment Scale (MAS) and the physical dimension of the Stroke Impact Scale (SIS) were transformed to conform to the same effect direction, these were then pooled and categorised as stroke specific functional outcomes in three sub-acute non-randomised controlled trials [4-6]. Measurement time-points were grouped prior to inpatient discharge.

Meta-analysis (n = 1,437) showed a trend towards improved functional activities of daily living taken prior to hospital discharge in favour of weekend allied health service provision, though this was not statistically significant (SMD 0.09; 95% CI -0.01 to 0.19; I^2=0.0%) [Figure 7(c)]. No difference was identified between intervention and control conditions in meta-analyses of functional mobility (n = 335), walking speed (n = 438), and stroke-specific outcome measures (n = 210).

Non-randomised studies and subgroup analyses
The Functional Independence Measure (FIM) was pooled and categorised as a functional activity of daily living outcome measure in three sub-acute non-randomised controlled trials [36-38]. Results from meta-analysis of randomised controlled trials were somewhat concordant with meta-analysis of three sub-acute non-randomised controlled trials (n = 4,746), which showed no statistically significant difference between weekend and no weekend allied health for functional activities of daily living outcome measures (SMD 0.05; 95% CI -0.17 to 0.28; I^2=89.4%) [Figure 7(d)]. However, high levels of heterogeneity in study results were observed between studies.

Outcome: Health-Related Quality of Life
Setting: Acute
Meta-analysis was not possible for this outcome in the identified randomised trials, as no identified randomised controlled trials reported health-related quality of life in the acute setting. Meta-analysis was also unable to be performed for this outcome in the identified non-randomised trials, as only one was identified in the acute setting. This study demonstrated no difference in health-related quality of life (EQ-5D) at four days postoperatively between the weekend and no weekend allied health groups [33].

Setting: Sub-acute
The EuroQol Five Dimensions questionnaire (EQ-5D) and Assessment of Quality of Life (AQoL) were pooled and categorised as health-related quality of life outcome measures taken prior to discharge in three sub-acute randomised controlled trials [4-6]. Meta-analysis (n = 1,423) indicated a trend towards improved health-related quality of life in favour of weekend allied health service provision (SMD 0.10; 95% CI -0.01 to 0.20; I^2=0.0%), though this result was not statistically significant [Figure 8].

Outcome: Cost of Hospital Care
Setting: Acute
Meta-analysis (n = 27,508) of individual participant level data from two acute stepped wedge randomised controlled trials indicated no difference in patient cost to healthcare system per hospital admission between those receiving and not receiving weekend allied health (WMD $AUD 118; 95% CI -274.50 to 510.50; I^2=98.5%) [Figure 9] [3]. Clinical costing data was captured using routinely applied hospital data collection and resource allocation procedures, largely driven by hospital length of stay and procedures performed, which does not take into consideration cost relative to patient diagnosis. High levels of heterogeneity in the study results were explained by exploratory analysis in Haines et al

(2017) indicating that there was a significant change in total cost favouring the original weekend allied health service delivery model, though these outcomes did not account for differences in patient diagnosis categories between phases [3].

Setting: Sub-acute

Meta-analysis was not possible for this outcome in randomised or non-randomised trials in the sub-acute setting. However, one sub-acute randomised controlled trial reported economic outcomes at 3-month, 6-month and 12-month follow-up [25, 26]. At 3-month follow-up post hospital discharge, there was a mean cost saving of AUD$1,673 favouring weekend allied health service provision. An incremental cost utility ratio saving of AUD$41,825 per quality of life year (QALY) gained, and an incremental cost effectiveness ratio (ICER) found a saving of AUD$16,003 in achieving a minimal clinically important difference (MCID) in functional independence for the group receiving additional weekend allied health service provision. If willingness to pay per QALY gained or MCID in functional independence was zero AUD$, the probability of cost effectiveness was 96% and 95% respectively. A sensitivity analysis removing the Saturday penalty rate salary loading of 50% did not alter the results of the primary analysis. At 6-month follow-up there was a mean cost saving of AUD$6,445 favouring weekend allied health service provision, however this saving was no longer significant at 12-month follow-up. The ICER found a saving of AUD$41,825 (95% CI −2,817 to 74,620) per QALY gained for the weekend allied health group at 6 months.

DISCUSSION

Providing allied health services during weekends is important when interventions dictate the need for allied health, however, there is currently limited evidence regarding the benefits. Diversity and relatively small numbers within specific allied health professions may contribute to limited evidence in some settings. Other barriers include fragmentation, diversity of settings, and the fact that allied health often provide complex multidisciplinary interventions with outcomes that are difficult to measure [40]. Overall, allied health professionals contribute greatly to health care service delivery, and there is growing evidence both by and about allied health professionals however there remains a need for greater focus on resource allocation decision making. This review focussed specifically on the provision of allied health services during weekends on adult, acute and sub-acute wards.

The results of this review pertained primarily to physiotherapy and occupational therapy services, as these were the professions most commonly evaluated in the identified studies. Randomised controlled trial evidence indicated no added benefit for weekend allied health services in the acute general medical and surgical ward setting. However, the finding of no significant effect of weekend allied health services towards reducing hospital length of stay in the acute general medical and surgical ward setting should not be extrapolated to weekday services. Likewise the effect of weekend allied health services was not explored for specialised wards (ICU, high dependency, maternity, or paediatric scenarios) that did not meet our inclusion criteria. Overall, the limited research in weekend allied health provision may indicate that using staff from other areas that do have an adult, weekend allied health service (e.g. ICU, maternity, crisis services) or use an on-call staffing model may be preferable to employing weekend allied health staff in fixed shifts to meet patient needs.

We identified the benefit of providing allied health services during weekends on sub-acute wards. Randomised controlled trial evidence suggested that the provision of

weekend allied health services reduce hospital length of stay and may improve movement, mobility, level of disability and health-related quality of life outcomes.

We acknowledge there is other existing literature exploring after-hours, increased intensity, and therapy delivered by non-allied health professionals, which includes a weekend allied health component. However, many of these studies were not included in this review, as they did not meet inclusion criteria. The reasons for their exclusion include: staff variations (such as nursing and allied health assistants), services provided in outpatient settings or the timing of therapy (no detail specified), and inability to access data specific to the therapy provided on a weekend. For example recent studies include: a systematic review by Pieris et al [41] exploring extra physical therapy in acute and sub-acute conditions; an RCT exploring the provision of early rehabilitation after stroke (AVERT study) [28]; a systematic review by Haas et al [42] exploring elective joint replacement, an RCT by Kimmel et al [43] exploring intensive acute hospital physiotherapy for patients with isolated hip fractures; an RCT by Calthorpe et al [44] exploring intensive physiotherapy for trauma patients; and a quasi-experimental study exploring the effect of an \increased days and both volume and scope of allied health [45].

Our results indicated that the provision of extra therapy sessions on the weekend was not sufficient to improve hospital length of stay in this setting. Considering the increased staffing costs due to weekend penalty rates and the logistical difficulty of staffing allied health professionals during weekends, it needs to be considered whether resources could be used more effectively in other settings. Indeed, future research in the field of weekend allied health service delivery is warranted, particularly in other ward types and when examining higher dosage levels of service delivery and the impact of day of surgery. Given the problems inherent in determining the benefit of weekend allied health, an attractive therapeutic option is to consider the dose of therapy. Increased dose outside of weekend settings has been suggested as an alternative to providing care on weekends [42]. If higher dosage levels can demonstrate an effect on clinical or patient flow outcomes (key studies outlined earlier), they will still need to be justified with research evidence and economic modelling. There is a wide scope for future research in this area to better understand dose-response relationship for allied health interventions to inform future policy, governance and service delivery in Australia.

Strengths and Limitations

Even though randomised controlled trial evidence provides the highest levels of evidence, we also included quasi-experimental studies in this systematic review. The reason for this was that, while RCTs are well designed, they often include large, homogenous patient populations. In contrast, quasi-experimental studies do not have randomly assigned groups; yet can often represent real-life situations since variables are less tightly controlled, thus providing greater understanding on this topic. Further, the included randomised controlled trials were also predominantly conducted in a Melbourne metropolitan setting in Australia. By including data from quasi-experimental studies, we were able to contrast the findings to those from other geographic locations, a process consistent with the Cochrane Collaboration recommendations.

However, we acknowledge that ascertainment bias was a potential source of bias as patients and staff involved in many of the studies could not be blinded as to whether patients were exposed to the weekend allied health service. There was also potential for bias in the cohort studies included in this review as a result of the confounding effects of concurrent interventions. Publication bias through lack of trial registration could have occurred and a control group selection bias might have eventuated where the choice of

comparison dataset was not made prior to collection of that data. Further, we excluded all the studies addressing intensity of therapy in scenarios when we were unable to obtain data weekend data separately from key trials.

CONCLUSION

Favourable benefits of allied health services were evident in the meta-analysis of the literature worldwide. The provision of additional weekend allied health services on sub-acute rehabilitation wards appeared to reduce patient length of stay by more than 2 days, and may be an economically efficient intervention to improve function and health-related quality of life. While there are clear benefits for weekday allied health services on acute general medical and surgical wards, providing weekend allied health to all patients on acute general medical and surgical wards does not appear to improve therapy outcomes, discharge destination, patient safety, readmission rates, or length of stay, over and above the benefits of usual care.

REFERENCES

1. Allied Health: Therapy and Science Disciplines Position Paper. In: Services. DoHH, ed. Victoria, Australia; 2016.

2. Mitchell D, Bowles K, O'Brien L, Markham D, Plumb S, May K, Philip K, Haas R, Sarkies M, Ghaly M, Shackell M, Chiu T, McPhail S, McDermott F, Skinner E, Haines T. *Weekend allied health services for acute medical / surgical patients to improve efficiency and safety of patient care: Report to the Victorian Department of Health and Human Services.* 2017.

3. Haines TP, Bowles K-A, Mitchell D, O'Brien L, Markham D, Plumb S, May K, Philip K, Haas R, Sarkies MN. Impact of disinvestment from weekend allied health services across acute medical and surgical wards: 2 stepped-wedge cluster randomised controlled trials. *PLoS medicine.* 2017;14(10):e1002412.

4. Brusco N, Shields N, Taylor N, Paratz J. A Saturday physiotherapy service may decrease length of stay in patients undergoing rehabilitation in hospital: a randomised controlled trial. *Australian Journal of Physiotherapy.* 2007;53(2):75-81.

5. English C, Bernhardt J, Crotty M, Esterman A, Segal L, Hillier S. Circuit class therapy or seven-day week therapy for increasing rehabilitation intensity of therapy after stroke (CIRCIT): a randomized controlled trial. *International Journal of Stroke.* 2015;10(4):594-602.

6. Peiris C, Shields N, Brusco N, Watts J, Taylor N. Additional Saturday rehabilitation improves functional independence and quality of life and reduces length of stay: a randomized controlled trial. *BMC Medicine.* 2013;11(1):1.

7. Keenan SP, Sinuff T, Burns KE, Muscedere J, Kutsogiannis J, Mehta S, Cook DJ, Ayas N, Adhikari NK, Hand L. Clinical practice guidelines for the use of noninvasive positive-pressure ventilation and noninvasive continuous positive airway pressure in the acute care setting. *Canadian Medical Association Journal.* 2011;183(3):E195-E214.

8. Stroke N. Transient Ischaemic Attack in Over 16s: Diagnosis and Initial Management. *London: NICE.* 2008.

9. Care NCCfA. Nutrition support for adults: oral nutrition support, enteral tube feeding and parenteral nutrition. 2006.

10. Buchan J, Law D. *A review of allied health workforce models and structures.*

11. Baker A. Crossing the quality chasm: a new health system for the 21st century. *BMJ: British Medical Journal.* 2001;323(7322):1192.

12. Lobb R, Colditz G. Implementation science and its application to population health. *Annual Review of Public Health.* 2013;34:235-251.

13. Gopalakrishnan S, Ganeshkumar P. Systematic reviews and meta-analysis: understanding the best evidence in primary healthcare. *Journal of Family Medicine and Primary Care.* 2013;2(1):9.

14. Adams J, Tocchnin L. The impact of allied health professionals in improving outcomes and reducing the cost of treating diabetes, osteoarthritis and stroke. *Canberra: Novartis Pharmaceuticals.* 2015.

15. Shaw KD, Taylor NF, Brusco NK. Physiotherapy services provided outside of business hours in Australian hospitals: a national survey. *Physiotherapy Research International.* 2013;18(2):115-123.

16. Campbell L, Bunston R, Colangelo S, Kim D, Nargi J, Hill K, Brooks D. The provision of weekend physiotherapy services in tertiary-care hospitals in Canada. *Physiotherapy Canada.* 2010;62(4):347-354.

17. Stanton P, Bartram T, Harbridge R. People management practices in the public health sector: developments from Victoria, Australia. *Journal of European Industrial Training.* 2004;28(2/3/4):310-328.

18. Philip K. Allied health: Untapped potential in the Australian health system. *Australian Health Review.* 2015;39(3):244-247.

19. Covidence. https://www.covidence.org/. Accessed 18/11/2016, 2016.

20. Higgins J, Altman D, Gøtzsche P, Jüni P, Moher D, Oxman A, Savović J, Schulz K, Weeks L, Sterne J. The Cochrane Collaboration’s tool for assessing risk of bias in randomised trials. *BMJ.* 2011;343:d5928.

21. Wells G, Shea B, O'Connell D, Peterson J, Welch V, Losos M, Tugwell P. Newcastle-Ottawa quality assessment scale cohort studies; 2014.

22. Wan X, Wang W, Liu J, Tong T. Estimating the sample mean and standard deviation from the sample size, median, range and/or interquartile range. *BMC Medical Research Methodology.* 2014;14:135.

23. Higgins J, Thompson S, Deeks J, Altman D. Measuring inconsistency in meta-analyses. *BMJ.* 2003;327(7414):557-560.

24. Brusco N, Watts J, Shields N, Chan S, Taylor N. Does additional acute phase inpatient rehabilitation help people return to work? A subgroup analysis from a randomized controlled trial. *Clinical Rehabilitation.* 2014;28(8):754-761.

25. Brusco NK, Watts JJ, Shields N, Taylor NF. Are weekend inpatient rehabilitation services value for money? An economic evaluation alongside a randomized controlled trial with a 30 day follow up. *BMC Med.* 2014;12:89.

26. Brusco N, Watts J, Shields N, Taylor N. Is cost effectiveness sustained after weekend inpatient rehabilitation? 12 month follow up from a randomized controlled trial. *BMC Health Services Research.* 2015;15(1):1.

27. Peiris C, Taylor N, Shields N. Additional Saturday allied health services increase habitual physical activity among patients receiving inpatient rehabilitation for lower limb orthopedic conditions: a randomized controlled trial. *Archicw Physical Medicine and Rehabilitation.* 2012;93(8):1365-1370.

28. English C, Bernhardt J, Hillier S. Circuit class therapy and 7-day-week therapy increase physiotherapy time, but not patient activity: early results from the CIRCIT trial. *Stroke.* 2014;45(10):3002-3007.

29. Maidment Z, Hordacre B, Barr C. Effect of weekend physiotherapy provision on physiotherapy and hospital length of stay after total knee and total hip replacement. *Australian Health Review.* 2014;38(3):265-270.

30. Boxall A, Sayers A, Caplan G. A cohort study of 7 day a week physiotherapy on an acute orthopaedic ward. *Journal of Orthopaedic Nursing.* 2004;8(2):96-102.

31. Pua Y, Ong P, Chong H, Lo N. Sunday physiotherapy reduces inpatient stay in knee arthroplasty: a retrospective cohort study. *Archives of Physical Medicine and Rehabilitation.* 2011;92(6):880-885.

32. Pengas I, Khan W, Bennett C, Rankin K. Impact of Weekend Physiotherapy Service on the Cost Effectiveness of Elective Orthopaedic Hip and Knee Arthroplasty. *Open Orthopaedics Journal.* 2015;9:515-519.

33. Haas R, O'Brien L, Bowles K, Haines T. Effectiveness of a pragmatic weekend physical therapy service on short-term outcomes following hip and knee arthroplasty. *Manuscript submitted for publication 2017.* 2017.

34. David C, Price N, Price T, Sheeran T, Mulherin D. Impact of weekend physiotherapy delivery on the throughput of rheumatology inpatients: Feasibility study. *Physiotherapy.* 2003;89(1):25-29.

35. Kinoshita S, Momosaki R, Kakuda W, Okamoto T, Abo M. Association Between 7 Days Per Week Rehabilitation and Functional Recovery of Patients With Acute Stroke: A Retrospective Cohort Study Based on the Japan Rehabilitation Database. *Archives of Physical Medicine and Rehabilitation.* 2017;98(4):701-706.

36. Caruana E, Kuys S, Clarke J, Bauer S. A pragmatic implementation of a 6-day physiotherapy service in a mixed inpatient rehabilitation unit. *Disability and Rehabilitation.* 2016:1-6.

37. Hakkennes S, Lindner C, Reid J. Implementing an inpatient rehabilitation Saturday service is associated with improved patient outcomes and facilitates patient flow across the health care continuum. *Disability and Rehabilitation.* 2015;37(8):721-727.

38. DiSotto-Monastero M, Chen X, Fisch S, Donaghy S, Gomez M. Efficacy of 7 days per week inpatient admissions and rehabilitation therapy. *Archives of Physical Medicine and Rehabilitation.* 2012;93(12):2165-2169.

39. Haas R, O'Brien L, Bowles K, Haines T. Effectiveness of a pragmatic weekend physical therapy service on short-term outcomes following hip and knee arthroplasty. *Manuscript submitted for publication.* 2017.

40. Needle J, Petchey R, Benson J, Scriven A, Lawrenson J, Hilari K. The role of allied health professionals in health promotion. 2011.

41. Peiris CL, Taylor NF, Shields N. Extra Physical Therapy Reduces Patient Length of Stay and Improves Functional Outcomes and Quality of Life in People With Acute or Subacute Conditions: A Systematic Review. *Archives of Physical Medicine and Rehabilitation.* 2012;92(9):1490-1500.

42. Haas R, Sarkies M, Bowles K-A, O'Brien L, Haines T. Early commencement of physical therapy in the acute phase following elective lower limb arthroplasty produces favorable outcomes: a systematic review and meta-analysis examining allied health service models. *Osteoarthritis and Cartilage.* 2016.

43. Kimmel LA, Liew SM, Sayer JM, Holland AE. HIP4Hips (High Intensity Physiotherapy for Hip fractures in the acute hospital setting): a randomised controlled trial. *The Medical Journal of Australia.* 2016;205(2):73-78.

44. Calthorpe S, Barber EA, Holland AE, Kimmel L, Webb MJ, Hodgson C, Gruen RL. An intensive physiotherapy program improves mobility for trauma patients. *Journal of Trauma and Acute Care Surgery.* 2014;76(1):101-106.

45. Mills E, Hume V, Stiller K. Increased allied health services to general and acute medical units decreases length of stay: comparison with a historical cohort. *Australian Health Review.*

FIGURES

Figure 1: PRISMA Flow Diagram

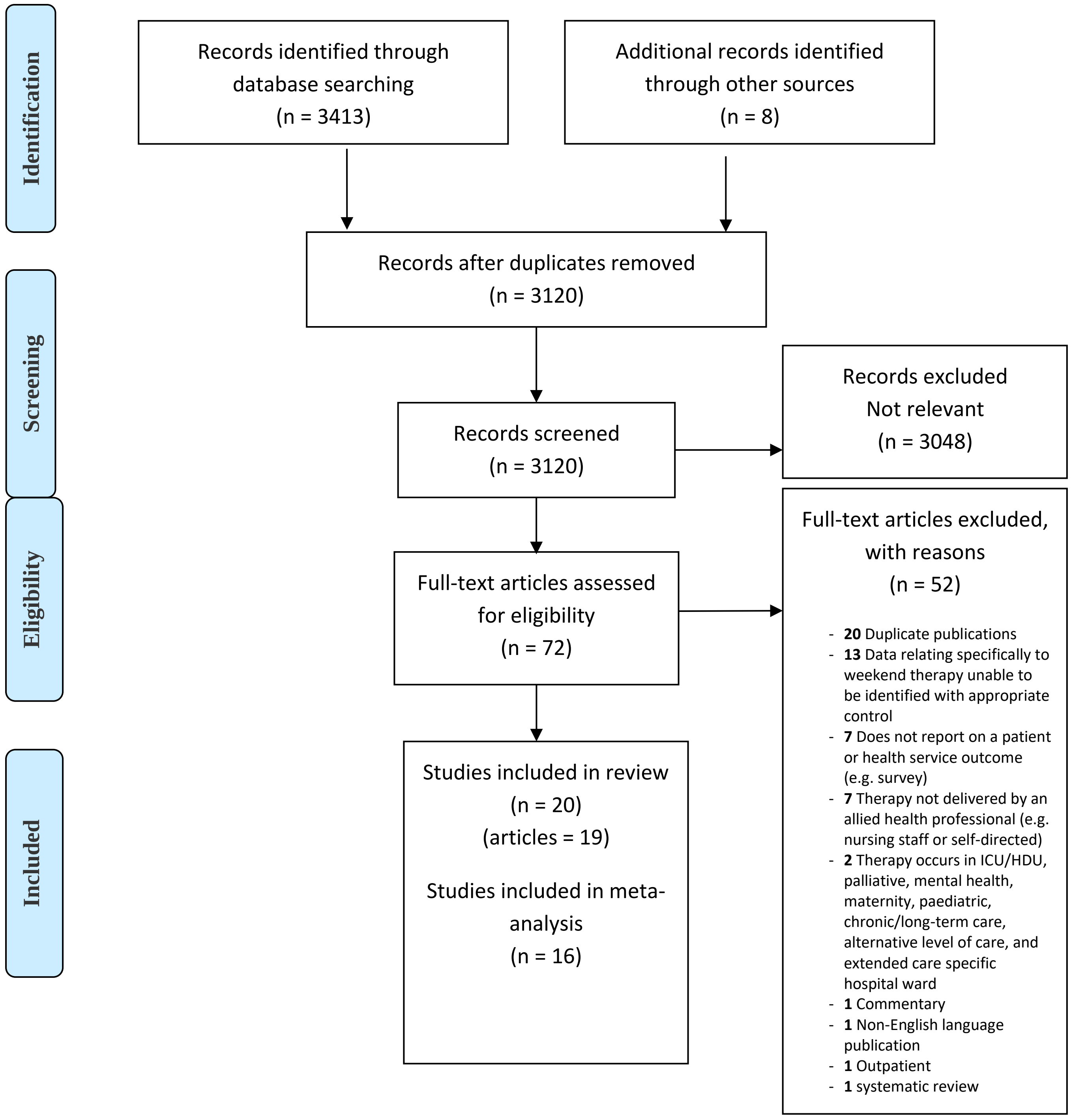

Figure 2. Forest plot for effect of weekend allied health on hospital length of stay: (a) acute RCT; (b) acute non-RCT; (c) sub-acute RCT; (d) sub-acute non- RCT

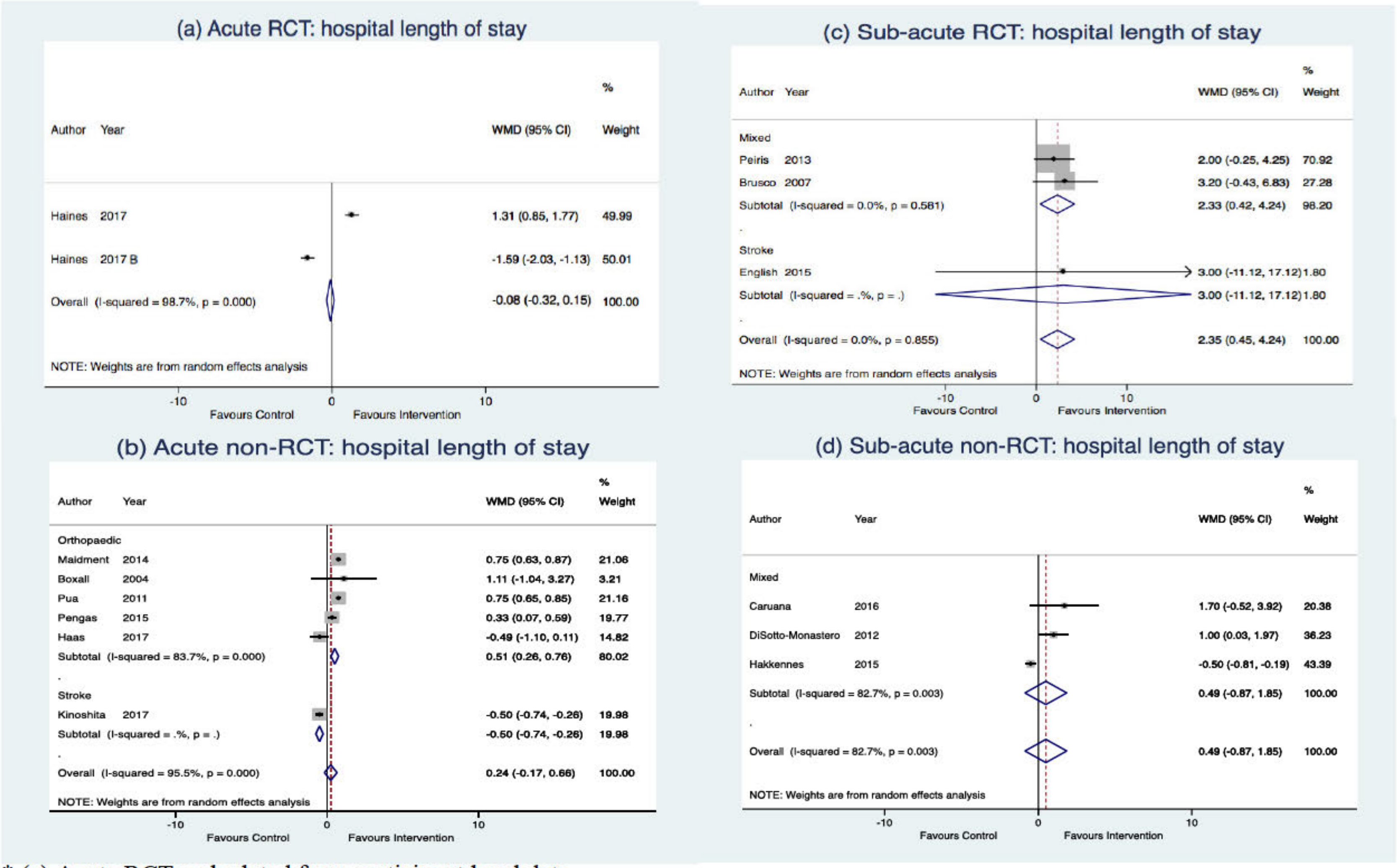

* (a) Acute RCT: calculated from participant level data

Figure 3. Forest plot of acute RCT's for effect of weekend allied health on proportion of patients whose hospital length of stay was longer then their expected length of stay

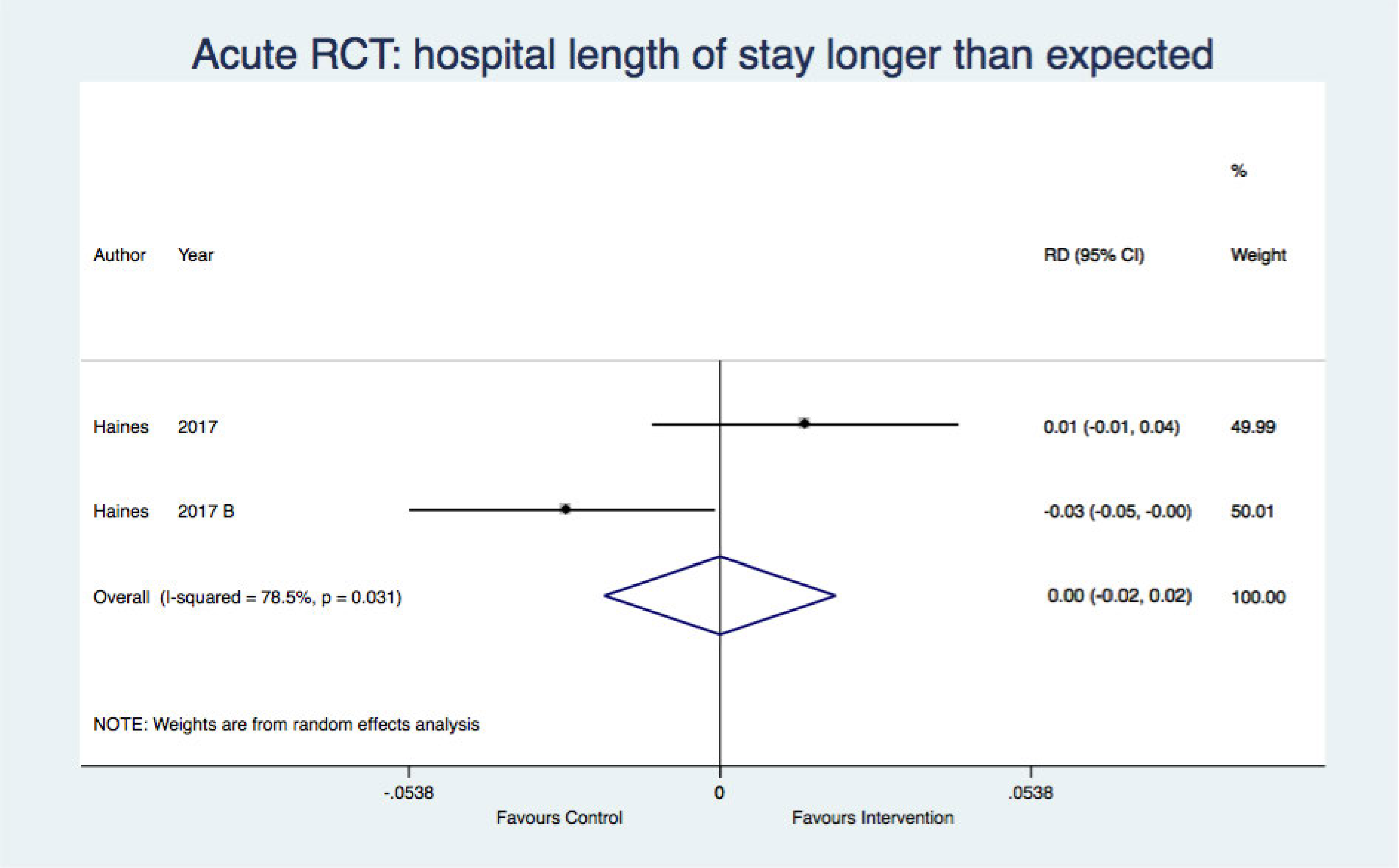

* Acute RCT: calculated from cluster level data

Figure 4. Forest plot for effect of weekend allied health on hospital readmission

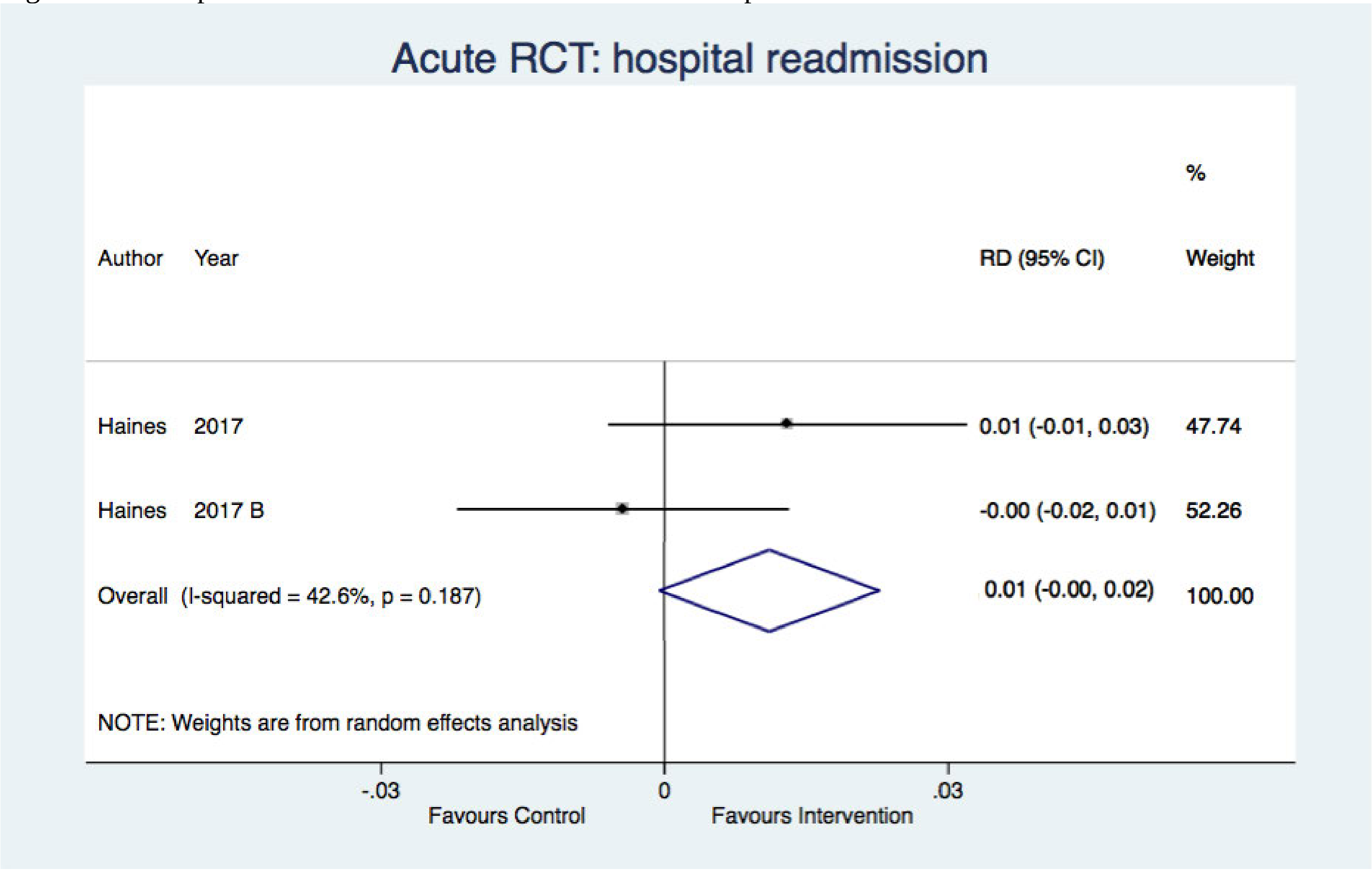

* Acute RCT: calculated from cluster level data

Figure 5. Forest plot for effect of weekend allied health on adverse events: (a) acute RCT; (b) acute non-RCT; (c) sub-acute RCT

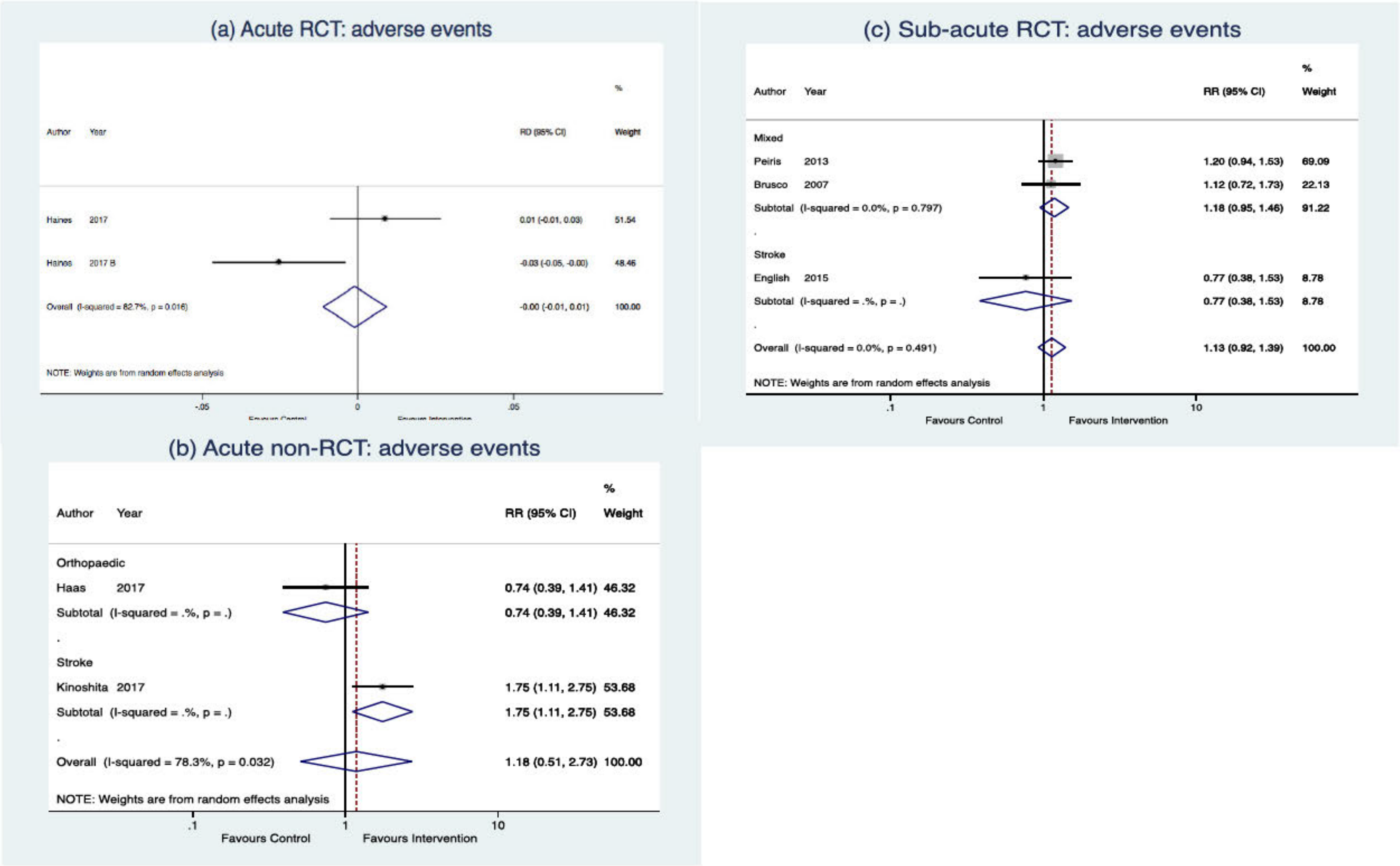

* Acute RCT: calculated from cluster level data

Figure 6. Forest plot for effect of weekend allied health on discharge destination: (a) acute RCT; (b) acute non-RCT; (d) sub-acute non-RCT.

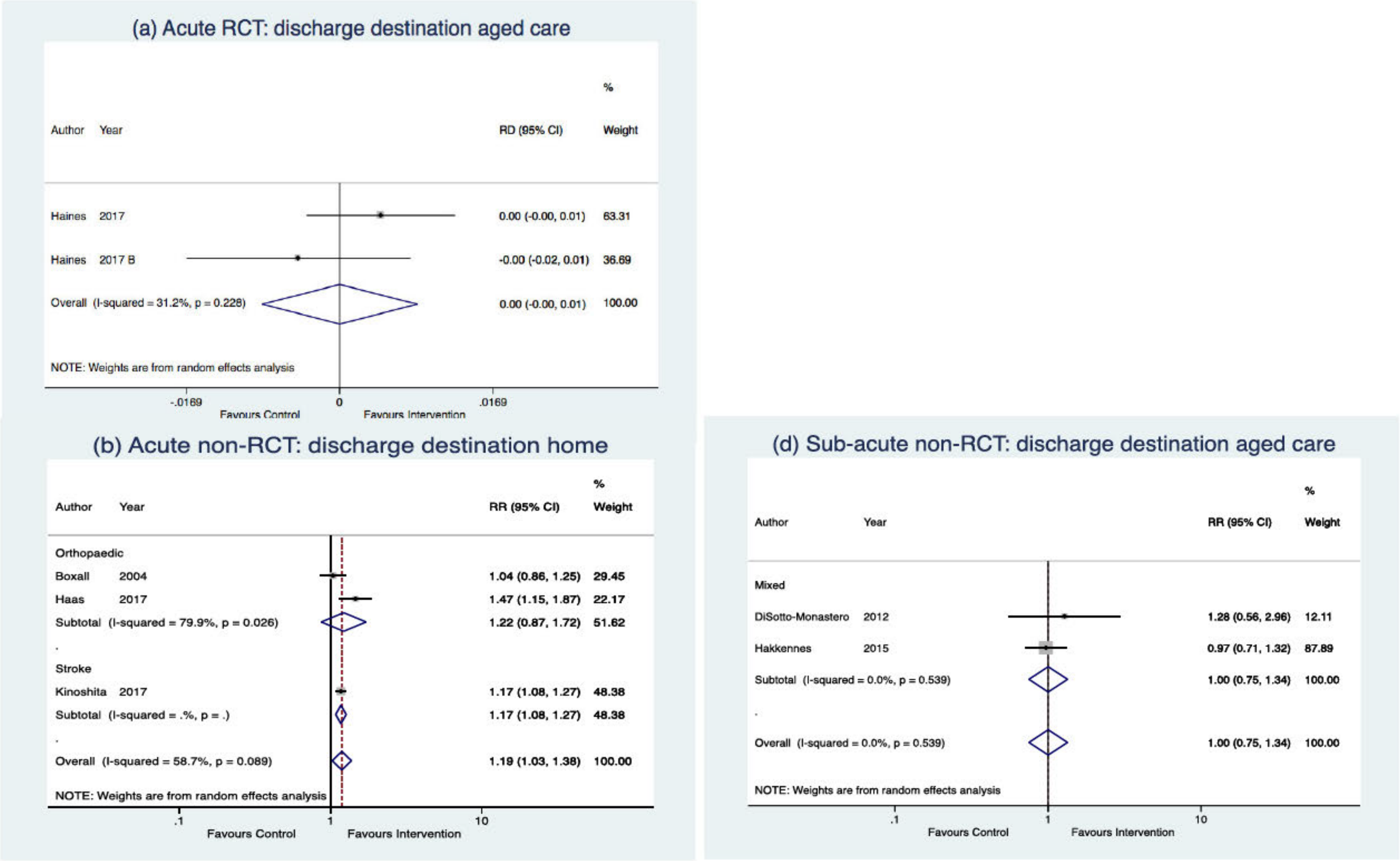

* Acute RCT: calculated from cluster level data

Figure 7. Forest plot for effect of weekend allied health on ADL function: (b) acute non-RCT; (c) sub-acute RCT; (d) sub-acute non-RCT

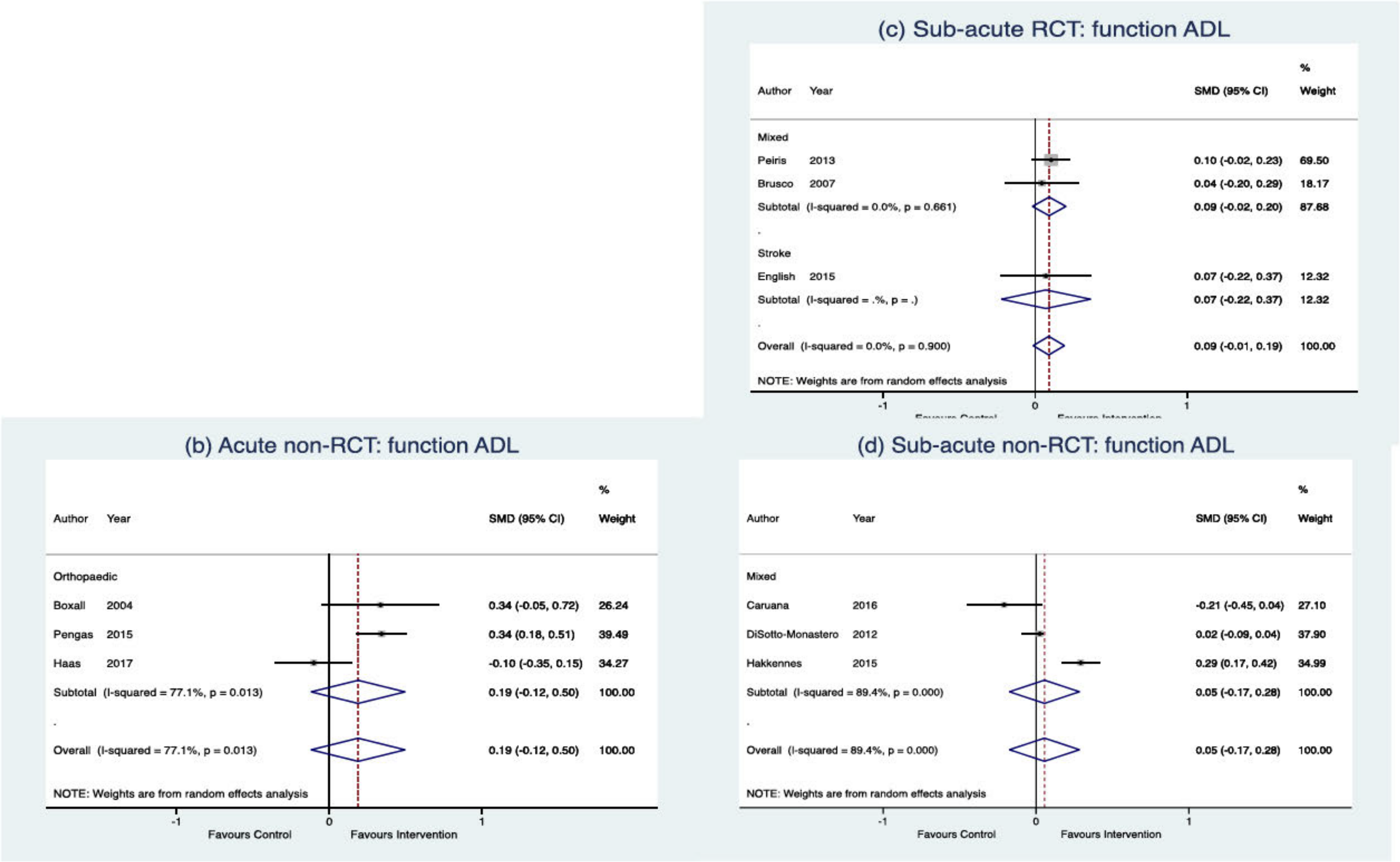

Figure 8. Forest plot for the effect of weekend allied health on quality of life

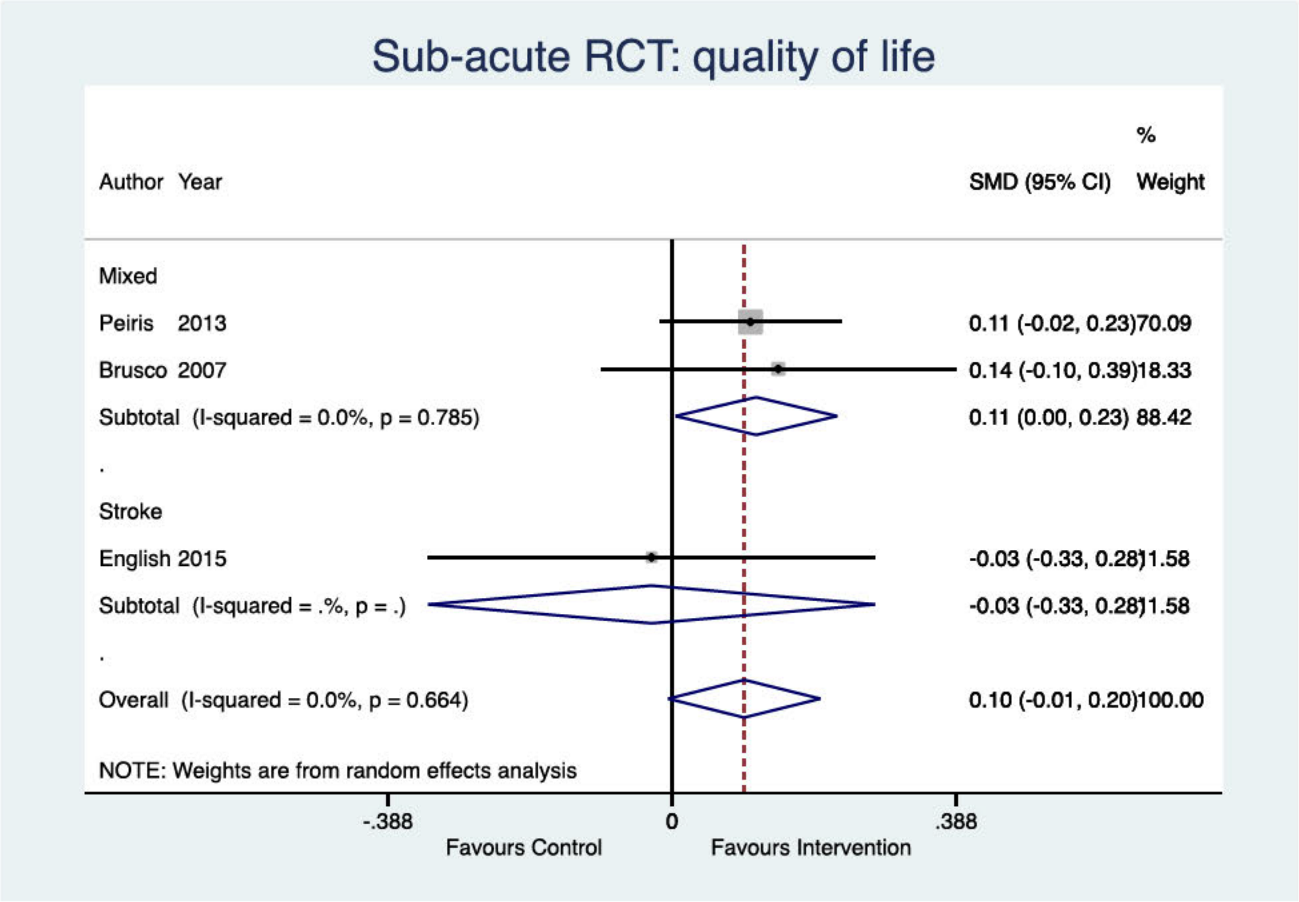

Figure 9. Forest plot for the effect of weekend allied health on cost

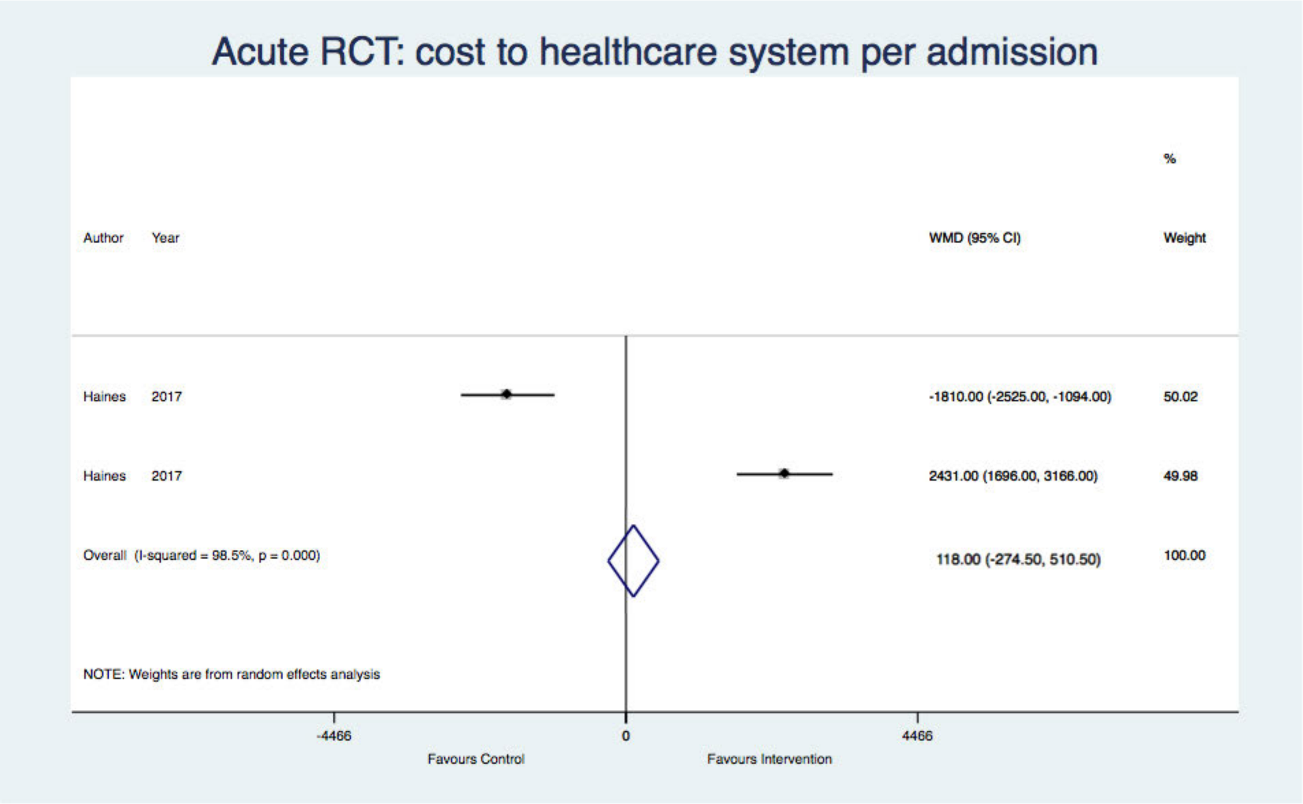

* Acute RCT: calculated from participant level data

Appendix 1: NHMRC Evidence Hierarchy: designations of 'levels of evidence' according to type of research question (including explanatory notes)

Level	Intervention 1	Diagnostic accuracy 2	Prognosis	Aetiology 3	Screening Intervention
I 4	A systematic review of level II studies	A systematic review of level II studies	A systematic review of level II studies	A systematic review of level II studies	A systematic review of level II studies
II	A randomised controlled trial	A study of test accuracy with: an independent, blinded comparison with a valid reference standard, among consecutive persons with a defined clinical presentation	A prospective cohort study	A prospective cohort study	A randomised controlled trial
III-1	A pseudo-randomised controlled trial (i.e. alternate allocation or some other method)	A study of test accuracy with: an independent, blinded comparison with a valid reference standard, among non-consecutive persons with a defined clinical presentation	All or none	All or none	A pseudo-randomised controlled trial (i.e. alternate allocation or some other method)
III-2	A comparative study with concurrent controls:<br>▪ Non-randomised, experimental trial<br>▪ Cohort study<br>▪ Case-control study<br>▪ Interrupted time series with a control group	A comparison with reference standard that does not meet the criteria required for Level II and III-1 evidence	Analysis of prognostic factors amongst persons in a single arm of a randomised controlled trial	A retrospective cohort study	A comparative study with concurrent controls:<br>▪ Non-randomised, experimental trial<br>▪ Cohort study<br>▪ Case-control study
III-3	A comparative study without concurrent controls:<br>▪ Historical control study<br>▪ Two or more single arm study<br>▪ Interrupted time series without a parallel control group	Diagnostic case-control study	A retrospective cohort study	A case-control study	A comparative study without concurrent controls:<br>▪ Historical control study

					▪ Two or more single arm study
IV	Case series with either post-test or pre-test/post-test outcomes	Study of diagnostic yield (no reference standard)	Case series, or cohort study of persons at different stages of disease	A cross-sectional study or case series	Case series

Appendix 2: Search Term Summary

Population	AND	Intervention 1	AND	Intervention 2
Hospital		Allied health		Weekend
**OR**		**OR**		**OR**
Acute		Physiotherap*		Saturday
**OR**		**OR**		**OR**
Subacute		Physical therap*		Sunday
**OR**		**OR**		**OR**
Ward		Occupational therap*		Out of hour
**OR**		**OR**		**OR**
Inpatient		Speech patholog*		After hour
		**OR**		**OR**
		Speech therap*		On call
		**OR**		**OR**
		Language patholog*		Six day
		**OR**		**OR**
		Language therap*		6 day
		**OR**		**OR**
		Nutrition		Seven day
		**OR**		**OR**
		Dietetic*		7 day
		**OR**		
		Dietitian		
		**OR**		
		Dietician		
		**OR**		
		Social work		
		**OR**		
		Art therapy		
		**OR**		
		Chiropractic		
		**OR**		
		Exercise physiology		
		**OR**		

	Music therapy
	**OR**
	Oral Health
	**OR**
	Osteopathy
	**OR**
	Podiatry
	**OR**
	Psychology
	**OR**
	Allied health assistant

Appendix 3: Inclusion and Exclusion Criteria

Inclusion	Exclusion
Allied health service delivered by an allied health professional or allied health assistant	Allied health service not delivered by an allied health professional or allied health assistant (e.g. nursing staff or self-directed)
Allied health service occurs on a general acute medical or surgical ward, Allied health service occurs on a sub-acute rehabilitation ward	Allied health service does not occur in an inpatient setting
Allied health service occurs during the weekend	Data relating specifically to weekend allied health services unable to be identified with appropriate control
Effect of allied health on patient or health service outcome is measured	Does not report on a patient or health service outcome (e.g. survey)
	Allied health service occurs in ICU/HDU, palliative, maternity, paediatric, chronic/long-term care, alternate level of care, and extended care specific hospital ward
	Duplicate publication
	Non-English language publication
	Systematic review
	Protocol
	Outpatient
	Commentary

Appendix 4: Detailed information about included studies

First author and year	Study design	Country	Setting	Population	Control	Intervention	Outcome
Haines et al., 2017 [3]	Randomised controlled trial	Australia	Acute	Mixed general medical and surgical (wards=12; n=14834)<br>C n = 6796; Mean age (SD) 60.8 (20.2)<br>I n = 8038; Mean age (SD) 59.5 (20.7)<br><br>Hospital 1, 6 inpatient wards: orthopaedic surgery, stroke, thoracic/vascular/general surgery & medical, general medicine, head/neck/plastics, and surgical<br><br>Hospital 2, 6 inpatient wards: medical (2 wards), infectious diseases/respiratory, plastics/ENT/head/neck surgery, general surgery/colorectal/breast/endocrine/urology, and general surgery/ vascular/thoracic/upper gastrointestinal	No allied health services on weekends	Usual care allied health services on weekends<br><br>Hospital 1<br>*Saturday (hours per hospital):*<br>PT (8), OT (3), SP (3.5), DT (2), SW (1), AHA (4)<br>*Sunday (hours per hospital):*<br>PT (11), OT (3), SP (3), DT (2), SW (1), AHA (4)<br><br>Hospital 2<br>*Saturday (hours per hospital):*<br>PT (3.25), OT (3.5)<br>*Sunday (hours per hospital):*<br>PT (3.25)	Primary: (1) Hospital length of stay; (2) Length of stay longer than expected length of stay; (3) Hospital readmission; (4) Adverse events<br><br>Secondary: (1) Compliments and complaints; (2) Discharge destination; (3) Occasions of Allied health service; (4) Cost of hospital admission; (5) Clinical exceptions; (6) Staff absenteeism
Haines et al., 2017 B [3]	Randomised controlled trial	Australia	Acute	Mixed general medical and surgical (wards=12; n=12674)<br>C n = 6869; Mean age (SD) 59.7 (20.6)<br>I n = 5805; Mean age (SD) 59.8 (20.3)<br><br>Hospital 1, 6 inpatient wards: orthopaedic surgery, stroke, thoracic/vascular/general surgery & medical, general medicine, head/neck/plastics, and surgical	No allied health services on weekends	Newly developed stakeholder-driven weekend allied health service<br><br>Hospital 1<br>*Friday (hours per hospital):*<br>PT (8), OT (4), AHA (4)<br>*Saturday (hours per hospital):*<br>PT (3.5), OT (2), SP (3.5), DT (1), SW (1)<br>*Sunday (hours per hospital):*<br>PT (7), OT (2), SP (3), DT (1), SW (1), AHA (4)	Primary:<br>(1) Hospital length of stay; (2) Length of stay longer than expected length of stay; (3) Hospital readmission; (4) Adverse events<br><br>Secondary: (1) Compliments and complaints; (2) Discharge destination; (3) Occasions

				Hospital 2, 5 inpatient wards: medical (2 wards), infectious diseases/respiratory, plastics/ENT/head/neck surgery, general surgery/colorectal/breast/endocrine/urology, and general surgery/ vascular/thoracic/upper gastrointestinal		*Monday (hours per hospital):* PT (4), OT (4)<br><br>Hospital 2<br>*Saturday (hours per hospital):* ICU PT (1), IRS (4), SP (2)<br>*Sunday (hours per hospital)* ICU PT (1)	of Allied health service; (4) Cost of hospital admission; (5) Clinical exceptions; (6) Staff absenteeism
Brusco et al., 2007 [4]	Randomised controlled trial	Australia	Sub-acute	Mixed rehabilitation (wards=2; n=262)<br>C n = 132; Mean age (SD) 77 (13)<br>I n = 130; Mean age (SD) 77 (13)	5-day weekday physiotherapy (daily hours per patient)<br><br>PT (1)	Additional Saturday physiotherapy (daily hours per patient)<br><br>PT (1)	Primary: (1) Hospital length of stay; (2) Physiotherapy length of stay<br><br>Secondary: (1) EuroQol; (2) Functional Independence Measure; (3) Functional reach; (4) 10 Meter Walk Test; (5) Timed Up and Go Test; (6) Motor Assessment Scale; (7) Knee and Hip Range Of Motion; (8) Discharge destination; (9) Adverse events; (10) Follow-up therapy
Brusco et al., 2014 [25]	Randomised controlled trial	Australia	Sub-acute	Mixed rehabilitation (hospitals=2; beds=90; n=996)<br>C n = 500; Mean age (SD) 74 (13)<br>I n = 496; Mean age (SD) 75 (13)	5-day weekday physiotherapy and occupational therapy (daily hours per patient)<br><br>PT (1)<br>OT (1)	Additional Saturday physiotherapy and occupational therapy (daily hours per patient)<br><br>PT (1)<br>OT (1)	(1) Health service and therapy utilisation (30-day follow-up); (2) Cost of inpatient rehabilitation (30-day follow-up)
Brusco et al., 2014B [24]	Randomised controlled trial	Australia	Sub-acute	Mixed rehabilitation (hospitals=2; beds=90; n=137)<br>C n = 63; Mean age (SD) 60.7 (13.3)<br>I n = 74; Mean age (SD) 62.6 (11.9)	5-day weekday physiotherapy and occupational therapy (daily hours per patient)<br><br>PT (1)<br>OT (1)	Additional Saturday physiotherapy and occupational therapy (daily hours per patient)<br><br>PT (1)<br>OT (1)	Primary: (1) Return to work; (2) Average hours worked; (3) Paid income<br><br>Secondary: (1) Functional Independence Measure; (2)

							EuroQol-5D; (3) Hospital length of stay
Brusco et al., 2015 [26]	Randomised controlled trial	Australia	Sub-acute	Mixed rehabilitation (hospitals=2; beds=90; n=996)<br>C n = 500; Mean age (SD) 74 (13)<br>I n = 496; Mean age (SD) 75 (13)	5-day weekday physiotherapy and occupational therapy (daily hours per patient)<br><br>PT (1)<br>OT (2)	Additional Saturday physiotherapy and occupational therapy (daily hours per patient)<br><br>PT (1)<br>OT (1)	(1) Health service and therapy utilisation (12-month follow-up); (2) Cost of inpatient rehabilitation (12-month follow-up)
English et al., 2014 [28]	Randomised controlled trial	Australia	Sub-acute	Stroke rehabilitation (centres=5; n=21)<br>C n = 10; Mean age (SD)<br>I n = 11; Mean age (SD)	Usual care physiotherapy (recruitment sites)<br><br>Individual therapy 5-days per week (3/5 sites)<br>Individual or group therapy between 1-4 days per week (2/5 sites)<br>Weekend therapy for some patients (2/5 sites)	7-day physiotherapy (daily duration per patient)<br><br>PT (matched to preceding week)	(1) Therapy duration; (2) Reasons for shortened therapy; (3) Reasons for non-attendance; (4) Activity across day; (5) Activity during therapy; (6) Activity outside therapy; (7) Activity location; (8) Activity with people present
English et al., 2015 [5]	Randomised controlled trial	Australia	Sub-acute	Stroke rehabilitation (centres=5; n=190)<br>C n = 94; Mean age (SD) 68.2 (13.5)<br>I n = 96; Mean age (SD) 71.9 (12.0)	Usual care physiotherapy (recruitment sites)<br><br>Individual therapy 5-days per week (3/5 sites)<br>Individual or group therapy between 1-4 days per week (2/5 sites)<br>Weekend therapy for some patients (2/5 sites)	7-day physiotherapy (daily duration per patient)<br><br>PT (matched to preceding week)	Primary: (1) Six-minute was test<br><br>Secondary: (1) Walking speed; (2) functional ambulation category; (3) Functional Independence Measure; (4) Wold Motor Function test; (5) Stroke Impact Scale physical subscale; (6) Hospital length of stay; (7) Assessment of Quality of Life; (8) Adverse events; (9) Resource utilisation
Peiris et al., 2012 [27]	Randomised controlled trial	Australia	Sub-acute	Mixed rehabilitation (hospitals=2; beds=90; n=105)<br>C n = 54; Mean age (SD) 73 (13)<br>I n = 51; Mean age (SD) 75 (12)	5-day weekday physiotherapy and occupational therapy (daily hours per patient)<br><br>PT (1)<br>OT (1)	Additional Saturday physiotherapy and occupational therapy (daily hours per patient)<br><br>PT (1)<br>OT (1)	Primary: (1) Steps per day; (2) Time spent upright<br><br>Secondary: (1) Time spent inactive; (2) Activity completed in therapy

Peiris et al., 2013 [6]	Randomised controlled trial	Australia	Sub-acute	Mixed rehabilitation (hospitals=2; beds=90; n=996)<br>C n = 500; Mean age (SD) 74 (13)<br>I n = 496; Mean age (SD) 75 (13)	5-day weekday physiotherapy and occupational therapy (daily hours per patient)<br><br>PT (1)<br>OT (1)	Additional Saturday physiotherapy and occupational therapy (daily hours per patient)<br><br>PT (1)<br>OT (1)	Primary: (1) Functional Independence Measure; (2) EuroQol-5D; (3) Hospital length of stay<br><br>Secondary: (1) Personal Care-Participation Assessment and Resource Tool; (2) 10 Metre Walk Test; (3) Timed Up and Go Test; (4) Modified Motor Assessment Scale; (5) Discharge destination; (6) Follow-up therapy; (7) Adverse events
Boxall et al., 2004 [30]	Cohort	Australia	Acute	Orthopaedic (wards=1; n=240)<br>C n = 120; Mean age (SD) 67.97<br>I n = 120; Mean age (SD) 68.03	5-day weekday physiotherapy	7-day physiotherapy service	(1) Hospital length of stay; (2) Days to independent transfers; (3) Days to independent mobility; (4) Reasons for delayed discharge; (5) Discharge destination; (6) Pre-admission clinic attendance
David et al., 2003 [34]	Cohort	England	Acute	Rheumatology (wards=1; beds=28; n=361)<br>C n = 146; Mean age (SD)<br>I n = 215; Mean age (SD)	5-day weekday physiotherapy (total EFT)<br><br>PT (2)<br>AHA (0.3)	Additional weekend physiotherapy (total EFT)<br><br>PT (0.2)<br>AHA (0.4)	(1) Number of Rheumatology admissions; (2) Hospital length of stay; (3) Day of admission and discharge; (4) Staff cost and utilisation
Haas et al., 2017 [39]	Cohort	Australia	Acute	Orthopaedic (wards=2; beds=48; n=276)<br>C n = 146; Mean age (SD) 68.58 (10.29)<br>I n = 130; Mean age (SD) 67.77 (10.62)	5-day weekday allied health service: physiotherapy, occupational therapy, speech pathology, dietetics, and social work	Saturday and Sunday allied health: physiotherapy, occupational therapy, speech pathology, dietetics, and social work	(1) Hospital length of stay; (2) Adverse events; (3) Discharge destination; (4) Time till first post-operative transfer; (5) Staff profession assisting first post-operative transfer; (6) Physiotherapy session rate; (7) Time till first post-op physiotherapy session; (8) Modified Barthel Index; (9) DE-Mortin Mobility Index;

							(10) EuroQol-5D utility; (11) EuroQol-5D VAS; (12) Patient satisfaction; (13) Pain; (14) Opioid use
Kinoshita et al., 2017 [35]	Cohort	Japan	Acute	Stroke (hospitals=14; n=3072)<br>C n = 1997; Mean age (SD) 72.67 (12.61) *<br>I n = 1075; Mean age (SD) 74.33 (11.88) *	5-day or 6-day physiotherapy and occupational therapy	7-day physiotherapy and occupational therapy	(1) Modified Rankin Scale; (2) Hospital length of stay; (3) Adverse events; (4) Discharge destination
Maidment et al., 2014 [29]	Cohort	Australia	Acute	Orthopaedic (wards=1; n=145)<br>C n = 59; Mean age (SD) 71.67 (8.36) *<br>I n = 86; Mean age (SD) 69.27 (6.94) *	5-day physiotherapy service	7-day physiotherapy service	(1) Physiotherapy length of stay; (2) Hospital length of stay; (3) Number of Physiotherapy sessions; (4) Reasons for delayed discharge
Pengas et al., 2015 [32]	Cohort	Scotland	Acute	Orthopaedic (n=792)<br>C n = 600; Mean age (SD)<br>I n = 192; Mean age (SD)	5-day weekday physiotherapy	Additional Saturday and Sunday physiotherapy provided by an allied health assistant (hours)<br><br>AHA (3)	(1) Days to mobilising with 2-sticks; (2) Hospital length of stay; (3) Time to achieve 90° knee flexion; (4) Range of Motion
Pua et al., 2011 [31]	Cohort	Singapore	Acute	Orthopaedic (n=155)<br>C n = 82; Mean age (SD) 65.4 (7.3)<br>I n = 73; Mean age (SD) 66.3 (8.0)	6-day Monday to Saturday physiotherapy (daily OOS)<br><br>PT (1)	Additional Sunday physiotherapy	(1) Hospital length of stay; (2) Passive Range of Motion- knee; (3) Straight Leg Raise ; (4) Independent mobility
Caruana et al., 2016 [36]	Cohort	Australia	Sub-acute	Mixed rehabilitation (wards=1; beds=40; n=270)<br>C n = 108; Mean age (SD) 75.5 (4.3)<br>I n = 162; Mean age (SD) 78.0 (12.3)	5-day Monday to Friday physiotherapy, occupational therapy, speech pathology, and dietetics	Additional Saturday physiotherapy (daily hours)<br><br>PT and AIN (4)	Primary: (1) Hospital length of stay<br><br>Secondary: (1) Functional Independence Measure; (2) Timed Up and Go Test; (3) 10 Metre Walk Test; (4) Functional reach; (5) Step test; (6) Feet Together Eyes Closed; (7) Balance Outcome Measure for Elder Rehabilitation
DiSotto-Monaster	Cross-sectional	Canada	Sub-acute	Mixed rehabilitation (n=3500)<br>C n = 1692; Mean age (SD) 71.9 (13)	5-day physiotherapy and occupational therapy	7-day physiotherapy and occupational therapy	(1) Functional Independence Measure; (2) Number of admissions and

o et al., 2012 [38]				I n = 1808; Mean age (SD) 72.2 (13.6)			discharges; (3) Hospital length of stay Rehabilitation workload
Hakkennes et al., 2015 [37]	Cohort	Australia	Sub-acute	Mixed rehabilitation (hospital=1; beds=100; n=976)<br>C n = 499; Mean age (SD) 78.33 (10.41) *<br>I n = 477; Mean age (SD) 77.67 (11.89) *	5-day weekday therapy	Additional Saturday physiotherapy, occupational therapy, and social work (daily hours per hospital)<br><br>PT (20)<br>OT (16)<br>SW (8)<br>AHA (6)	(1)Functional Independence Measure; (2) Hospital length of stay; (3) Number of admissions on Saturday

*=converted from median (range/inter-quartile range) to mean (SD); hours=hours per day; OOS=occasions of service; PT=physiotherapy, OT=occupational therapy, SP=speech pathology, DT=dietetics, SW=social work, AHA=allied health assistant, AIN=assistant in nursing; IRS=Immediate Response Service; ICU=intensive care unit; and C=control, I=intervention, n=population, SD=standard deviation
