# Supplementary material for: Effectiveness of knowledge brokering and recommendation dissemination for influencing healthcare resource allocation decisions: A cluster randomised controlled implementation trial
Source: PLoS Med. 2021 Oct 22;18(10):e1003833. doi: 10.1371/journal.pmed.1003833 (PMC8570499; doi:10.1371/journal.pmed.1003833)
Supplement: Attachment — Submitted filename: Response to comments 140921.docx [file pmed.1003833.s009.docx]

**Response to comments**

**Effectiveness of knowledge brokering and recommendation dissemination for influencing healthcare resource allocation decisions: A cluster randomised controlled implementation trial (PMEDICINE-D-21-01199_R4)**

The authorship team would like to extend our appreciation to the editor and peer reviewers for considering our manuscript revision. We have responded to each comment and modified the manuscript accordingly.

**Requests from Editors:**

Thank you for responding to editorial requests. Before we proceed, please address the following comments:

Editorial comment 1) Please highlight in the conclusion (both abstract and main text), that you did not observe a difference imparted by the implementation strategies, owing to lack of power. For example, you could state “owing to lack of power, our study was unable to find whether…”

Author response 1) We thank the editor for their suggestion and have modified the conclusion (in the abstract and main text) to begin with the phrase *“Owing to a lack of power at the cluster-level, this trial was unable to…”*

Editorial comment 2) In the author summary, please revise line 110... should this be “are” instead of “are represent”?

Author response 2) Thank you for identifying this error. We have removed the word *“represent”* from the last dot point of the author summary.

Editorial comment 3) In the abstract, last sentence of “Methods and Findings” please clearly highlight the limitations of the study. The statement, “Limitations of this study include…” can be useful.

Author response 3) We have added the statement *“Limitations of this study include…”* and listed underpowering at the cluster-level and unexpected high-levels of recommendation alignment in the knowledge broker group at baseline.

Editorial comment 4) References #55, #57: please include access dates for the weblinks, e.g., Accessed July 15, 2021.

Author response 4) Thank you, we have added access dates to the two references.

**Comments from Reviewers:**

Reviewer #2, comment 1: The authors have addressed all my concerns professionally. I am satisfied with the response and revision. No further issues needing attention. Thanks.

Author response: We thank the reviewer for their detailed and insightful statistical review. The manuscript has been greatly strengthened by the reviewer’s suggestions.

Reviewer #4, comment 1: I'd like to thank the authors for their efforts to address the comments.

I think that the researchers could use more suitable implementation theoretical frameworks to guide the design of the study, as well as more relevant and sensitive implementation outcomes to capture the impact of individual, team-level, and organizational factors that could determine the success/failure of the intervention. Despite this, I do not object the publication of this study, given its rigorous design and potentially important implications for further research on knowledge broker interventions.

However, I re-emphasize that, it should be clearly stated in the study limitations that the observed lack of evidence to support knowledge broker intervention could simply be due the fact that the intervention was not successfully implemented (for which insufficient evidence was provided). I recommend that the authors suggest potential solutions for more theoretically-informed assessment of the implementation of KB interventions.

Author response: We appreciate the implementation science focused review provided by Reviewer 4, whose insightful comments have led to the strengthening of this manuscript. The suggestion to use different theories, models or frameworks to structure future evaluations is acknowledged, with many thanks. A broader range of implementation outcomes will be reported in other planned publications. We thank the reviewer for their recommendation to accept our manuscript for publication.

We agree that the operationalisation of the knowledge broker strategy could have influenced its success. The discussion provides an extensive exploration of limitations pertaining to the intervention, including: 1) the external and centralised design, 2) the online delivery, 3) the limited frequency and duration, and 4) the lack of hospital manager engagement. The evaluation was structured using: 1) CONSORT Extension for Cluster Trials checklist; 2) Standards for Reporting Implementation Studies statement (StaRI); 3) Template for Intervention Description and Replication (TIDieR) guidelines; 4) Proctor’s framework for the specification of implementation strategies; 5) Proctor’s implementation outcomes taxonomy; 6) Donabedian model for linking structure, process, and outcome. Furthermore, the knowledge broker strategy was designed to address implementation determinants (barriers and enablers) from a recent systematic review and the knowledge broker prompting questions were informed by the COM-B (Capability, Opportunity, Motivation, and Behaviour) model. Based on the above, we feel the assessment of the knowledge broker and evidence dissemination strategies were adequately informed by relevant theories, models and frameworks.
