# Supplementary material for: Effectiveness of knowledge brokering and recommendation dissemination for influencing healthcare resource allocation decisions: A cluster randomised controlled implementation trial
Source: PLoS Med. 2021 Oct 22;18(10):e1003833. doi: 10.1371/journal.pmed.1003833 (PMC8570499; doi:10.1371/journal.pmed.1003833)
Supplement: Attachment — Submitted filename: Response to reviewer comments 02062021.docx [file pmed.1003833.s007.docx]

**Response to reviewer comments**

**Effectiveness of knowledge brokering and recommendation dissemination for influencing healthcare resource allocation decisions: A cluster randomised controlled implementation trial (PMEDICINE-D-21-01199R2)**

The authorship team would like to extend our appreciation to the editor and reviewers for providing peer review of our manuscript. We have responded to each reviewer comment and made modifications where required. Page and line numbers in each response refer to the “tracked” version of the manuscript.

**Requests from the editors**

**Editor comment 1**: Please revise your title to indicate that this is an implementation science study. Your title must be nondeclarative and not a question. It should begin with main concept if possible. For example, please place the study design ("A cluster randomized controlled implementation trial,") in the subtitle (i.e., after a colon).

**Author response**: The title has been revised to indicate that it is an implementation science study. Please refer to Page 1, line 1-3 which now reads *“Effectiveness of knowledge brokering and recommendation dissemination for influencing healthcare resource allocation decisions: A cluster randomised controlled implementation trial”*

**Editor comment 2**: Abstract summary - At this stage, we ask that you reformat your non-technical Author Summary. The Author Summary should immediately follow the Abstract in your revised manuscript. This text is subject to editorial change and should be distinct from the scientific abstract. The summary should be accessible to a wide audience that includes both scientists and non-scientists. Please see our author guidelines for more information: https://journals.plos.org/plosmedicine/s/revising-your-manuscript#loc-author-summary.

**Author response**: A non-technical author summary has been added immediately after the abstract in accordance with the author guidelines. Please refer to Page 5, line 90-116.

**Editor comment 3**: Abstract:

1. Please structure your abstract using the PLOS Medicine headings (Background, Methods and Findings, Conclusions).

2. Please combine the Methods and Findings sections into one section, “Methods and findings”. Please ensure that all numbers presented in the abstract are present and identical to numbers presented in the main manuscript text.

3. Please quantify the main results (with p values in addition to 95% CI).

4. Please include a summary of adverse events if these were assessed in the study.

**Author response**: 1) The abstract has been structured using the PLOS Medicine headings; 2) We have combined the Methods and Findings sections accordingly and checked the numbers align with those presented in the main text; 3) The main results have been quantified, including p values and 95% CIs; 4) No adverse events were recorded which is now reflected in the abstract. Please refer to Page 3-4, line 41-87.

**Editor comment 4**: When completing the CONSORT checklist, please use section and paragraph numbers, rather than page numbers.

**Author response**: Section headings and paragraph numbers have now been inserted, in addition to page numbers. Please refer to S2 Text.

**Editor comment 5**: In addition to the CONSORT checklist please ensure that your implementation research is reported according to Standards for Reporting Implementation Studies statement (STARI). The STARI guidelines can be found here: https://www.equator-network.org/reporting-guidelines/stari-statement/

**Author response**: Standards for Reporting Implementation Studies statement (StaRI) checklist has been completed in S3 Text.

**Editor comment 6**: Please clearly specify and report your implementation strategies. Consider using the guidelines published by Proctor et al. to improve your reporting: Proctor EK, Powell BJ, McMillen JC: Implementation strategies: recommendations for specifying and reporting. Implement Sci 2013, 8:139

**Author response**: Further specification of the implementation strategies has been provided according to the recommendations by Proctor et al (2013) within the Interventions section. We have added Table 1 for improved clarity (page 12-14, line 246-247).

**Editor comment 7**: To improve the clarity and definition of your primary implementation outcomes, please use standard implementation science terminology such as adoption, fidelity, penetration, sustainability etc.

**Author response**: We have categorised the primary outcome (practice alignment with recommendations) as addressing three implementation outcomes: adoption of evidence-based practice recommendation, penetration among eligible hospital wards, and fidelity to the recommendation. Please refer to changes in the Abstract, page 3, line 61; and Methods, Outcomes section, page 14, line 250-252.

**Editor comment 8**: Please present p-values along with 95% CI in the tables and main text. Please specify the statistical test used to derive p values

**Author response**: p values and 95% CIs have been added to the main text in addition to the tables, please refer to the Results section, page 21, line 374- 383. We have also added which statistical test was used to for each outcome within the Results section in addition to the Methods, please refer to Results section, page 21, line 374 and 380-381.

**Editor comment 9**: Please use the "Vancouver" style for reference formatting and see our website for other reference guidelines https://journals.plos.org/plosmedicine/s/submission-guidelines#loc-references. Please ensure that weblinks are current and accessible

**Author response**: We have reviewed the referencing throughout and ensured alignment with the recommended “Vancouver” style., please refer to changes in References section, Page 29-33, line 545-772.

**Comments from the reviewers**

**Reviewer 1, comment 1**: The evaluation of knowledge brokering is important to contribute to the knowledge translation literature. As the authors point out there have been relatively few empirical studies conducted to date evaluating the impact of knowledge brokering on the use of evidence-informed practices and/or recommendations. I enjoyed reading this article and believe, with revisions, it will be an important contribution to the literature. However, I have a few concerns that I feel are important to be addressed prior to publication.

**Author response**: We thank the reviewer for their time and efforts providing peer review for our manuscript. Provided below is response to each comment, where we reference corresponding changes made to the main text. The manuscript has been greatly strengthened by the reviewer’s contributions.

**Reviewer 1, Major Concerns**

**Reviewer 1, comment 1**: greater justification for the use of a cluster randomized controlled trial given previous suggestions in the literature to avoid the use of such a design to evaluate knowledge brokering.

**Author response**: We thank the reviewer for identifying where we could improve our explanation for why cluster randomised controlled designs are needed to evaluate knowledge brokering (and other strategies). Evidence of effectiveness is required given the substantial cost and resourcing required to deliver formalised knowledge broker roles: providing funds for activities with unknown levels of effectiveness such as knowledge brokering is difficult to justify, as those same funds can no longer be used for alternative activities that are known to effectively improve health outcomes elsewhere in the health system (please refer to text added to the end of the Introduction section). Randomised controlled trials are the most suitable designs to answer questions of effectiveness in circumstances where study conditions can be feasibly assigned at random, and clustering allowed us to avoid potential contamination across study conditions and capture outcomes at the system level where changes were expected to occur. The authors acknowledge the benefits of different study designs for different research questions and contextual circumstances, such as more exploratory approaches to elucidate the “how, why, where, and when” in relation to knowledge broker strategies. We have inbuilt these concurrent investigations within our study, which are planned for publication elsewhere. Please refer to text added to the Methods, Trial design section, page 10, line 191-193.

**Reviewer 1, comment 2**: More detail needs to be provided about the knowledge brokering intervention as this is very sparse.

**Author response**: In response to the Editor and Reviewer 1’s comment to expand our description of the knowledge broker strategy, we have provided additional text within the Methods, Interventions section, page 12-14, line 229-247 and added Table 1, page 12-14, line 246-247, which specifies and reports the details of both interventions according to recommendations by Proctor et al (2013). Further descriptions of the study conditions can be obtained in our referenced protocol (see S1 Text) and trial registration (see REF 44).

**Reviewer 1, comment 3**: However, my understanding from what is written about the knowledge brokering intervention is that in entailed interactive webinars. While this is certainly one activity that knowledge brokers engage in, if this was the only activity conducted during this study, I would not classify this as knowledge brokering. As the authors point out in the discussion, knowledge brokering involves building relationships with end users and considerable interaction. It does not appear from the description provided that these activities occurred, which might suggest that what was actually evaluated in this study was webinars as a knowledge translation strategy delivered by a knowledge broker.

**Author response**: The reviewer raises a valid concern in relation to the knowledge broker strategy as it was described upon manuscript submission. As indicated by the reviewer, knowledge brokering is an umbrella term for multiple permutations which we believe is better captured by re-phrasing our strategy as a “webinar-based knowledge broker”. Our strategy involved multiple contact points via different modes: 1) initial individualised contact made via email or phone to confirm receipt of the written recommendations, discuss local needs, and discuss a plan over the next 12-months; 2) within 6-months (according to hospital manager availability) a group webinar was arranged; 3) the group webinar was followed up by individualised contact via email or phone (according to hospital manager preference); 4) a final group webinar was arranged; 5) follow up individualised contact thereafter on an “as needs” basis. Contacts made over a 12-month period with dose varying according to levels of participant engagement. Given the cost and resources associated with traditional, in-person models of knowledge brokering (particularly for Australia and other countries with large geographical distances between sparse populations), many knowledge brokering models have shifted to remote delivering via webinar, email and phone. This trend has been further emphasised during the COVID-19 Pandemic. Please refer to the Methods, Interventions section for additional text and Table 1.

**Reviewer 1, comment 4**: Also, previous research has suggested that knowledge brokering likely needs to be implemented for more than 12 months to be effective, so perhaps some more justification can be provided for why a 12 month intervention was implemented in this study.

**Author response**: The duration of our intervention was based on a similar trial evaluating a knowledge broker role by Dobbins et al (2009), which also aligned with our hypothesis that a 12-month intervention duration would prove sufficient time for hospital managers to develop and implement a business case for change. Please refer to additional text in the Methods, Interventions section, page 12, line 242-245. Commentators have speculated that knowledge broker strategies of longer duration and higher intensity might prove more beneficial, however empirical evidence to support this claim is insufficient. More recent trials have explored shorter durations, such as Minian et al (2021) who used a six-month follow up.

**Reviewer 1, comment 5**: A significant challenge for this study is the extent to which it is under powered to detect even a large difference. I have two issues here. The sample size calculation is based on a very large effect (50% alignment with the allied health allocation recommendation). Justification from previous knowledge brokering studies for such a large effect should be provided. However, I am not certain that there is evidence to suggest that knowledge brokering evaluations in the past has found such large effects, in which case, a much more conservative effect size should have been used in the sample size calculation. The challenge this creates is that the required sample size would be much larger than it already is, which makes this study even more under-powered.

**Author response**: Indeed, the reviewer makes a valid point that underpowering at the cluster-level analysis is a limitation of our study, which we have highlighted in the discussion. However, our estimates were relatively robust and accurate for the proportion of clusters that would be aligned with the recommendation at follow up, as well as anticipated differences between the control and intervention groups. The sample size calculation did not posit an effect size of 50% between the control and intervention groups, rather it claimed that we would have >80% power if 50% of clusters in either intervention group, and 10% of the control group clusters were aligned with the recommendation at follow up. It is this difference between groups that we were powered to detect. As it happened, nearly 40% of the knowledge broker clusters partially aligned at follow up, compared with 0% of the control clusters (representing a similar difference to our estimate).

**Reviewer 1, comment 6**: With the study being so significantly under-powered, the conclusion is worded much too strongly, that knowledge brokering is no more effective than dissemination of recommendations or standard practice. Given the lack of power to detect a difference, the only conclusion that can be drawn is that there was insufficient power to observe statistically significant differences between groups.

**Author response**: Agreed, please refer to changes in the Conclusion section of the main text (page 27, line 518-526) and Abstract (page 4, line 82-87) *“**This trial was unable to identify a difference between the knowledge broker strategy and dissemination of recommendations compared with usual practice for the promotion of evidence-informed resource allocation to inpatient weekend allied health services.”*

**Reviewer 1, comment 7**: The ceiling effect in this study requires more attention in the discussion. While a regression analysis was conducted to take into account differences at baseline with half of the knowledge brokering group already being in alignment with the recommendations, this may not adequately adjust for these baseline differences. It is also worth noting that the knowledge brokering group had more highly educated participants than the other two groups, and could this explain the baseline difference in the primary outcome?

**Author response**: The potential for ceiling effects within knowledge broker strategy is an insightful suggestion. We have added text to the Discussion section to raise this possibility, referring to the relevant extant literature (see page 24, line 439-446). Upon review of the healthcare decision maker baseline demographics table, we have identified an error in the percentages for the qualification levels in the knowledge broker group. These have now been corrected and do not appear to indicate a systematic trend towards higher levels of education. We thank the reviewer for drawing our attention to this (see Table 2, Page 17).

**Reviewer 1, comment 8**: I believe a much more developed discussion about the lessons learned about what went wrong in this trial would be extremely helpful in helping others fall in to the same challenges in evaluating knowledge brokering. This includes: appropriate designs for evaluation, a more fulsome knowledge brokering intervention implemented for a longer period of time, ensuring it is feasible to be adequately powered to detect statistically significant differences, and ensuring the outcome is not present prior to intervention. A comprehensive discussion of these issues, drawing from what is currently known in the literature, with suggestions on how to avoid these pitfalls, will provide an important contribution to the literature.

**Author response**: The discussion has been expanded in line with the reviewer’s suggested topics, please refer to the Discussion section, page 23-27.

**Reviewer 1, Minor comments**

**Reviewer 1, comment 8**: In the abstract it is stated the study occurred between Feb 2018 and Jan 2020, but in the Results section of the paper, it is stated data collection occurred until April 2020.

**Author response**: We have now provided clarification text in the Results section, page 17, line 319-320, stating *“The first cluster was recruited on the 7th of February 2018 and the final cluster completed their intervention in January 2020; final data collected for that period on the 30th of April 2020.”*

**Reviewer 1, comment 9**: Provide justification for why at the ward level, partial alignment with the recommendations was not included as an option for measuring the outcome.

**Author response**: The partial alignment classification can only apply to clusters, as it was defined by some ward types aligning and others not aligning (e.g. acute wards aligned but subacute units are not aligned, or vice versa). The ward-level analysis only considered one unit of alignment for each ward, so the partial alignment classification was not relevant or able to be applied. Description of this definition is provided in the Methods, Outcomes section, page 14.

**Reviewer 1, comment 10**: It is stated in the methods that an intention to treat analysis was conducted. However, 4 organizations either did not provide any data or follow-up data and therefore were not included in the analysis. This is not consistent with intention to treat analysis. For an intention to treat analysis to have been conducted, those organizations that did not provide data should have still been analyzed in the group to which they were allocated.

**Author response**: We agree and thank the reviewer for identifying where this could be improved. To fully align with intention to treat principles, we have performed multiple imputation (50 datasets) for missing values in both baseline and follow up alignment with the recommendations. Geographical classification was used as the independent variable when imputing missing data at the cluster level; geographical classification and ward classification were used at the ward-level. We have retained our original complete case analysis (Table 7) for sensitivity comparison with the intention to treat approach (Table 6), which demonstrates outcomes were largely unaffected. Please refer to changes in the Methods, Statistical methods, page 16, line 297-315; and Results section, page 21-22.

**Reviewer 1, comment 11**: I may have missed it but I did not see statements as to there being statistically significant differences between groups at baseline. From the data provided it appears as though there could be.

**Author response**: According to the CONSORT statement, testing for statistically significant differences between groups at baseline should not be performed. It is a practice generally discouraged because significance tests assess the probability that observed baseline differences occurred by chance; however, we already know that differences are caused by chance through random assignment. Although random assignment prevents selection bias, it does not guarantee equivalence of groups at baseline. Our comparison of baseline characteristics is based on a consideration of the prognostic impact of the variables along with the size of any chance imbalances that may have occurred.

**Reviewer 1, comment 12**: there is a formatting issue with Ref #30

**Author response**: Thank you, we have reviewed the full reference list and aligned with the journal’s Vancouver referencing style.

**Reviewer 2, comment 1**: This is an interesting cluster randomised controlled trial on the effectiveness of knowledge brokering and recommendation dissemination on resource allocation decisions. However, the trial was poorly designed and conducted with quite a few major issues needing attention.

**Author response**: We thank the reviewer for their comments and interest in our study. It is recognised that our description could have been presented more clearly and we have sought to improve the manuscript in response to the reviewer’s comments. The challenge of conducting implementation trials within real-world settings is well recognised. This trial was carefully designed and conducted considering these challenges, however we do not seek to understate its limitations. The reviewer’s comments have highlighted where we could improve our reporting and clarify potential concerns. We hope that our responses have addressed the reviewer’s concerns, and ultimately strengthened the manuscript.

**Reviewer 2, comment 2**: Cluster design. Throughout the paper, there is no detailed description of the cluster RCT design especially on cluster size. Also It's very confusing as to what was ultimately randomised? decision makers or wards?

**Author response**: We acknowledge that we could have better described our approach. In our protocol paper, we stated that clustering and randomisation to the study conditions was to occur at the organisation-level where weekend allied health staffing decisions were made. Wards were nested within these decision-making structures. For example, one organisation within our study was made up of two geographically distinct hospitals (one acute and another subacute); these hospitals made independent decisions in relation to their allied health staffing and were subsequently randomised as separate clusters. More often, allied health staffing decisions were made across all hospitals within the organisation, and subsequently multiple acute and subacute hospitals were randomised as a single cluster. The diversity in decision making structures across Australian and New Zealand hospitals meant it was impossible to anticipate how many eligible wards/hospitals would be included within each cluster, therefore cluster size could not be prespecified. Please refer to changes in the Methods, Trial design section, page 9-10, line 181-189.

**Reviewer 2, comment 3**: Sample size calculation. On page 10 and 11, it said "a sample size of 25 clusters per group..." but in the results it said 15 clusters per group. Also, cluster size or ICC was never mentioned. And, why effect size is on cluster level rather than paticipants level? In short, the sample size calculation was very confusing and not informative therefore not adequate at all.

**Author response**: The discrepancy between our required sample size at the cluster-level (25 per group) and the number of clusters recruited (14-16 per group) was a product of the unanticipated, aggregated nature of decision-making structures for allied health staffing. We found that allied health staffing decisions were made across multiple hospitals/wards within healthcare organisations, reducing the potential clusters that could be recruited and constraining our ability to reach the required sample size at the cluster-level. To provide an indication of our extensive recruitment efforts, we estimate that we captured approximately 50% of all public healthcare organisations in Australia. The cluster size (i.e. number of wards/hospitals within clusters) was unable to be pre-specified, due to the previously mentioned diversity in decision making structures across Australian and New Zealand hospitals. ICCs were not reported in the sample size calculation because the analysis at the cluster-level only involved one unit of data per cluster. We planned two levels of analysis, one to be conducted at the cluster-level (one unit of data per cluster) and the other to be conducted at the ward-level (nested within cluster). We considered the analysis at the cluster-level to be the more conservative of the two, and therefore undertook our power analysis based on this comparison. Please refer to changes to the Methods, Statistical methods section, page 16, line 306-315.

**Reviewer 2, comment 4**: Primary outcome. It says on page 9 "The primary outcome of interest was whether weekend allied health service provision at both the cluster- and ward-level aligned with the recommendations at 12-months follow up". However, wards were not in the sample size calculation so not powered therefore should not be used as a primary outcome.

**Author response**: The alignment of weekend allied health service provision with the recommendations was pre-specified within our published protocol and trial registration as a primary outcome, at both the cluster- and ward-level. Our sample size calculation was only conducted at the cluster-level as this was considered more conservative. We understand the reviewer’s concern and are prepared to change the ward-level analysis to a secondary outcome; however, this would then become a deviation from published protocol. We are also happy to conduct a post-hoc power analysis if this would be required or preferred. We respectfully seek the preference of the editor and reviewer surrounding the handling of these two options?

**Reviewer 2, comment 5**: Intention to treat. It's claimed on page 10 "Analysis was undertaken according to intention-to-treat principles...". However, as shown in Figure 1 and table 1, the randomisation was disrupted at cluster level with error and empty clusters and also at healthcare decision maker level as shown in table 1 with huge imbalance. Basically, the trial analysis was not intention to treat at all, neither at cluster level nor paticipant level. The imbalance at both levels mean the trial was poor conducted and lost the purpose of effective randomisation. It basically becomes an observational study.

**Author response**: Figure 1 has been modified to improve clarity regarding the flow of clusters. There were no empty clusters, as wards were nested within clusters. We hope the difference in hospital manager numbers between healthcare organisations (and groups) has been clarified through our response to previous comments and has been adequately reflected in modifications to the manuscript. While there was a randomisation error (one cluster did not receive their intervention) we analysed this cluster as if they received the intervention. To fully align with intention to treat principles, we have performed multiple imputation (50 datasets) for missing values in both baseline and follow up alignment with the recommendations. Geographical classification was used as the independent variable when imputing missing data at the cluster-level; geographical classification and ward classification were used at the ward-level. We have retained our original complete case analysis (Table 7) for sensitivity comparison with the intention to treat approach (Table 6), which demonstrates outcomes were largely unaffected. Please refer to changes in the Methods, Statistical methods, page 16, line 297-301; and Results sections, page 21-22.

**Reviewer 2, comment 6**: Statistical analysis. As the trial was not balanced at both levels, the simple summary statistics as shown in table 1-4 becomes inadequate and potentially misleading. Multivariable analysis adjusted for all confounders is essentially needed however it's not done or seen at all in results section although vaguely mentioned in the stats methods section. All these methodological inadequacies can lead to unreliable and unbelievable results and conclusions.

**Author response**: We acknowledge that our approach could have been better described. In response to Reviewer 2’s comments we have endeavoured to rephrase or provide additional information for clarification where possible. We do hope these clarifications have strengthened the manuscript and addressed the reviewer’s concerns. In relation to balance between study groups, a relatively even number of clusters from metropolitan and rural settings is spread across the three study conditions. We acknowledge there were differences in baseline alignment with the recommendations between groups, which was addressed by adjusting for baseline alignment within our analyses. We have added this important note regarding baseline adjustment in the Results section, page 21, line 372.

**Reviewer 2, comment 7**: Randomisation. On page 10, not clear what was randomised? clusters or healthcare managers?

**Author response**: We have modified the text to specify that hospital wards were nested within clusters, which were randomised as single units to one of the three study conditions (groups). Please see change to Methods, Randomisation, page 15, line 270-275.

**Reviewer 3, comment 1**: I wish to thank the editor for their invitation to review the manuscript, titled "Effectiveness of knowledge brokering and recommendation dissemination on resource allocation decisions: cluster randomised controlled trial." The aim of this study was to determine the effectiveness of two research implementation strategies to facilitate evidence-informed healthcare management decisions for the provision of inpatient weekend allied health services. Given that I am not a statistician and I would recommend the editors consider a statistical review prior to publication. I present my concerns in the order they appeared:

**Author response**: We thank the reviewer for their time and well considered comments.

**Reviewer 3, comment 2**: The Title: I find it a bit confusing and not specific enough

**Author response**: Thank you for identifying where we could improve the specificity of our title. We have modified our title to *“Effectiveness of knowledge brokering and recommendation dissemination for influencing healthcare resource allocation decisions: A cluster randomised controlled implementation trial”* in response to both reviewer and editorial comments.

**Reviewer 3, comment 3**: The abstract: Might want to be specific as to which were the two implementation strategies used.

**Author response**: We have added text to the Abstract to specify the two implementation strategies used, please refer to page 3, line 44-46.

**Reviewer 3, comment 4**: The background section they might want to describe in more detail what is meant by "the dissemination of evidence-based practice recommendations " (line 114-115), and the literature behind this strategy.

**Author response**: We thank the reviewer for highlighted where we could improve clarity. The dissemination of evidence-based practice recommendations is described in the previous paragraph (Introduction section, page 7, line 139-160), where we have added some text to signpost that we are referring to the strategy under investigation.

**Reviewer 3, comment 5**: How can the participants be blinded to receiving the support of a knowledge broker?

**Author response**: We agree that this would not be possible, this study is only single blinded (data collection and analysis). We have added text to the Abstract, Methods and findings section, page 3, line 50, to clarify this and align with the description in the Trial design section.

**Reviewer 3, comment 6**: How was the COM-B model used?

**Author response**: Thank you identifying where we could improve this description. The COM-B model was used to design prompting questions by the knowledge broker for the hospital managers. We have altered the text in the Methods, Interventions section, page 12, line 236-237 to specify this is how the model was used.

**Reviewer 3, comment 7**: Discussion: How do their finding relate to what previous researchers have found?

**Author response**: We have expanded our discussion in response to Reviewer 1 and Reviewer 3’s comments. These revisions include at least two paragraphs describing how our findings relate to previous research. Please refer to Discussion section, page 23-27.

**Reviewer 3, comment 8**: What are the implications to the field?

**Author response**: We have expanded our discussion in response to Reviewer 1 and Reviewer 3’s comments. Implications to the field have been provided throughout, please refer to page 23-28. (e.g. *“The implication of this research is that more intense and interactive strategies for implementing evidence into practice are not necessarily more likely to enable changes in resource allocation due to the multiple competing priorities, time and resource constraints experienced by hospital managers.”*)

**Reviewer 4, comment 1**: This study tried to address an important issue of the impact of evidence dissemination and knowledge brokering on implementation of health system evidence. It is a well designed cluster randomized trial, which is a strength. However there are a few significant shortcomings that affect the validity and usefulness of its conclusions.

**Author response**: We thank Reviewer 4 for their time and efforts providing per review for our manuscript.

**Reviewer 4, comment 2**: the interventions seem to be multi component and complex. The evidence-based recommendation states the desired objective but suggested complex activities to managers in order to achieve them. Activities such as development of a business case, identification of local priorities, engagement of diverse stakeholders including allied health professionals, nurses, and even patients in the process, and development of communication and negotiation channels with staff. It seems unlikely that email dissemination of an evidence-based guideline or one or two online webinars by the knowledge broker would be sufficient to change the behavior of hospital managers to engage in these complex and resource-intensive activities.

**Author response**: We agree with the reviewer that these implementation strategies are relatively complex interventions. This manuscript reports on the primary and secondary outcomes from this study. We plan to publish economic, process and qualitative measures, such as those described by the reviewer elsewhere. Despite its simplicity, guideline and recommendation dissemination have been found to improve awareness of key messages, change attitudes and knowledge (Campbell et al, 2011; Chambers et al, 2012; Dobbins et al, 2001), and in some circumstances impacted practice change (Grimshaw et al, 2006; Knapp et al, 2015; Salbach et al, 2017; Vedel et al, 2018). In many instances recommendation dissemination has not led to meaningful changes in behaviour. Yet, given the feasibility and low cost it may be an efficient way to change practice under certain circumstances, even if it is only minimally effective. Therefore, we hypothesised it may be a reasonable strategy to compare with the more active knowledge broker. The knowledge broker involved more than one or two group webinars. It involved: 1) Initial individualised contact made via email or phone to confirm receipt of the written recommendations, discuss local needs, and discuss a plan over the next 12-months; 2) within 6-months (according to hospital manager availability) a group webinar was arranged; 3) the group webinar was followed up by individualised contact via email or phone (according to hospital manager preference); 4) a final group webinar was arranged; 5) follow up individualised contact thereafter on an “as needs” basis. The dose or contacts made over a 12-month period varied according to levels of participant engagement. Changes have been made to the Methods, Interventions section, page 11-14, to improve this description.

**Reviewer 4, comment 3**: Since the authors did not report any information regarding the adoption and implementation of such activities (e.g how many managers initiated those activities, how many actually implemented them in their routine practice, how many were successful in engaging staff in planning and priority setting, etc), it is difficult to attribute the study findings to the lack of effectiveness or unsuccessful implementation. This is a widespread shortcoming in implementation studies, where the emphasis is on comparative effectiveness rather than investigating the conditions that facilitate or impede utilization and ultimate effectiveness of an implementation strategy. Ideally, a mixed methods study would provide better opportunities to address the contextual and procedural complexities of these interventions. But even as a proper RCT, a more comprehensive assessment of implementation outcomes was needed.

**Author response**: We have modified the text to link our outcomes to the implementation outcome taxonomy. The primary outcome (practice alignment with recommendations) can be conceptualised as addressing three implementation outcomes: adoption of evidence-based practice recommendation, penetration among eligible hospital wards, and fidelity to the recommendation (please refer to Methods, Outcome section, page 14, line 250-252). Further economic (implementation cost), process and qualitative measures are planned to be published in other manuscripts (e.g. perceptions of the trustworthiness and sufficiency of evidence of the evidence base to guide clinical practice in this area of allied health service delivery; the sources of information relied upon by allied health managers when deciding upon the model of weekend allied health service delivery they used in acute and sub-acute hospital wards and why they chose to use those sources; perceived most influential source of information encountered by allied health managers when deciding upon the model of weekend allied health service delivery they used in acute and sub-acute hospital wards and why they thought this source was the most influential; perceived potential improvements to the intervention received and how it was provided; perceived risks, barriers, and facilitators to adopting evidence-based policy recommendations).

**Reviewer 4, comment 4**: ward-level analysis of alignment to the guidelines at study arms (table 4) shows that the number of wards with missing status increased dramatically at follow up in knowledge broker and dissemination arms. This along with the lack of engagement of sites in knowledge broker meetings implies that there were challenges in preserving the adherence of hospital mangers to the interventions. More clarification is needed regarding the potential reasons for this lack of adherence and the increase in non-response in intervention arms.

**Author response**: An explanatory sentence for reasons data were missing has been provided in the Results section, page 19, line 348-351 *“The most common reasons for missing follow up data across all three groups were 1) that a different contact person was used (due to staff turnover) who did not know how to extract all the required data; 2) the contact person did not have time to extract all the required data.”* It appears the increase in missing data was primarily for acute wards, whereas subacute wards did not have a large difference. This indicates it might not be related to the interventions but rather challenges collecting data from acute services. Reasons for reduced engagement with the knowledge broker is provided in the Results, Process outcomes section, page 23, line 411-414 *“A desire to make an internal decision regarding weekend allied health service provision was the most common reason for nonparticipation. Two clusters (14%) did not engage with the knowledge broker without providing a reason.”*
