# Supplementary material for: Effectiveness of knowledge brokering and recommendation dissemination for influencing healthcare resource allocation decisions: A cluster randomised controlled implementation trial
Source: PLoS Med. 2021 Oct 22;18(10):e1003833. doi: 10.1371/journal.pmed.1003833 (PMC8570499; doi:10.1371/journal.pmed.1003833)
Supplement: Attachment — Submitted filename: Response to reviewer comments 230721.docx [file pmed.1003833.s008.docx]

**Response to reviewer comments**

**Effectiveness of knowledge brokering and recommendation dissemination for influencing healthcare resource allocation decisions: A cluster randomised controlled implementation trial (PMEDICINE-D-21-01199R3)**

The authorship team would like to extend our appreciation to the editor and peer reviewers for considering our manuscript revision. We have responded to each reviewer comment and made modifications where required. Page and line numbers in each response refer to the “tracked” version of the manuscript.

**Comments from the reviewers**

**Reviewer #1, comment 1:** The authors have comprehensively addressed all of my suggestions.

**Author response**: We thank the reviewer for their expertise and peer review for our manuscript.

**Reviewer #2, comment 1:** Many thanks authors for their great effort to improve the manuscript. I can appreciate the challenges in this implementation trial that investigators have faced and the fact that investigators tried their best to deal with the situations, however, I am still not convinced/statisfied with the response and revision. Remaing issues:

**Author response**: We wish to extend our thanks and appreciation to the reviewer for their expertise, time, and effort providing peer review for our manuscript. We believe the manuscript has been greatly strengthened because of the reviewer’s suggestions.

**Reviewer #2, comment 2:** This is not an "intention to treat" analysis. There are 3 clusters without 12-month follow up (primary) so not in the main analysis, as shown in Figure 1. Also multiple imputation for missing data is not ITT analysis. So far, it was effectively performed with a complete case analysis plus sensitivity analysis for missing values (imputation).

**Author response**: We have removed the term ‘intention to treat’ from the manuscript and instead described our approach to handling missing data. Data were imputed for the clusters with missing follow up data, so they could be included in the primary analysis. Figure 1 has been modified to show which data were included in each analysis, for each group (collected or imputed). There is currently no universal consensus on how to conduct an ITT analysis in the presence of missing outcome data (Alshurafa et al 2012). We believe that our approach to handling missing data, using multiple imputation as our primary analysis, is consistent with the CONSORT Statement (31/07/2013): *“A strategy for analyzing data in which all participants are included in the group to which they were assigned, whether or not they completed the intervention given to the group.”*

Please refer to changes to Figure 1; Trial design section, page 9, line 195-196; Statistical methods section, page 15, line 291-292; and Results section, page 21, line 403.

- REF: Alshurafa M, Briel M, Akl EA, Haines T, Moayyedi P, et al. (2012) Inconsistent Definitions for Intention-To-Treat in Relation to Missing Outcome Data: Systematic Review of the Methods Literature. *PLOS ONE* 7(11): e49163. https://doi.org/10.1371/journal.pone.0049163

**Reviewer #2, comment 3:** Design issue. Cluster RCT without pre-specified or uncertain cluster size? Sample size only for cluster level but not for ward level? Primary outcome for both cluster (powered) and ward (not powered)? All these deviate from the definition of a cluster RCT. Perhaps this is the pragmatic nature of implementation trial but how to address the methodological issues and potential biases which could impact on the results?

**Author response**: It is appropriate to analyse cluster RCTs at the cluster-level summary or individual-level data (Hayes & Moulton 2017). We decided to power our study at the cluster-level because we anticipated this to be the more conservative option than at the ward-level. Thus, an adequate sample size at the cluster-level would also prove sufficient at the ward-level. This decision, and the sample size calculation was published in the trial protocol (Sarkies et al 2017). A further sample size calculation was not conducted at the ward-level because there was no reasonable way to estimate the likely ICC for this healthcare context and our main sample size constraints pertained to the practicality of recruitment at the cluster-level. During protocol design and sample size calculation, it was not possible to anticipate how many wards would be included in each cluster prior to recruitment, as we first needed to ascertain the decision-making structure for each healthcare organisation in order to understand how many wards were nested within each decision-making structure (e.g. single hospital or network of hospitals). We acknowledge that our efforts to understand and cluster according to these decision-making structures prevented pre-specification of cluster size. This design element was imperative to avoid the potential risk of contamination between units of randomisation. We do not believe the pragmatic nature of this implementation trial introduced potential sources of bias that could impact on the results, rather these limitations were introduced through efforts to reduce the risk of contamination bias. This additional justification for our approach has been added to the Methods and Discussion sections.

We understand the reviewer’s concern. It would be possible to change the ward-level analysis to a secondary outcome; however, this would then become a deviation from published protocol. We are also happy to conduct a post-hoc power analysis at the ward-level if this would be required.

Please refer to changes in the Trial design section, page 8, line 180-181; Statistical methods section, page 15, line 306-312; and Discussion section, page 26, line 505-512.

- REF: Hayes, R. J., & Moulton, L. H. (2017). Cluster randomised trials. Chapman and Hall/CRC Press, Boca Raton, FL.
- REF: Sarkies, M. N., White, J., Morris, M. E., Taylor, N. F., Williams, C., O’Brien, L., ... & Haines, T. P. (2018). Implementation of evidence-based weekend service recommendations for allied health managers: a cluster randomised controlled trial protocol. *Implementation Science*, 13(1), 1-14. https://doi.org/10.1186/s13012-018-0752-7

**Reviewer #2, comment 4:** Statistical analysis. Again, the trial was not balanced at both levels, the simple summary statistics becomes inadequate and potentially misleading. Multivariable analysis comprehensively adjusted for all confounders is essentially needed. Otherwise, the results may be subject to scrutiny due to design, sample size and trial conduct issues.

**Author response**: Thank you for the suggestion. We have added a sensitivity multivariable analysis to adjust for other potential baseline confounders (cluster-level: geography and full-time research staffing; ward-level: geography and ward type). Findings were similar with the addition of other potential confounders, although a significant reduction in hospital length of stay was observed for the knowledge broker group when compared to the control. This finding indicates there might have been an effect, but the precise mechanism is unclear without an observed change in recommendation alignment. This additional analysis has been added to the Methods and Results sections, and we have elaborated on the findings in the Discussion.

The random allocation of clusters to groups was relatively balanced in this trial (Control: 15; Recommendation: 16; Knowledge broker: 14). There was also a relatively even number of clusters from metropolitan and rural settings spread across the three groups, achieved through stratification. The difference in ward numbers nested within these clusters is a product of the variability in allied health staffing decision making structures for each cluster, which was caused by chance through random assignment. Although random assignment prevents selection bias, it does not guarantee equivalence of groups at baseline. We acknowledge there were differences in baseline alignment with the recommendations between groups and have explored this potential limitation and our approach for adjusting for baseline alignment in the Discussion.

Please refer to changes in the Statistical methods section, page 15, line 297-301; and Results section, page 20-21, line 378-400.

**Reviewer #3, comment 1:** The authors have addressed all the comments in a satisfactory manner.

**Author response**: We thank the reviewer for their expertise and peer review for our manuscript.

**Reviewer #4, comment 1:** The authors tried to address most of the comments by reviewers. The revised manuscript is better organized and is more informative.

**Author response**: We thank the reviewer for their time and efforts providing informative comments, which we feel has led to an improved manuscript.

**Reviewer #4, comment 2:** My main remaining concern, which is now more prominent since the term 'implementation' was added to the title, is lack of a guiding framework and evaluation metrics related to the implementation. Alignment of practice with the guidelines does not capture the complexities of the implementation. It seems that some data have already been collected that should be added to the Results. For example webinar attendance rates at ward and organization levels may address the notion of 'adoption' of the intervention by managers. Furthermore, the authors can include ward-level attendance data in the regression analyses to assess its impact on study outcomes.

**Author response**: The reviewer makes an important and relevant comment in relation to reporting implementation processes thought to drive our effectiveness outcomes. Webinar attendance rates are reported at an aggregate level in the Process outcomes section, page 23, line 419-433. We are unable to report attendance at the cluster level, as this would potentially enable re-identification of research participants and breach the conditions of our ethical approval. Attendance cannot be reported at the ward-level, as allied health staffing decisions were made at the cluster-level (i.e., webinar attendees were representing an entire hospital or networks of hospitals). We are confident that the qualitative and economic outcome data collected throughout this trial is better placed to explain implementation processes than more detailed information about webinar attendance rates, and have included statements in the Methods outcomes section, page 13, line 263-264 and Process outcomes section, page 23, line 434-436:

“Detailed process and economic outcomes described in our protocol are planned for other publications.”

“Further detail regarding the knowledge broker webinar support sessions is published elsewhere [58]. Other process, economic and qualitative outcomes specified in our protocol are planned to be reported in other publications.”

Our primary outcome was to determine whether these implementation strategies worked (i.e., the effectiveness of knowledge brokering and dissemination of recommendations). While we did not frame our study using RE-AIM, there were several other frameworks guiding the evaluation and reporting within the manuscript. Our evaluation was guided by the CONSORT Extension for Cluster Trials checklist and Standards for Reporting Implementation Studies statement (StaRI). The implementation strategies were reported according to the Template for Intervention Description and Replication (TIDieR) guidelines and in line with Proctor’s framework for the specification of implementation strategies. These strategies were based on factors perceived to be associated with implementation success from a recent systematic review, and specifically the knowledge broker prompting questions were informed by the COM-B (Capability, Opportunity, Motivation, and Behaviour) model. Primary, secondary and process outcomes used in this study have also been mapped to Proctor’s implementation outcomes taxonomy. Our approach for examining implementation processes follows a Donabedian model for linking structure (strategies), process (includes webinar attendance but primarily qualitative to be reported elsewhere), and outcome (primary and secondary). The webinar attendance rates were only available for clusters in the knowledge broker group and our experience suggests that qualitative explorations of the decision-making process provide a more nuanced insight to the factors influencing adoption of the intervention (alignment with the recommendations). These qualitative explorations have been reserved for other publications.

**Reviewer #4, comment 3**: Also, given the large non-response, it is important to provide some contextual information about the characteristics of non-respondents, in comparison to the participating organizations. This comparison may reflect the notion of 'reach' (who was missed?), using RE-AIM terminology.

**Author response**: We thank the reviewer for bringing our attention to the notion of reach and how it applies to implementation trials. Missing data in the study did not necessarily infer non-response, as the primary reason for missing data was *“1) that a different contact person was used (due to staff turnover) who did not know how to extract all the required data; 2) the contact person did not have time to extract all the required data.”* (Please refer to the Results section, page 18, line 350-353). Unfortunately, we are also unable to report the characteristics of organisations with higher and lower knowledge broker engagement, as this would also potentially enable re-identification of research participants and breach the conditions of our ethical approval. We also believe reporting summative attendance rates according to organisational characteristics would be misleading, as this study was not powered to explore potential associations between these variables. Questions surrounding the reach of our strategies are better answered with qualitative data reserved for other publications, indicated in the Methods outcomes section, page 13, line 263-264 and Process outcomes section, page 23, line 434-436. The primary aim of this paper is to report on the effectiveness of the strategies.

**Reviewer #4, comment 4:** Providing some temporal participation and adoption statistics (how did the participation differ during the first few months vs later dates), if available, would address the notion of sustainability ('maintenance' in RE-AIM)

**Author response**: We agree with the reviewer’s suggestion as it pertains to the knowledge broker webinar attendance. Engagement was much higher in the initial webinars (range 6-18) compared to the follow up webinars (range 2-8). Typically, a larger pool of allied health decision makers attended the first webinars; however, the second webinars were dominated by decision makers from the physiotherapy and occupational therapy professions because the evidence and recommendations mostly pertained to these professions, leading to lower attendance rates. We have added this detail in the Process outcomes section.

Please refer to changes in the Process outcomes section, page 23, line 427-431.
